# Supplementary material for: Altered microRNA Transcriptome in Cultured Human Liver Cells upon Infection with Ebola Virus
Source: Int J Mol Sci. 2021 Apr 6;22(7):3792. doi: 10.3390/ijms22073792 (PMC8038836; doi:10.3390/ijms22073792)
Supplement: Supplementary file 1 [file ijms-22-03792-s001.zip › Supplementary_File/C_ GO_Analysis_Results/16-30nt_go_Mayinga-96h-Huh7_vs_Control-96h-Huh7_down.mature_mirna_targets/BP_result(Human).html]

| GO.ID | Term | Ontology | Count | Pop.Hits | List.Total | Pop.Total | Fold.Enrichment | Pvalue | FDR | Enrichment.Score | Gene.Ratio | GENES |
| --- | --- | --- | --- | --- | --- | --- | --- | --- | --- | --- | --- | --- |
| GO:0007399 | nervous system development | Biological process | 74 | 2296 | 372 | 17653 | 1.52945047019595 | 0.000108414834785356 | 0.32079239188053 | 3.96491128768127 | 0.198924731182796 | FBXO45//SATB2//MARK1//ASCL1//NDN//FZD3//TMED2//OTP//LRRC38//FGFR2//RTN4//LRRN1//PARD6B//EFNA3//NCAM1//SPTBN2//CNTN2//VLDLR//UNC5A//NLGN1//DBNL//BSN//NPTX1//PAX8//CTNS//FOXG1//GRIN2B//H3F3B//CA10//BCR//SLC5A3//SFRP1//ZNF804A//TRIM67//RIT2//BAG5//NAB1//RND1//LRP8//PAX5//SYPL2//MAS1//DLX5//DAPK3//FZD2//PROX2//TTL//GNAO1//JAM3//NREP//UBE4B//TBC1D24//PRDM12//KCNIP2//HEYL//KIT//TLX2//HS6ST1//PPP3CB//SEMA4G//LRRTM1//PALM//PLAG1//ABCC8//ACTR2//ZSWIM6//CHRM1//ELAVL3//FGF11//KIF2A//NDP//SCN3B//PURA//SMPD1// |
| GO:0048666 | neuron development | Biological process | 41 | 1060 | 372 | 17653 | 1.83549655102455 | 0.000127684006895258 | 0.32079239188053 | 3.89386349703662 | 0.110215053763441 | ASCL1//LRRC38//FGFR2//RTN4//LRRN1//PARD6B//EFNA3//NCAM1//SPTBN2//CNTN2//VLDLR//UNC5A//NDN//SFRP1//ZNF804A//TRIM67//RIT2//BAG5//RND1//FBXO45//TTL//JAM3//NREP//UBE4B//NLGN1//GNAO1//TBC1D24//PRDM12//FZD3//KCNIP2//PPP3CB//DBNL//SEMA4G//TLX2//FZD2//NPTX1//ACTR2//LRP8//DLX5//ZSWIM6//HS6ST1// |
| GO:0015012 | heparan sulfate proteoglycan biosynthetic process | Biological process | 5 | 25 | 372 | 17653 | 9.49086021505376 | 0.000151817809376872 | 0.32079239188053 | 3.81867727942588 | 0.0134408602150538 | NDST3//HS6ST1//EXTL2//XYLT2//NDST2// |
| GO:0065007 | biological regulation | Biological process | 290 | 12351 | 372 | 17653 | 1.11422130287652 | 0.000302442632727861 | 0.32079239188053 | 3.51935699011053 | 0.779569892473118 | ZNF268//EDN1//FGFR2//SATB2//ZNF281//GABPA//HEYL//PROX2//KCNIP3//ASCL1//NFKB1//PAX5//FOXP3//GATAD2B//UBE2D3//VLDLR//LRP8//BHLHE40//PAXBP1//CAMKK2//GRIN2B//IL5RA//KIT//NCAM1//MAPK4//PSMC6//SPTBN2//YWHAB//H3F3B//PRKAA1//THBS1//CD81//EIF4A2//PAN3//PTBP1//SGK2//IGFBP7//ABCC8//JAM3//EHD3//REP15//TP53INP2//PPP3CB//MAS1//CDK2AP1//SFRP1//TSLP//OTP//FZD3//NLGN1//LRRTM1//CD209//RAG1//IL4R//CREB3L3//BCR//FFAR3//NDFIP1//P2RX2//CHRM1//DDAH1//CYP4F2//LIN9//NUP214//AKAP10//VPS45//C1QA//TRABD2B//MARK1//NDP//TNKS2//VPS26A//FZD2//GNAO1//RALBP1//PRDM4//PTPRT//TAGAP//FGF11//GABRR1//HTR3E//GRP//KCNIP2//ARHGAP40//IGFBP1//LGALS3BP//NDFIP2//HIVEP3//RASL11B//SMPD1//RERGL//OGT//TNFSF14//SH3BP5//CXCL14//GNA14//NUDT4//DAPK3//CARHSP1//RIT2//OXSR1//PPP1R3F//TXNDC8//TMX1//MCIDAS//ZNF618//ZNF519//ZNF781//EWSR1//ATP1B4//MYCBP//HMGB3//NAB1//NFYA//PFDN1//ASF1B//GPBP1L1//SNAPC1//ZNF17//ZNF322//ZNF468//ZNF682//ELK1//FOXG1//HOXC9//PTAFR//PURA//CREBZF//PRDM12//CDC5L//EIF1B//EIF4G2//CEP85//KDELR1//SYPL2//LETM2//TLN1//GAS2//KMT2E//ZW10//TTK//RND1//CLEC1A//TSPAN14//TSPAN18//PIK3C2A//VAV3//GNAZ//GPR34//CYSLTR2//CCL16//ARHGEF6//GPR3//PALM//GRIA3//NPVF//EVC//ITGA5//FERMT3//DBNL//ARFIP2//RHOF//RALGPS2//CPLX1//MAEA//TTC28//SYT9//BRK1//DDX39B//UFM1//RGS5//LDOC1//ASPH//NDN//EEF1E1//ATP6V1G2//PARVA//POLR2E//UBE4B//CASP7//PYURF//MAPRE1//SCN3B//BMP10//IL26//PIP4K2C//TRIM21//PPP4R2//TMED2//RBMS3//ZNF804A//PLAG1//RTN4//PAX8//CTNS//MICAL2//AKAP7//MEST//INSIG2//PPP1R26//CNTN2//TRIM67//BAG5//PIK3IP1//CPLX3//ARPP19//LARP1//MGRN1//SERBP1//TNFAIP8L2//ANP32E//SEPT7//EFNA3//TAF8//DLX5//PASD1//NREP//MEX3B//CYP7A1//SCN1A//DPYS//HLA-DPA1//KIF2A//PARD6B//SEMA4G//TTL//CTH//EDEM3//EXD1//SKA3//UBQLN1//USP46//CA7//RNF135//RABL3//RAB2A//TBC1D24//WDFY1//ARPC3//ACTR2//KCNA6//CACNG1//KCNQ4//C9ORF129//MTAP//VSNL1//EXTL2//GOSR2//UNC5A//PTPN18//CPS1//SLC26A1//SLC1A6//WDR91//SLC5A3//UBXN2B//PPIH//FAM57B//ERH//TET3//TLX2//TMPRSS2//CLEC9A//CD300E//CGNL1//IFRD2//NOVA1//XRCC4//SLC9B1//BEST1//LRRN1//STEAP2//SLC30A6//ANO3//HAAO//CANX//GRIPAP1//BSN//LYPD6//PATE1//IARS//LSM11//SNRPA//PEX5//ALAD//ZSWIM6//NUCB1//LRRC38//SESTD1//FAM168A// |
| GO:0048699 | generation of neurons | Biological process | 50 | 1437 | 372 | 17653 | 1.65115870129676 | 0.000302509212077443 | 0.32079239188053 | 3.51926139557851 | 0.134408602150538 | FBXO45//SATB2//MARK1//ASCL1//NDN//FZD3//OTP//LRRC38//FGFR2//RTN4//LRRN1//PARD6B//EFNA3//NCAM1//SPTBN2//CNTN2//VLDLR//UNC5A//SFRP1//ZNF804A//TRIM67//RIT2//BAG5//RND1//DLX5//DAPK3//FZD2//PROX2//TTL//JAM3//NREP//UBE4B//NLGN1//GNAO1//TBC1D24//PRDM12//KCNIP2//HEYL//KIT//HS6ST1//PPP3CB//DBNL//SEMA4G//TLX2//PLAG1//ABCC8//NPTX1//ACTR2//LRP8//ZSWIM6// |
| GO:0070542 | response to fatty acid | Biological process | 8 | 81 | 372 | 17653 | 4.68684455064383 | 0.000303740003422713 | 0.32079239188053 | 3.51749800649584 | 0.021505376344086 | EDN1//PRKAA1//SFRP1//FFAR3//PTAFR//CPS1//ALAD//INSIG2// |
| GO:0031175 | neuron projection development | Biological process | 36 | 936 | 372 | 17653 | 1.82516542597188 | 0.000371593412925613 | 0.32079239188053 | 3.42993199315906 | 0.0967741935483871 | LRRC38//FGFR2//RTN4//LRRN1//PARD6B//EFNA3//NCAM1//SPTBN2//CNTN2//VLDLR//UNC5A//NDN//SFRP1//ZNF804A//TRIM67//RIT2//BAG5//FBXO45//TTL//JAM3//NREP//FZD3//PPP3CB//DBNL//SEMA4G//TLX2//NPTX1//NLGN1//ACTR2//LRP8//DLX5//ZSWIM6//UBE4B//GNAO1//TBC1D24//PRDM12// |
| GO:0030182 | neuron differentiation | Biological process | 46 | 1303 | 372 | 17653 | 1.67528614693965 | 0.000392169040342646 | 0.32079239188053 | 3.40652669455163 | 0.123655913978495 | ASCL1//LRRC38//FGFR2//RTN4//LRRN1//PARD6B//EFNA3//NCAM1//SPTBN2//CNTN2//VLDLR//UNC5A//NDN//SFRP1//ZNF804A//TRIM67//RIT2//BAG5//RND1//OTP//DLX5//SATB2//FBXO45//TTL//JAM3//NREP//UBE4B//NLGN1//GNAO1//TBC1D24//PRDM12//FZD3//KCNIP2//HEYL//HS6ST1//PPP3CB//DBNL//SEMA4G//TLX2//FZD2//NPTX1//ACTR2//LRP8//ZSWIM6//DAPK3//PROX2// |
| GO:0030201 | heparan sulfate proteoglycan metabolic process | Biological process | 5 | 31 | 372 | 17653 | 7.65391952826916 | 0.000437667278160374 | 0.32079239188053 | 3.35885592184841 | 0.0134408602150538 | EXTL2//XYLT2//NDST2//NDST3//HS6ST1// |
| GO:0007267 | cell-cell signaling | Biological process | 55 | 1658 | 372 | 17653 | 1.5741776593123 | 0.000481684391838592 | 0.32079239188053 | 3.31723742602975 | 0.147849462365591 | TRABD2B//MARK1//NDP//PRKAA1//TNKS2//VPS26A//FZD2//GNAO1//PPP3CB//FZD3//NLGN1//CPLX1//GABRR1//HTR3E//GRIN2B//KCNIP2//NOVA1//NPTX1//RIT2//SLC1A6//BSN//CHRM1//P2RX2//SYT9//ABCC8//CPLX3//EDN1//FOXP3//FGFR2//SFRP1//USP46//CA7//NPVF//GRIA3//VSNL1//CNTN2//KIT//LRP8//PSMC6//LRRTM1//SCN3B//RBMS3//IGFBP1//DAPK3//DLX5//NFKB1//FFAR3//GRP//PAX8//EFNA3//FGF11//IL26//CCL16//C1QA//CXCL14// |
| GO:0006936 | muscle contraction | Biological process | 18 | 355 | 372 | 17653 | 2.40613357564743 | 0.00056723756832153 | 0.32079239188053 | 3.2462350134131 | 0.0483870967741935 | CHRM1//DAPK3//MYOM3//EDN1//PIK3C2A//P2RX2//BMP10//EHD3//SCN3B//SCN1A//KIT//PTAFR//GNAO1//KCNIP2//ASPH//TLN1//CACNG1//SSPN// |
| GO:0120036 | plasma membrane bounded cell projection organization | Biological process | 49 | 1444 | 372 | 17653 | 1.61029137997796 | 0.000613164599530915 | 0.32079239188053 | 3.21242292666858 | 0.131720430107527 | LRRC38//FGFR2//RTN4//LRRN1//PARD6B//EFNA3//NCAM1//SPTBN2//CNTN2//VLDLR//UNC5A//NDN//ACTR2//UBXN10//EHD3//MCIDAS//PARVA//SEPT7//BRK1//SFRP1//ZNF804A//TRIM67//RIT2//BAG5//FBXO45//VAV3//ARFIP2//KIT//ARHGEF6//TTL//JAM3//NREP//UBE4B//NLGN1//GNAO1//TBC1D24//PRDM12//FZD3//PPP3CB//DBNL//SEMA4G//TLX2//PALM//NPTX1//LRP8//DLX5//MAPRE1//ZSWIM6//GRIN2B// |
| GO:0022008 | neurogenesis | Biological process | 51 | 1536 | 372 | 17653 | 1.57563109038978 | 0.000771179247103484 | 0.32079239188053 | 3.11284466607321 | 0.137096774193548 | FBXO45//SATB2//MARK1//ASCL1//NDN//FZD3//OTP//LRRC38//FGFR2//RTN4//LRRN1//PARD6B//EFNA3//NCAM1//SPTBN2//CNTN2//VLDLR//UNC5A//SFRP1//ZNF804A//TRIM67//RIT2//BAG5//NAB1//RND1//DLX5//DAPK3//FZD2//PROX2//TTL//JAM3//NREP//UBE4B//NLGN1//GNAO1//TBC1D24//PRDM12//KCNIP2//HEYL//KIT//HS6ST1//PPP3CB//DBNL//SEMA4G//TLX2//PLAG1//ABCC8//NPTX1//ACTR2//LRP8//ZSWIM6// |
| GO:0042471 | ear morphogenesis | Biological process | 9 | 117 | 372 | 17653 | 3.65033085194376 | 0.000831774032079942 | 0.32079239188053 | 3.07999464239911 | 0.0241935483870968 | FGFR2//DLX5//INSIG2//BCR//PAX8//FZD3//KCNQ4//EDN1//FZD2// |
| GO:0021879 | forebrain neuron differentiation | Biological process | 6 | 53 | 372 | 17653 | 5.37218502738892 | 0.000837286835648787 | 0.32079239188053 | 3.07712573698953 | 0.0161290322580645 | CNTN2//FGFR2//DLX5//ASCL1//SATB2//OTP// |
| GO:0071398 | cellular response to fatty acid | Biological process | 6 | 53 | 372 | 17653 | 5.37218502738892 | 0.000837286835648787 | 0.32079239188053 | 3.07712573698953 | 0.0161290322580645 | PRKAA1//SFRP1//CPS1//EDN1//FFAR3//PTAFR// |
| GO:0050789 | regulation of biological process | Biological process | 275 | 11709 | 372 | 17653 | 1.11452154716021 | 0.00085692343018029 | 0.32079239188053 | 3.06705798243599 | 0.739247311827957 | ZNF268//EDN1//FGFR2//SATB2//ZNF281//GABPA//HEYL//PROX2//KCNIP3//ASCL1//NFKB1//PAX5//FOXP3//GATAD2B//UBE2D3//VLDLR//LRP8//BHLHE40//PAXBP1//CAMKK2//GRIN2B//IL5RA//KIT//NCAM1//MAPK4//PSMC6//SPTBN2//YWHAB//H3F3B//PRKAA1//THBS1//CD81//EIF4A2//PAN3//PTBP1//SGK2//IGFBP7//EHD3//REP15//PPP3CB//MAS1//CDK2AP1//SFRP1//TSLP//OTP//FZD3//NLGN1//LRRTM1//CD209//RAG1//IL4R//CREB3L3//BCR//FFAR3//NDFIP1//CHRM1//LIN9//NUP214//C1QA//TRABD2B//MARK1//NDP//TNKS2//VPS26A//FZD2//GNAO1//RALBP1//PRDM4//PTPRT//AKAP10//TAGAP//FGF11//GABRR1//HTR3E//GRP//KCNIP2//ARHGAP40//IGFBP1//LGALS3BP//NDFIP2//HIVEP3//RASL11B//SMPD1//ABCC8//RERGL//OGT//TNFSF14//SH3BP5//CXCL14//GNA14//NUDT4//DAPK3//CARHSP1//RIT2//OXSR1//PPP1R3F//TXNDC8//TMX1//MCIDAS//ZNF618//ZNF519//ZNF781//EWSR1//ATP1B4//MYCBP//HMGB3//NAB1//NFYA//PFDN1//ASF1B//GPBP1L1//SNAPC1//ZNF17//ZNF322//ZNF468//ZNF682//ELK1//FOXG1//HOXC9//PTAFR//PURA//CREBZF//PRDM12//CDC5L//EIF1B//EIF4G2//CEP85//GAS2//KMT2E//ZW10//TTK//RND1//CLEC1A//TSPAN14//TSPAN18//PIK3C2A//VAV3//GNAZ//GPR34//CYSLTR2//CCL16//ARHGEF6//GPR3//PALM//GRIA3//NPVF//EVC//ITGA5//TLN1//FERMT3//DBNL//DDAH1//ARFIP2//RHOF//RALGPS2//MAEA//TTC28//SYT9//BRK1//DDX39B//UFM1//RGS5//LDOC1//ASPH//NDN//EEF1E1//ATP6V1G2//PARVA//POLR2E//UBE4B//CASP7//PYURF//MAPRE1//SCN3B//BMP10//IL26//PIP4K2C//TP53INP2//TRIM21//P2RX2//PPP4R2//TMED2//RBMS3//ZNF804A//PLAG1//RTN4//PAX8//CTNS//MICAL2//AKAP7//MEST//INSIG2//PPP1R26//CNTN2//TRIM67//BAG5//PIK3IP1//ARPP19//LARP1//MGRN1//SERBP1//TNFAIP8L2//ANP32E//SEPT7//EFNA3//TAF8//DLX5//PASD1//NREP//MEX3B//CPLX1//CYP7A1//HLA-DPA1//KIF2A//PARD6B//SEMA4G//TTL//CTH//EDEM3//EXD1//SKA3//UBQLN1//USP46//CA7//CYP4F2//RNF135//RABL3//RAB2A//WDFY1//ARPC3//ACTR2//KCNA6//SCN1A//CACNG1//KCNQ4//C9ORF129//MTAP//VSNL1//EXTL2//GOSR2//UNC5A//PTPN18//WDR91//SLC5A3//UBXN2B//PPIH//FAM57B//ERH//TET3//TLX2//TMPRSS2//CPLX3//CLEC9A//CD300E//CGNL1//IFRD2//NOVA1//BEST1//LRRN1//SLC30A6//CANX//JAM3//BSN//LYPD6//PATE1//LSM11//SNRPA//PEX5//ALAD//ZSWIM6//NUCB1//LRRC38//SESTD1//FAM168A//GRIPAP1// |
| GO:0061564 | axon development | Biological process | 22 | 497 | 372 | 17653 | 2.10059280413665 | 0.00090713878397846 | 0.32079239188053 | 3.04232626475339 | 0.0591397849462366 | LRRC38//FGFR2//RTN4//LRRN1//PARD6B//EFNA3//NCAM1//SPTBN2//CNTN2//VLDLR//UNC5A//NDN//FBXO45//TTL//JAM3//NREP//FZD3//PPP3CB//SEMA4G//NPTX1//DLX5//ZSWIM6// |
| GO:0048667 | cell morphogenesis involved in neuron differentiation | Biological process | 24 | 567 | 372 | 17653 | 2.00864766456164 | 0.000999285322858314 | 0.32079239188053 | 3.00031049130272 | 0.0645161290322581 | LRRC38//FGFR2//RTN4//LRRN1//PARD6B//EFNA3//NCAM1//SPTBN2//CNTN2//VLDLR//UNC5A//FBXO45//TTL//FZD3//NDN//PPP3CB//SEMA4G//TLX2//NPTX1//NLGN1//ACTR2//LRP8//DLX5//ZSWIM6// |
| GO:0030030 | cell projection organization | Biological process | 49 | 1477 | 372 | 17653 | 1.57431330581461 | 0.00100237554893806 | 0.32079239188053 | 2.9989695356799 | 0.131720430107527 | LRRC38//FGFR2//RTN4//LRRN1//PARD6B//EFNA3//NCAM1//SPTBN2//CNTN2//VLDLR//UNC5A//NDN//ACTR2//UBXN10//EHD3//MCIDAS//PARVA//SEPT7//BRK1//SFRP1//ZNF804A//TRIM67//RIT2//BAG5//FBXO45//VAV3//ARFIP2//KIT//ARHGEF6//TTL//JAM3//NREP//UBE4B//NLGN1//GNAO1//TBC1D24//PRDM12//FZD3//PPP3CB//DBNL//SEMA4G//TLX2//PALM//NPTX1//LRP8//DLX5//MAPRE1//ZSWIM6//GRIN2B// |
| GO:0032989 | cellular component morphogenesis | Biological process | 39 | 1101 | 372 | 17653 | 1.68094254490053 | 0.00103871275836916 | 0.32079239188053 | 2.98350453396568 | 0.104838709677419 | TXNDC8//LRRC38//FGFR2//RTN4//LRRN1//PARD6B//EFNA3//NCAM1//SPTBN2//CNTN2//VLDLR//UNC5A//DAPK3//GAS2//RND1//KIT//PALM//RHOF//PARVA//SEPT7//FBXO45//MYOM3//TTL//FERMT3//FZD3//BMP10//NDN//PPP3CB//DBNL//SEMA4G//TLX2//EDN1//NPTX1//NLGN1//ACTR2//LRP8//PROX2//DLX5//ZSWIM6// |
| GO:0016482 | cytosolic transport | Biological process | 10 | 146 | 372 | 17653 | 3.25029459419649 | 0.00107142882868264 | 0.32079239188053 | 2.97003667234682 | 0.0268817204301075 | TGOLN2//SYS1//EHD3//YKT6//AP1S1//VPS53//VPS26A//GOSR2//WDR91//ACTR2// |
| GO:0042472 | inner ear morphogenesis | Biological process | 8 | 98 | 372 | 17653 | 3.87382049594031 | 0.00109096924637767 | 0.32079239188053 | 2.9621874916818 | 0.021505376344086 | FGFR2//FZD2//DLX5//INSIG2//BCR//PAX8//FZD3//KCNQ4// |
| GO:0003012 | muscle system process | Biological process | 20 | 456 | 372 | 17653 | 2.08132899452933 | 0.00170846301682944 | 0.44610693438463 | 2.76739441795433 | 0.0537634408602151 | CHRM1//GNAO1//KCNIP2//ASPH//TLN1//CACNG1//SSPN//DAPK3//MYOM3//EDN1//BMP10//PIK3C2A//P2RX2//EHD3//SCN3B//DDX39B//SCN1A//KIT//ABCC8//PTAFR// |
| GO:0007409 | axonogenesis | Biological process | 20 | 456 | 372 | 17653 | 2.08132899452933 | 0.00170846301682944 | 0.44610693438463 | 2.76739441795433 | 0.0537634408602151 | EFNA3//NCAM1//SPTBN2//CNTN2//VLDLR//UNC5A//FBXO45//TTL//RTN4//FZD3//NDN//PPP3CB//SEMA4G//NPTX1//DLX5//ZSWIM6//LRRC38//FGFR2//LRRN1//PARD6B// |
| GO:0048812 | neuron projection morphogenesis | Biological process | 25 | 628 | 372 | 17653 | 1.88910434216834 | 0.00184157156710238 | 0.44610693438463 | 2.73481139894119 | 0.0672043010752688 | LRRC38//FGFR2//RTN4//LRRN1//PARD6B//EFNA3//NCAM1//SPTBN2//CNTN2//VLDLR//UNC5A//FBXO45//TTL//FZD3//NDN//PPP3CB//SEMA4G//TLX2//NPTX1//NLGN1//ACTR2//LRP8//DLX5//ZSWIM6//DBNL// |
| GO:0000904 | cell morphogenesis involved in differentiation | Biological process | 27 | 701 | 372 | 17653 | 1.82776908563803 | 0.00196272909396739 | 0.44610693438463 | 2.70713963983669 | 0.0725806451612903 | LRRC38//FGFR2//RTN4//LRRN1//PARD6B//EFNA3//NCAM1//SPTBN2//CNTN2//VLDLR//UNC5A//FBXO45//TTL//PARVA//FERMT3//FZD3//NDN//PPP3CB//SEMA4G//TLX2//NPTX1//NLGN1//ACTR2//LRP8//PROX2//DLX5//ZSWIM6// |
| GO:0065008 | regulation of biological quality | Biological process | 103 | 3770 | 372 | 17653 | 1.2964968198283 | 0.0020543009238285 | 0.44610693438463 | 2.68733593854511 | 0.276881720430108 | ABCC8//JAM3//FOXP3//TP53INP2//P2RX2//CHRM1//DDAH1//EDN1//CYP4F2//AKAP10//VPS45//H3F3B//EHD3//NLGN1//TXNDC8//TMX1//KDELR1//SYPL2//LETM2//NDFIP1//ATP1B4//TLN1//CPLX1//SYT9//PPP3CB//BRK1//DAPK3//GAS2//RND1//KIT//PALM//RHOF//PARVA//VAV3//PTAFR//SCN3B//MEST//NFKB1//CTNS//PIK3C2A//CPLX3//SEPT7//RTN4//SCN1A//DPYS//GNA14//THBS1//KMT2E//TTL//ARHGAP40//CNTN2//ASPH//CD81//TRIM21//TNKS2//NPVF//RIT2//CA7//ARPC3//ACTR2//ARFIP2//VSNL1//CPS1//SLC26A1//GABRR1//HTR3E//SLC1A6//PRKAA1//CYP7A1//RAG1//TNFSF14//BCR//MAEA//CARHSP1//SERBP1//PSMC6//YWHAB//NUP214//FFAR3//SFRP1//GRIN2B//LARP1//SEMA4G//NDN//SLC9B1//TAF8//SPTBN2//LRRTM1//LRRN1//STEAP2//GRIA3//LRP8//SLC30A6//ANO3//HAAO//FERMT3//KCNIP2//GRP//ATP6V1G2//GRIPAP1//IARS//ZNF804A//PAX8// |
| GO:0023052 | signaling | Biological process | 169 | 6724 | 372 | 17653 | 1.19270923285551 | 0.00206771178119225 | 0.44610693438463 | 2.68450999776438 | 0.454301075268817 | CAMKK2//FGFR2//GRIN2B//IL5RA//KIT//NCAM1//MAPK4//PSMC6//SPTBN2//YWHAB//PRKAA1//THBS1//CD81//TSLP//CD209//NFKB1//UBE2D3//EDN1//EFNA3//FGF11//NDP//IL26//CCL16//C1QA//CXCL14//TRABD2B//MARK1//TNKS2//VPS26A//FZD2//GNAO1//PPP3CB//FZD3//RALBP1//PRDM4//PTPRT//AKAP10//CHRM1//TAGAP//GABRR1//HTR3E//GRP//KCNIP3//KCNIP2//ARHGAP40//IGFBP1//IL4R//LGALS3BP//NDFIP2//HIVEP3//BCR//RASL11B//SMPD1//ABCC8//VLDLR//LRP8//RERGL//NDFIP1//OGT//TNFSF14//SH3BP5//GNA14//SGK2//NUDT4//DAPK3//CARHSP1//RIT2//OXSR1//NLGN1//CLEC1A//TSPAN14//TSPAN18//PIK3C2A//VAV3//GNAZ//GPR34//FFAR3//MAS1//CYSLTR2//PTAFR//SFRP1//ARHGEF6//GPR3//PALM//GRIA3//NPVF//HEYL//ASCL1//EVC//ITGA5//TLN1//FERMT3//DBNL//DDAH1//ARFIP2//RND1//RHOF//RALGPS2//CPLX1//NOVA1//NPTX1//SLC1A6//BSN//P2RX2//SYT9//RGS5//ATP6V1G2//POLR2E//PTBP1//UBE4B//BMP10//AKAP7//TTK//PIP4K2C//PIK3IP1//CPLX3//MGRN1//BRK1//NREP//LRRTM1//NDN//JAM3//SCN1A//HLA-DPA1//FOXP3//DLX5//CTH//EDEM3//CREB3L3//LARP1//USP46//CA7//RABL3//RAB2A//INSIG2//UFM1//UBQLN1//WDFY1//C9ORF129//MTAP//VSNL1//EXTL2//GOSR2//UNC5A//ARPC3//ACTR2//PTPN18//PAX8//EEF1E1//ERH//TRIM67//CNTN2//PAX5//CGNL1//RTN4//TRIM21//CACNG1//CANX//SEMA4G//CDC5L//SCN3B//RBMS3//LYPD6//PATE1//BAG5//LDOC1//EHD3//ASPH// |
| GO:0050808 | synapse organization | Biological process | 15 | 304 | 372 | 17653 | 2.3414951188455 | 0.00208384648308894 | 0.44610693438463 | 2.68113427865617 | 0.0403225806451613 | NLGN1//DBNL//SPTBN2//BSN//P2RX2//LRRTM1//LRRN1//PALM//FBXO45//ACTR2//LRP8//CNTN2//GRIN2B//ZNF804A//GRIPAP1// |
| GO:0034976 | response to endoplasmic reticulum stress | Biological process | 14 | 274 | 372 | 17653 | 2.42467231771447 | 0.00210940670117079 | 0.44610693438463 | 2.67583967865506 | 0.0376344086021505 | UBE4B//UBQLN1//PSMC6//CTH//EDEM3//CREB3L3//EXTL2//TLN1//GOSR2//IGFBP1//DNAJB12//UFM1//THBS1//TMX1// |
| GO:0006811 | ion transport | Biological process | 51 | 1610 | 372 | 17653 | 1.50321077940293 | 0.00212968655216157 | 0.44610693438463 | 2.6716843114363 | 0.137096774193548 | SLC27A4//EDN1//P2RX2//KCNIP2//KCNA6//ABCC8//KCNQ4//SCN3B//SCN1A//SLC5A3//SLC23A2//SLC26A1//ANO8//BEST1//NDFIP2//NDFIP1//SLC39A2//SLC1A6//ATP1B4//THBS1//CPLX1//SLC9B1//STEAP2//CA7//CTNS//PCTP//CYP4F2//REP15//ATP6V1G2//SGK2//GABRR1//HTR3E//GRIA3//ASPH//ANO3//CACNG1//CHRM1//GNAO1//SLC30A6//LRRC38//KCNIP3//SLC35A2//GRIN2B//RALBP1//OXSR1//UBQLN1//EHD3//PTAFR//SESTD1//NLGN1//TOMM40L// |
| GO:0000902 | cell morphogenesis | Biological process | 35 | 997 | 372 | 17653 | 1.6658982323314 | 0.00217677492750152 | 0.44610693438463 | 2.66218647348411 | 0.0940860215053763 | LRRC38//FGFR2//RTN4//LRRN1//PARD6B//EFNA3//NCAM1//SPTBN2//CNTN2//VLDLR//UNC5A//DAPK3//GAS2//RND1//KIT//PALM//RHOF//PARVA//SEPT7//FBXO45//TTL//FERMT3//FZD3//NDN//PPP3CB//DBNL//SEMA4G//TLX2//NPTX1//NLGN1//ACTR2//LRP8//PROX2//DLX5//ZSWIM6// |
| GO:0021872 | forebrain generation of neurons | Biological process | 6 | 64 | 372 | 17653 | 4.44884072580645 | 0.00225325737891292 | 0.44819940157612 | 2.64718919804206 | 0.0161290322580645 | FGFR2//CNTN2//OTP//DLX5//ASCL1//SATB2// |
| GO:0120039 | plasma membrane bounded cell projection morphogenesis | Biological process | 25 | 642 | 372 | 17653 | 1.84790892037651 | 0.00247006964710824 | 0.477288029239801 | 2.60729080102035 | 0.0672043010752688 | LRRC38//FGFR2//RTN4//LRRN1//PARD6B//EFNA3//NCAM1//SPTBN2//CNTN2//VLDLR//UNC5A//FBXO45//TTL//FZD3//NDN//PPP3CB//DBNL//SEMA4G//TLX2//NPTX1//NLGN1//ACTR2//LRP8//DLX5//ZSWIM6// |
| GO:0048858 | cell projection morphogenesis | Biological process | 25 | 644 | 372 | 17653 | 1.8421700727977 | 0.00257328024267649 | 0.481905563114506 | 2.58951291444241 | 0.0672043010752688 | LRRC38//FGFR2//RTN4//LRRN1//PARD6B//EFNA3//NCAM1//SPTBN2//CNTN2//VLDLR//UNC5A//FBXO45//TTL//FZD3//NDN//PPP3CB//DBNL//SEMA4G//TLX2//NPTX1//NLGN1//ACTR2//LRP8//DLX5//ZSWIM6// |
| GO:0006513 | protein monoubiquitination | Biological process | 6 | 66 | 372 | 17653 | 4.31402737047898 | 0.00263647875724334 | 0.481905563114506 | 2.57897572353569 | 0.0161290322580645 | RAG1//UBE2E1//UBE4B//MGRN1//TRIM21//UBE2D3// |
| GO:0051128 | regulation of cellular component organization | Biological process | 70 | 2403 | 372 | 17653 | 1.3823558365663 | 0.00278855222896004 | 0.496288913801493 | 2.55462121685766 | 0.188172043010753 | SGK2//IGFBP7//LRRTM1//DAPK3//TTK//ZW10//BRK1//GAS2//RND1//KIT//PALM//RHOF//PARVA//PAN3//SFRP1//ZNF804A//TRIM67//RIT2//BAG5//SEPT7//EIF4G2//H3F3B//IGFBP1//BMP10//RTN4//TTL//ARHGAP40//PAXBP1//FOXP3//SKA3//MAPRE1//CNTN2//TNKS2//TRABD2B//ARPC3//ACTR2//ARFIP2//GPR3//EDN1//PSMC6//CEP85//SEMA4G//BCR//TLX2//NLGN1//PAX5//PRDM12//SPTBN2//THBS1//LRRN1//LRP8//DDX39B//OGT//JAM3//VLDLR//YWHAB//PEX5//IL4R//TNFSF14//ASPH//ZSWIM6//GRIN2B//EHD3//CAMKK2//PRKAA1//UBE2D3//ABCC8//GRIPAP1//KMT2E//PIP4K2C// |
| GO:0051179 | localization | Biological process | 163 | 6497 | 372 | 17653 | 1.19055734573939 | 0.00293963702636178 | 0.50976321049448 | 2.53170629107762 | 0.438172043010753 | ZW10//SPRN//NUP214//SLC27A4//SEMA4G//FBXO45//SATB2//MARK1//ASCL1//NDN//FZD3//EDN1//LRRTM1//JAM3//CCL16//KIT//LGALS3BP//THBS1//TLN1//FERMT3//BCR//SPTBN2//AKAP10//MAPRE1//AKAP7//CD81//VAV3//YKT6//SYT9//NLGN1//DDX39B//PAN3//YWHAB//ZDHHC20//GOLGA7B//KDELR1//GOSR2//TIMM23//TOMM34//SLC26A1//CTNS//SLC1A6//TOMM40L//P2RX2//KCNIP2//KCNA6//ABCC8//KCNQ4//SCN3B//SCN1A//SLC5A3//SLC23A2//ANO8//BEST1//NDFIP2//NDFIP1//SLC39A2//RALBP1//PCTP//ANO3//VLDLR//TMED2//VPS45//AP1S1//ARFIP2//RABL3//KCNIP3//RAB2A//MLPH//VPS26A//CPLX1//PIK3C2A//KIF2A//STEAP2//TGOLN2//SYS1//CD209//LRP8//CLEC9A//DBNL//TMPRSS2//ANP32E//VPS53//PPP3CB//MGRN1//SLC35A2//CANX//ATP1B4//BMP10//RTN4//TNFSF14//MEST//NFKB1//FFAR3//EHD3//ANKRD50//SLC9B1//CA7//CPLX3//ARPP19//ACTR2//AVL9//RND1//RHOF//PEX5//CNTN2//PTAFR//RIT2//PARD6B//ZNF268//DAPK3//ITGA5//SFRP1//NPVF//CYP4F2//SGK2//REP15//ATP6V1G2//GABRR1//HTR3E//GRIA3//ASPH//TTK//SEPT7//CACNG1//BSN//VSNL1//ARPC3//BRK1//CHRM1//IL4R//ALAD//MGST1//TSPAN14//WDR91//NUDT4//FOXP3//IL26//MOGAT2//TAF8//GNAO1//PSMC6//CXCL14//PRKAA1//SLC30A6//TULP4//TNKS2//PARVA//LRRC38//PALM//TTL//GRP//GRIN2B//GRIPAP1//OXSR1//UBQLN1//ZSWIM6//NUCB1//TP53INP2//UBE2D3//SESTD1//PAX8//TSLP// |
| GO:0048562 | embryonic organ morphogenesis | Biological process | 14 | 288 | 372 | 17653 | 2.30680630227001 | 0.00331332174227216 | 0.5476255814114 | 2.47973638962095 | 0.0376344086021505 | TMED2//MICAL2//FGFR2//DLX5//INSIG2//BCR//PAX8//FZD3//KCNQ4//EDN1//PAX5//SATB2//HOXC9//FZD2// |
| GO:0045893 | positive regulation of transcription, DNA-templated | Biological process | 46 | 1450 | 372 | 17653 | 1.50544679273267 | 0.00343707239837395 | 0.5476255814114 | 2.4638113198025 | 0.123655913978495 | HEYL//DDX39B//MICAL2//INSIG2//PSMC6//ZNF268//PRDM4//EDN1//ELK1//TET3//FGFR2//SATB2//ATP1B4//GABPA//PROX2//TLX2//MCIDAS//ASCL1//NDN//NFKB1//PAX5//FOXP3//PLAG1//PPP3CB//IL26//RIT2//PAX8//CREB3L3//OGT//PAXBP1//CD81//CDC5L//DLX5//PRKAA1//CAMKK2//TAF8//ZNF281//FZD2//BMP10//NDP//NFYA//KMT2E//TP53INP2//HIVEP3//GPBP1L1//SFRP1// |
| GO:1903508 | positive regulation of nucleic acid-templated transcription | Biological process | 46 | 1450 | 372 | 17653 | 1.50544679273267 | 0.00343707239837395 | 0.5476255814114 | 2.4638113198025 | 0.123655913978495 | HEYL//DDX39B//MICAL2//CAMKK2//TAF8//DLX5//ELK1//ZNF281//FZD2//BMP10//NDP//NFKB1//NFYA//FOXP3//PPP3CB//KMT2E//TP53INP2//HIVEP3//GPBP1L1//SFRP1//PAX8//INSIG2//PSMC6//ZNF268//PRDM4//EDN1//TET3//FGFR2//SATB2//ATP1B4//GABPA//PROX2//TLX2//MCIDAS//ASCL1//NDN//PAX5//PLAG1//IL26//RIT2//CREB3L3//OGT//PAXBP1//CD81//CDC5L//PRKAA1// |
| GO:1902680 | positive regulation of RNA biosynthetic process | Biological process | 46 | 1451 | 372 | 17653 | 1.5044092690988 | 0.00348187195041996 | 0.5476255814114 | 2.45818720457095 | 0.123655913978495 | HEYL//DDX39B//MICAL2//CAMKK2//TAF8//DLX5//ELK1//ZNF281//FZD2//BMP10//NDP//NFKB1//NFYA//FOXP3//PPP3CB//KMT2E//TP53INP2//HIVEP3//GPBP1L1//SFRP1//PAX8//INSIG2//PSMC6//ZNF268//PRDM4//EDN1//TET3//FGFR2//SATB2//ATP1B4//GABPA//PROX2//TLX2//MCIDAS//ASCL1//NDN//PAX5//PLAG1//IL26//RIT2//CREB3L3//OGT//PAXBP1//CD81//CDC5L//PRKAA1// |
| GO:0032990 | cell part morphogenesis | Biological process | 25 | 663 | 372 | 17653 | 1.78937786859988 | 0.0037506176863348 | 0.572459670646357 | 2.42589720275869 | 0.0672043010752688 | LRRC38//FGFR2//RTN4//LRRN1//PARD6B//EFNA3//NCAM1//SPTBN2//CNTN2//VLDLR//UNC5A//FBXO45//TTL//FZD3//NDN//PPP3CB//DBNL//SEMA4G//TLX2//NPTX1//NLGN1//ACTR2//LRP8//DLX5//ZSWIM6// |
| GO:0010557 | positive regulation of macromolecule biosynthetic process | Biological process | 52 | 1698 | 372 | 17653 | 1.45325303646288 | 0.00386757404745213 | 0.572459670646357 | 2.41256136267268 | 0.139784946236559 | HEYL//DDX39B//MICAL2//EDN1//LARP1//RBMS3//PTAFR//THBS1//CAMKK2//TAF8//DLX5//ELK1//ZNF281//FZD2//BMP10//NDP//NFKB1//NFYA//FOXP3//PPP3CB//KMT2E//TP53INP2//HIVEP3//GPBP1L1//SFRP1//PAX8//TNKS2//INSIG2//MAS1//PSMC6//ZNF268//PRDM4//TET3//FGFR2//SATB2//ATP1B4//GABPA//PROX2//TLX2//MCIDAS//ASCL1//NDN//PAX5//PLAG1//IL26//RIT2//CREB3L3//OGT//PAXBP1//CD81//CDC5L//PRKAA1// |
| GO:0050869 | negative regulation of B cell activation | Biological process | 4 | 31 | 372 | 17653 | 6.12313562261533 | 0.00389370765189005 | 0.572459670646357 | 2.40963665937409 | 0.010752688172043 | HMGB3//SFRP1//FOXP3//NDFIP1// |
| GO:0007567 | parturition | Biological process | 3 | 16 | 372 | 17653 | 8.8976814516129 | 0.00423890867471439 | 0.580440091702923 | 2.37274594000051 | 0.00806451612903226 | EDN1//LDOC1//PTAFR// |
| GO:0080182 | histone H3-K4 trimethylation | Biological process | 3 | 16 | 372 | 17653 | 8.8976814516129 | 0.00423890867471439 | 0.580440091702923 | 2.37274594000051 | 0.00806451612903226 | KMT2E//TET3//OGT// |
| GO:0048522 | positive regulation of cellular process | Biological process | 132 | 5142 | 372 | 17653 | 1.21819676039197 | 0.00429739530299338 | 0.580440091702923 | 2.36679469518451 | 0.354838709677419 | KIT//PRKAA1//THBS1//CD81//CAMKK2//CDK2AP1//TSLP//OTP//FZD3//FGFR2//HEYL//MAS1//DBNL//DDX39B//ZNF268//CHRM1//EDN1//BRK1//PURA//SFRP1//TTK//CASP7//VAV3//TP53INP2//TRIM21//P2RX2//PAN3//RTN4//MICAL2//NFKB1//TNFSF14//BMP10//CYSLTR2//CNTN2//TRIM67//RIT2//ZNF804A//ARPP19//LARP1//RBMS3//PTAFR//TAF8//DLX5//ELK1//ZNF281//FZD2//NDP//NFYA//FOXP3//PPP3CB//KMT2E//HIVEP3//GPBP1L1//PAX8//EIF4G2//H3F3B//IGFBP1//DAPK3//MAPRE1//ITGA5//SEMA4G//FERMT3//PAXBP1//HLA-DPA1//UBQLN1//NDFIP2//NDFIP1//OXSR1//TNKS2//NLGN1//CA7//TRABD2B//IL26//INSIG2//WDFY1//ARPC3//ACTR2//ARFIP2//SMPD1//VSNL1//CD209//IL4R//ARHGEF6//EEF1E1//CTH//ASCL1//DDAH1//RAG1//ERH//TSPAN14//VLDLR//ASPH//OGT//EVC//PSMC6//TTL//PRDM4//TET3//SATB2//ATP1B4//GABPA//PROX2//TLX2//MCIDAS//NDN//PAX5//PLAG1//CREB3L3//CDC5L//SYT9//CLEC9A//LRP8//BCR//PALM//PRDM12//LRRTM1//LRRN1//GRIN2B//CCL16//PIK3C2A//GRP//LSM11//YWHAB//SEPT7//EFNA3//LRRC38//ABCC8//UBE2D3//FAM168A//CXCL14//PIP4K2C//FFAR3// |
| GO:0015833 | peptide transport | Biological process | 58 | 1951 | 372 | 17653 | 1.41073780746571 | 0.00433869663872787 | 0.580440091702923 | 2.3626407146397 | 0.155913978494624 | SPRN//NUP214//DDX39B//NLGN1//PAN3//YWHAB//ZDHHC20//GOLGA7B//GOSR2//TIMM23//TOMM34//KDELR1//TMED2//VPS45//AP1S1//ARFIP2//RABL3//KCNIP3//RAB2A//MLPH//VPS26A//SYT9//PPP3CB//ABCC8//CANX//YKT6//EHD3//VPS53//ANKRD50//ZW10//PEX5//EDN1//CPLX1//CPLX3//TOMM40L//REP15//ATP6V1G2//VLDLR//VSNL1//SYS1//SFRP1//CD209//FOXP3//CLEC9A//IL26//NDFIP2//NDFIP1//BCR//IL4R//FFAR3//GRP//CHRM1//GRIPAP1//NUCB1//PRKAA1//UBE2D3//PAX8//TSLP// |
| GO:0035587 | purinergic receptor signaling pathway | Biological process | 4 | 32 | 372 | 17653 | 5.9317876344086 | 0.00437711735573696 | 0.580440091702923 | 2.35881180927774 | 0.010752688172043 | GPR34//PTAFR//P2RX2//CNTN2// |
| GO:0010628 | positive regulation of gene expression | Biological process | 56 | 1877 | 372 | 17653 | 1.41579161439268 | 0.0047123249586076 | 0.586108143302666 | 2.32676476854693 | 0.150537634408602 | HEYL//DDX39B//MICAL2//CNTN2//LARP1//RBMS3//PTAFR//THBS1//CAMKK2//TAF8//DLX5//ELK1//ZNF281//FZD2//BMP10//NDP//NFKB1//NFYA//FOXP3//PPP3CB//KMT2E//TP53INP2//HIVEP3//GPBP1L1//SFRP1//PAX8//INSIG2//H3F3B//POLR2E//PSMC6//ZNF268//PRDM4//EDN1//TET3//FGFR2//SATB2//ATP1B4//GABPA//PROX2//TLX2//MCIDAS//ASCL1//NDN//PAX5//PLAG1//IL26//RIT2//CREB3L3//OGT//PAXBP1//CD81//CDC5L//PRKAA1//TMED2//KIT//ZNF804A// |
| GO:0071702 | organic substance transport | Biological process | 75 | 2667 | 372 | 17653 | 1.33448540706605 | 0.00479237063681063 | 0.586108143302666 | 2.31944960144991 | 0.201612903225806 | SPRN//NUP214//SLC27A4//EDN1//DDX39B//NLGN1//PAN3//YWHAB//ZDHHC20//GOLGA7B//GOSR2//TIMM23//TOMM34//SLC1A6//PCTP//ANO3//VLDLR//KDELR1//TMED2//VPS45//AP1S1//ARFIP2//RABL3//KCNIP3//RAB2A//MLPH//VPS26A//SYT9//PPP3CB//ABCC8//SLC35A2//CANX//THBS1//CPLX1//FFAR3//YKT6//EHD3//VPS53//ANKRD50//ZW10//SLC26A1//BEST1//CA7//SLC5A3//CTNS//SLC23A2//ARPP19//PEX5//CPLX3//TOMM40L//CYP4F2//NFKB1//REP15//ATP6V1G2//VSNL1//SYS1//SFRP1//NUDT4//CD209//FOXP3//CLEC9A//IL26//NDFIP2//NDFIP1//BCR//IL4R//GRP//CHRM1//GRIPAP1//RALBP1//NUCB1//PRKAA1//UBE2D3//PAX8//TSLP// |
| GO:0043550 | regulation of lipid kinase activity | Biological process | 5 | 53 | 372 | 17653 | 4.47682085615744 | 0.0050670089571877 | 0.586108143302666 | 2.29524832798607 | 0.0134408602150538 | CD81//WDR91//VAV3//KIT//PIK3IP1// |
| GO:0006790 | sulfur compound metabolic process | Biological process | 16 | 367 | 372 | 17653 | 2.0688523629545 | 0.00507024732078963 | 0.586108143302666 | 2.29497085576834 | 0.043010752688172 | CTH//CTNS//MGST1//EEF1E1//ACBD6//ST3GAL3//MICAL2//MTAP//XYLT2//NDST2//NDST3//FAR2//SULT4A1//SLC26A1//CPS1//PAX8// |
| GO:0042886 | amide transport | Biological process | 58 | 1972 | 372 | 17653 | 1.39571473750791 | 0.00543860923758115 | 0.586108143302666 | 2.26451214399026 | 0.155913978494624 | SPRN//NUP214//DDX39B//NLGN1//PAN3//YWHAB//ZDHHC20//GOLGA7B//GOSR2//TIMM23//TOMM34//KDELR1//TMED2//VPS45//AP1S1//ARFIP2//RABL3//KCNIP3//RAB2A//MLPH//VPS26A//SYT9//PPP3CB//ABCC8//CANX//YKT6//EHD3//VPS53//ANKRD50//ZW10//PEX5//EDN1//CPLX1//CPLX3//TOMM40L//REP15//ATP6V1G2//VLDLR//VSNL1//SYS1//SFRP1//CD209//FOXP3//CLEC9A//IL26//NDFIP2//NDFIP1//BCR//IL4R//FFAR3//GRP//CHRM1//GRIPAP1//NUCB1//PRKAA1//UBE2D3//PAX8//TSLP// |
| GO:0031062 | positive regulation of histone methylation | Biological process | 4 | 34 | 372 | 17653 | 5.58285895003163 | 0.00546190160553651 | 0.586108143302666 | 2.26265612780622 | 0.010752688172043 | PRDM12//OGT//KMT2E//PAXBP1// |
| GO:1901654 | response to ketone | Biological process | 10 | 183 | 372 | 17653 | 2.59313120629884 | 0.00554035426476565 | 0.586108143302666 | 2.25646246445681 | 0.0268817204301075 | THBS1//EDN1//IGFBP7//PRKAA1//SFRP1//ELK1//CPS1//PTAFR//LARP1//CDC5L// |
| GO:0031328 | positive regulation of cellular biosynthetic process | Biological process | 54 | 1812 | 372 | 17653 | 1.41420102542192 | 0.00564480746964968 | 0.586108143302666 | 2.24835086619157 | 0.145161290322581 | HEYL//DDX39B//MICAL2//ARPP19//LARP1//RBMS3//PTAFR//THBS1//CAMKK2//TAF8//DLX5//ELK1//ZNF281//FZD2//BMP10//NDP//NFKB1//NFYA//FOXP3//PPP3CB//KMT2E//TP53INP2//HIVEP3//GPBP1L1//SFRP1//PAX8//TNKS2//INSIG2//EDN1//DDAH1//MAS1//PRKAA1//PSMC6//ZNF268//PRDM4//TET3//FGFR2//SATB2//ATP1B4//GABPA//PROX2//TLX2//MCIDAS//ASCL1//NDN//PAX5//PLAG1//IL26//RIT2//CREB3L3//OGT//PAXBP1//CD81//CDC5L// |
| GO:0006810 | transport | Biological process | 129 | 5048 | 372 | 17653 | 1.21267924697101 | 0.00575957139817976 | 0.586108143302666 | 2.23960983364816 | 0.346774193548387 | SPRN//NUP214//SLC27A4//EDN1//LRRTM1//LGALS3BP//THBS1//TLN1//FERMT3//SPTBN2//VAV3//YKT6//SYT9//NLGN1//DDX39B//PAN3//YWHAB//ZDHHC20//GOLGA7B//GOSR2//TIMM23//TOMM34//SLC26A1//CTNS//SLC1A6//TOMM40L//P2RX2//KCNIP2//KCNA6//ABCC8//KCNQ4//SCN3B//SCN1A//SLC5A3//SLC23A2//ANO8//BEST1//NDFIP2//NDFIP1//SLC39A2//RALBP1//PCTP//ANO3//VLDLR//KDELR1//TMED2//VPS45//AP1S1//ARFIP2//RABL3//KCNIP3//RAB2A//MLPH//VPS26A//CPLX1//PIK3C2A//ZW10//KIF2A//STEAP2//TGOLN2//SYS1//CD209//LRP8//CLEC9A//DBNL//TMPRSS2//ANP32E//VPS53//PPP3CB//MGRN1//SLC35A2//CANX//ATP1B4//FFAR3//EHD3//ANKRD50//SLC9B1//CA7//CPLX3//ARPP19//ACTR2//PEX5//PTAFR//RIT2//CNTN2//CD81//NPVF//CYP4F2//NFKB1//SGK2//REP15//ATP6V1G2//GABRR1//HTR3E//GRIA3//ASPH//CACNG1//VSNL1//ARPC3//BRK1//CHRM1//KIT//IL4R//ALAD//MGST1//RHOF//TSPAN14//BCR//WDR91//SFRP1//NUDT4//FOXP3//IL26//MOGAT2//GNAO1//PSMC6//PRKAA1//SLC30A6//LRRC38//GRP//GRIN2B//GRIPAP1//OXSR1//UBQLN1//NUCB1//UBE2D3//SESTD1//PAX8//TSLP// |
| GO:0051149 | positive regulation of muscle cell differentiation | Biological process | 7 | 102 | 372 | 17653 | 3.25666772085178 | 0.00581583941762223 | 0.586108143302666 | 2.23538759338068 | 0.0188172043010753 | EDN1//BMP10//DDX39B//IL4R//TNFSF14//CTH//KIT// |
| GO:0070633 | transepithelial transport | Biological process | 3 | 18 | 372 | 17653 | 7.90905017921147 | 0.00598719598778997 | 0.586108143302666 | 2.2227765252145 | 0.00806451612903226 | BEST1//EDN1//SLC23A2// |
| GO:0043255 | regulation of carbohydrate biosynthetic process | Biological process | 6 | 78 | 372 | 17653 | 3.65033085194375 | 0.00604191146722266 | 0.586108143302666 | 2.21882564277978 | 0.0161290322580645 | PPP1R3F//ARPP19//MAEA//MAS1//PTAFR//NFKB1// |
| GO:0002823 | negative regulation of adaptive immune response based on somatic recombination of immune receptors built from immunoglobulin superfamily domains | Biological process | 4 | 35 | 372 | 17653 | 5.42334869431644 | 0.00606595856042736 | 0.586108143302666 | 2.21710056089631 | 0.010752688172043 | PPP3CB//FOXP3//IL4R//NDFIP1// |
| GO:0009653 | anatomical structure morphogenesis | Biological process | 74 | 2656 | 372 | 17653 | 1.32214543658505 | 0.00639043371875765 | 0.586108143302666 | 2.19446966527338 | 0.198924731182796 | VAV3//FGFR2//ITGA5//RTN4//JAM3//HS6ST1//EDN1//PAX8//TXNDC8//TLX2//FZD3//TMED2//PROX2//PPP3CB//MICAL2//DLX5//NAB1//PARVA//THBS1//FZD2//HEYL//UBE4B//PAX5//LRRC38//LRRN1//PARD6B//EFNA3//NCAM1//SPTBN2//CNTN2//VLDLR//UNC5A//DAPK3//GAS2//RND1//KIT//PALM//RHOF//ZNF281//SFRP1//SEPT7//ABCC8//FBXO45//PLAG1//MYOM3//TTL//FERMT3//ASPH//INSIG2//BCR//KCNQ4//BMP10//DDAH1//CYSLTR2//C2ORF49//NDN//SATB2//HOXC9//DBNL//SEMA4G//PSMC6//CRISPLD1//NPTX1//NLGN1//ACTR2//LRP8//NDP//PIK3C2A//IL4R//TNFSF14//ZSWIM6//FOXG1//MAS1//FOXP3// |
| GO:0010043 | response to zinc ion | Biological process | 5 | 56 | 372 | 17653 | 4.23699116743472 | 0.00640840297382486 | 0.586108143302666 | 2.19325018671691 | 0.0134408602150538 | CPS1//ALAD//HAAO//SLC30A6//ABCC8// |
| GO:0033036 | macromolecule localization | Biological process | 81 | 2955 | 372 | 17653 | 1.30077779597184 | 0.00645063494968755 | 0.586108143302666 | 2.19039753473317 | 0.217741935483871 | SPRN//NUP214//SLC27A4//AKAP10//MAPRE1//AKAP7//CD81//DDX39B//NLGN1//PAN3//YWHAB//ZDHHC20//GOLGA7B//KDELR1//GOSR2//TIMM23//TOMM34//PCTP//ANO3//VLDLR//TMED2//VPS45//AP1S1//ARFIP2//RABL3//KCNIP3//RAB2A//MLPH//VPS26A//TLN1//SYT9//PPP3CB//ABCC8//CANX//THBS1//MEST//NFKB1//YKT6//EHD3//VPS53//ANKRD50//ZW10//PEX5//CPLX1//CPLX3//TOMM40L//CYP4F2//EDN1//RIT2//REP15//ATP6V1G2//TTK//SEPT7//LRRTM1//BSN//VSNL1//SYS1//SFRP1//NUDT4//FOXP3//CLEC9A//IL26//NDFIP2//NDFIP1//TAF8//TULP4//BCR//TNKS2//CNTN2//PALM//SCN3B//TSPAN14//IL4R//CHRM1//GRIPAP1//NUCB1//TP53INP2//PRKAA1//UBE2D3//RTN4//TSLP// |
| GO:1905114 | cell surface receptor signaling pathway involved in cell-cell signaling | Biological process | 23 | 620 | 372 | 17653 | 1.76040149150191 | 0.00645540691441089 | 0.586108143302666 | 2.19007637692763 | 0.0618279569892473 | TRABD2B//MARK1//NDP//PRKAA1//TNKS2//VPS26A//FZD2//GNAO1//PPP3CB//FZD3//FOXP3//FGFR2//SFRP1//PSMC6//P2RX2//GRIA3//GRIN2B//RBMS3//IGFBP1//DAPK3//DLX5//NFKB1//NLGN1// |
| GO:0032879 | regulation of localization | Biological process | 75 | 2700 | 372 | 17653 | 1.31817502986858 | 0.00648438096580608 | 0.586108143302666 | 2.18813147770893 | 0.201612903225806 | LRRTM1//BCR//EDN1//SYT9//PPP3CB//ABCC8//P2RX2//THBS1//BMP10//RTN4//SCN3B//TNFSF14//MEST//NFKB1//FFAR3//ARPP19//CPLX1//RIT2//RND1//KIF2A//RHOF//PARD6B//ZNF268//DAPK3//MAPRE1//ITGA5//KIT//SEMA4G//FERMT3//SFRP1//NPVF//CYP4F2//NDFIP2//NDFIP1//SGK2//KCNA6//SCN1A//CACNG1//KCNQ4//VSNL1//CHRM1//IL4R//PTAFR//NUP214//NUDT4//CPLX3//FOXP3//CLEC9A//IL26//BEST1//GNAO1//PRKAA1//SLC30A6//JAM3//PIK3C2A//TTL//GRP//NLGN1//YWHAB//OXSR1//UBQLN1//KCNIP3//KCNIP2//EHD3//ZSWIM6//NUCB1//LRRC38//TP53INP2//UBE2D3//SESTD1//CD81//CXCL14//PAX8//TSLP//CA7// |
| GO:0043269 | regulation of ion transport | Biological process | 23 | 622 | 372 | 17653 | 1.75474103654531 | 0.00670049020652914 | 0.586108143302666 | 2.17389342323906 | 0.0618279569892473 | P2RX2//THBS1//SCN3B//CYP4F2//EDN1//KCNA6//SCN1A//CACNG1//KCNQ4//CHRM1//BEST1//GNAO1//OXSR1//UBQLN1//KCNIP3//KCNIP2//EHD3//PTAFR//LRRC38//ABCC8//SESTD1//NLGN1//CA7// |
| GO:0034314 | Arp2/3 complex-mediated actin nucleation | Biological process | 4 | 36 | 372 | 17653 | 5.27270011947431 | 0.00671284559803687 | 0.586108143302666 | 2.17309334189474 | 0.010752688172043 | ARFIP2//BRK1//ARPC3//ACTR2// |
| GO:0035418 | protein localization to synapse | Biological process | 4 | 36 | 372 | 17653 | 5.27270011947431 | 0.00671284559803687 | 0.586108143302666 | 2.17309334189474 | 0.010752688172043 | GRIPAP1//NLGN1//LRRTM1//BSN// |
| GO:0097306 | cellular response to alcohol | Biological process | 5 | 57 | 372 | 17653 | 4.16265798905867 | 0.00690599846966981 | 0.586108143302666 | 2.16077352222375 | 0.0134408602150538 | PRKAA1//SFRP1//CYP7A1//LRP8//LARP1// |
| GO:0006939 | smooth muscle contraction | Biological process | 7 | 106 | 372 | 17653 | 3.1337745993102 | 0.00714920330324833 | 0.586108143302666 | 2.14574235264384 | 0.0188172043010753 | CHRM1//DAPK3//EDN1//PIK3C2A//P2RX2//KIT//PTAFR// |
| GO:0055085 | transmembrane transport | Biological process | 47 | 1550 | 372 | 17653 | 1.4389368713146 | 0.00717229890633413 | 0.586108143302666 | 2.14434161946311 | 0.126344086021505 | SLC1A6//RALBP1//ATP1B4//THBS1//SLC23A2//ARPP19//PEX5//TIMM23//TOMM40L//SGK2//SLC9B1//P2RX2//GABRR1//HTR3E//GRIA3//ASPH//ATP6V1G2//ANO8//ANO3//BEST1//KCNA6//SCN1A//CACNG1//KCNQ4//SCN3B//BCR//KCNIP2//SLC39A2//SLC30A6//LRRC38//KCNIP3//ABCC8//SLC35A2//GRIN2B//STEAP2//OXSR1//UBQLN1//EHD3//SLC26A1//PTAFR//SESTD1//NLGN1//PRKAA1//FFAR3//CTNS//PSMC6//SLC5A3// |
| GO:0097305 | response to alcohol | Biological process | 10 | 191 | 372 | 17653 | 2.48451838090413 | 0.0074197039074348 | 0.586108143302666 | 2.1296134254397 | 0.0268817204301075 | ACTR2//ALAD//FGFR2//GRIN2B//USP46//PRKAA1//SFRP1//CYP7A1//LRP8//LARP1// |
| GO:0099175 | regulation of postsynapse organization | Biological process | 5 | 58 | 372 | 17653 | 4.09088802373007 | 0.00742998671472659 | 0.586108143302666 | 2.12901196278399 | 0.0134408602150538 | NLGN1//ACTR2//LRP8//GRIN2B//ZNF804A// |
| GO:0034220 | ion transmembrane transport | Biological process | 36 | 1119 | 372 | 17653 | 1.52667992735449 | 0.00760305569734686 | 0.586108143302666 | 2.11901182801097 | 0.0967741935483871 | SLC1A6//ATP1B4//THBS1//SLC23A2//KCNA6//SCN1A//CACNG1//KCNQ4//SLC9B1//SCN3B//KCNIP2//SLC39A2//SLC30A6//LRRC38//KCNIP3//ABCC8//SLC35A2//GRIN2B//P2RX2//HTR3E//STEAP2//OXSR1//UBQLN1//EHD3//SLC26A1//GABRR1//BEST1//ATP6V1G2//PTAFR//SESTD1//NLGN1//SGK2//GRIA3//ASPH//ANO8//ANO3// |
| GO:0042147 | retrograde transport, endosome to Golgi | Biological process | 6 | 82 | 372 | 17653 | 3.47226593233674 | 0.00768211548686272 | 0.586108143302666 | 2.1145191682812 | 0.0161290322580645 | EHD3//YKT6//AP1S1//VPS53//VPS26A//GOSR2// |
| GO:0050810 | regulation of steroid biosynthetic process | Biological process | 6 | 82 | 372 | 17653 | 3.47226593233674 | 0.00768211548686272 | 0.586108143302666 | 2.1145191682812 | 0.0161290322580645 | INSIG2//NFKB1//NFYA//PRKAA1//CYP7A1//IGFBP7// |
| GO:0050794 | regulation of cellular process | Biological process | 256 | 11074 | 372 | 17653 | 1.0970111138946 | 0.00768368025816377 | 0.586108143302666 | 2.11443071577863 | 0.688172043010753 | ZNF268//EDN1//FGFR2//SATB2//ZNF281//GABPA//HEYL//PROX2//KCNIP3//ASCL1//NFKB1//PAX5//FOXP3//GATAD2B//UBE2D3//VLDLR//LRP8//BHLHE40//PAXBP1//CAMKK2//GRIN2B//IL5RA//KIT//NCAM1//MAPK4//PSMC6//SPTBN2//YWHAB//H3F3B//PRKAA1//THBS1//CD81//PTBP1//SGK2//IGFBP7//MAS1//CDK2AP1//TSLP//OTP//FZD3//LRRTM1//CD209//BCR//NDFIP1//LIN9//NUP214//TRABD2B//MARK1//NDP//TNKS2//VPS26A//FZD2//GNAO1//PPP3CB//RALBP1//PRDM4//PTPRT//AKAP10//CHRM1//TAGAP//FGF11//GABRR1//HTR3E//GRP//KCNIP2//ARHGAP40//IGFBP1//IL4R//LGALS3BP//NDFIP2//HIVEP3//RASL11B//SMPD1//ABCC8//RERGL//OGT//TNFSF14//SH3BP5//CXCL14//GNA14//NUDT4//DAPK3//CARHSP1//RIT2//OXSR1//PPP1R3F//TXNDC8//TMX1//MCIDAS//ZNF618//ZNF519//ZNF781//EWSR1//ATP1B4//MYCBP//HMGB3//NAB1//NFYA//PFDN1//ASF1B//GPBP1L1//SNAPC1//ZNF17//ZNF322//ZNF468//ZNF682//ELK1//FOXG1//HOXC9//PTAFR//PURA//CREBZF//PRDM12//CDC5L//EIF1B//EIF4A2//EIF4G2//CEP85//GAS2//KMT2E//ZW10//TTK//RND1//CLEC1A//TSPAN14//TSPAN18//PIK3C2A//VAV3//GNAZ//GPR34//FFAR3//CYSLTR2//CCL16//SFRP1//ARHGEF6//GPR3//PALM//GRIA3//NPVF//EVC//ITGA5//TLN1//FERMT3//DBNL//DDAH1//ARFIP2//RHOF//RALGPS2//MAEA//TTC28//SYT9//BRK1//DDX39B//UFM1//RGS5//LDOC1//ASPH//NDN//EEF1E1//ATP6V1G2//PARVA//POLR2E//UBE4B//CASP7//MAPRE1//BMP10//IL26//PIP4K2C//TP53INP2//TRIM21//P2RX2//PPP4R2//PAN3//RTN4//PAX8//CTNS//MICAL2//AKAP7//PPP1R26//CNTN2//ZNF804A//TRIM67//BAG5//PIK3IP1//ARPP19//LARP1//RBMS3//MGRN1//SERBP1//TNFAIP8L2//ANP32E//SEPT7//TAF8//DLX5//PASD1//NREP//MEX3B//CPLX1//HLA-DPA1//NLGN1//KIF2A//PARD6B//SEMA4G//TTL//CTH//EDEM3//CREB3L3//SKA3//UBQLN1//USP46//CA7//RABL3//RAB2A//INSIG2//WDFY1//ARPC3//ACTR2//C9ORF129//MTAP//VSNL1//EXTL2//GOSR2//UNC5A//PTPN18//RAG1//WDR91//UBXN2B//FAM57B//EFNA3//ERH//TET3//TLX2//PLAG1//CPLX3//CLEC9A//CGNL1//IFRD2//NOVA1//LRRN1//SCN3B//SLC30A6//CANX//CYP7A1//JAM3//BSN//LYPD6//PATE1//LSM11//SNRPA//PEX5//ALAD//ZSWIM6//EHD3//LRRC38//FAM168A//GRIPAP1// |
| GO:0009891 | positive regulation of biosynthetic process | Biological process | 54 | 1841 | 372 | 17653 | 1.3919240945489 | 0.00770603228117653 | 0.586108143302666 | 2.1131691760254 | 0.145161290322581 | HEYL//DDX39B//MICAL2//EDN1//ARPP19//LARP1//RBMS3//PTAFR//THBS1//CAMKK2//TAF8//DLX5//ELK1//ZNF281//FZD2//BMP10//NDP//NFKB1//NFYA//FOXP3//PPP3CB//KMT2E//TP53INP2//HIVEP3//GPBP1L1//SFRP1//PAX8//TNKS2//INSIG2//DDAH1//PRKAA1//MAS1//PSMC6//ZNF268//PRDM4//TET3//FGFR2//SATB2//ATP1B4//GABPA//PROX2//TLX2//MCIDAS//ASCL1//NDN//PAX5//PLAG1//IL26//RIT2//CREB3L3//OGT//PAXBP1//CD81//CDC5L// |
| GO:0007268 | chemical synaptic transmission | Biological process | 24 | 667 | 372 | 17653 | 1.70750108816559 | 0.00784070468041526 | 0.586108143302666 | 2.10564490350652 | 0.0645161290322581 | NLGN1//CPLX1//CHRM1//P2RX2//SYT9//CPLX3//USP46//CA7//GRIA3//EDN1//GRIN2B//PPP3CB//CNTN2//KIT//LRP8//LRRTM1//GABRR1//HTR3E//KCNIP2//NOVA1//NPTX1//RIT2//SLC1A6//BSN// |
| GO:0098916 | anterograde trans-synaptic signaling | Biological process | 24 | 667 | 372 | 17653 | 1.70750108816559 | 0.00784070468041526 | 0.586108143302666 | 2.10564490350652 | 0.0645161290322581 | NLGN1//CPLX1//GABRR1//HTR3E//GRIN2B//KCNIP2//NOVA1//NPTX1//RIT2//SLC1A6//BSN//CHRM1//P2RX2//SYT9//CPLX3//USP46//CA7//GRIA3//EDN1//PPP3CB//CNTN2//KIT//LRP8//LRRTM1// |
| GO:0019218 | regulation of steroid metabolic process | Biological process | 7 | 108 | 372 | 17653 | 3.07574173636002 | 0.00789501244499137 | 0.586108143302666 | 2.10264718107218 | 0.0188172043010753 | IGFBP7//INSIG2//NFKB1//NFYA//PRKAA1//CYP7A1//KIT// |
| GO:0031060 | regulation of histone methylation | Biological process | 5 | 59 | 372 | 17653 | 4.0215509385821 | 0.00798104986416788 | 0.586108143302666 | 2.09793997578904 | 0.0134408602150538 | PAXBP1//PAX5//PRDM12//OGT//KMT2E// |
| GO:0032409 | regulation of transporter activity | Biological process | 12 | 255 | 372 | 17653 | 2.23314358001265 | 0.00808757855609048 | 0.586108143302666 | 2.09218148792313 | 0.032258064516129 | NDFIP2//NDFIP1//SGK2//OXSR1//UBQLN1//EHD3//PTAFR//LRRC38//ABCC8//NLGN1//SCN3B//KCNIP2// |
| GO:0007413 | axonal fasciculation | Biological process | 3 | 20 | 372 | 17653 | 7.11814516129032 | 0.00810839920511165 | 0.586108143302666 | 2.09106487760246 | 0.00806451612903226 | NDN//RTN4//CNTN2// |
| GO:0106030 | neuron projection fasciculation | Biological process | 3 | 20 | 372 | 17653 | 7.11814516129032 | 0.00810839920511165 | 0.586108143302666 | 2.09106487760246 | 0.00806451612903226 | NDN//RTN4//CNTN2// |
| GO:1990126 | retrograde transport, endosome to plasma membrane | Biological process | 3 | 20 | 372 | 17653 | 7.11814516129032 | 0.00810839920511165 | 0.586108143302666 | 2.09106487760246 | 0.00806451612903226 | GRIPAP1//ANKRD50//VPS26A// |
| GO:0002820 | negative regulation of adaptive immune response | Biological process | 4 | 38 | 372 | 17653 | 4.9951895868704 | 0.00814001191384601 | 0.586108143302666 | 2.08937495947028 | 0.010752688172043 | PPP3CB//FOXP3//IL4R//NDFIP1// |
| GO:0007223 | Wnt signaling pathway, calcium modulating pathway | Biological process | 4 | 38 | 372 | 17653 | 4.9951895868704 | 0.00814001191384601 | 0.586108143302666 | 2.08937495947028 | 0.010752688172043 | FZD2//GNAO1//PPP3CB//FZD3// |
| GO:0007154 | cell communication | Biological process | 165 | 6744 | 372 | 17653 | 1.16102604943941 | 0.00825524490179824 | 0.586108143302666 | 2.08327003834038 | 0.443548387096774 | CAMKK2//FGFR2//GRIN2B//IL5RA//KIT//NCAM1//MAPK4//PSMC6//SPTBN2//YWHAB//PRKAA1//THBS1//CD81//TSLP//CD209//NFKB1//UBE2D3//EDN1//EFNA3//FGF11//NDP//IL26//CCL16//C1QA//CXCL14//TRABD2B//MARK1//TNKS2//VPS26A//FZD2//GNAO1//PPP3CB//FZD3//RALBP1//PRDM4//PTPRT//AKAP10//CHRM1//TAGAP//GABRR1//HTR3E//GRP//KCNIP3//KCNIP2//ARHGAP40//IGFBP1//IL4R//LGALS3BP//NDFIP2//HIVEP3//BCR//RASL11B//SMPD1//ABCC8//VLDLR//LRP8//RERGL//NDFIP1//OGT//TNFSF14//SH3BP5//GNA14//SGK2//NUDT4//DAPK3//CARHSP1//RIT2//OXSR1//NLGN1//CLEC1A//TSPAN14//TSPAN18//PIK3C2A//VAV3//GNAZ//GPR34//FFAR3//MAS1//CYSLTR2//PTAFR//SFRP1//ARHGEF6//GPR3//PALM//GRIA3//NPVF//HEYL//ASCL1//EVC//ITGA5//TLN1//FERMT3//DBNL//DDAH1//ARFIP2//RND1//RHOF//RALGPS2//CPLX1//NOVA1//NPTX1//SLC1A6//BSN//P2RX2//SYT9//RGS5//ATP6V1G2//POLR2E//PTBP1//UBE4B//BMP10//AKAP7//TTK//PIP4K2C//PIK3IP1//CPLX3//MGRN1//BRK1//NREP//LRRTM1//NDN//JAM3//SCN1A//HLA-DPA1//FOXP3//DLX5//CTH//EDEM3//CREB3L3//LARP1//USP46//CA7//RABL3//RAB2A//INSIG2//UFM1//UBQLN1//WDFY1//C9ORF129//MTAP//VSNL1//EXTL2//GOSR2//UNC5A//ARPC3//ACTR2//PTPN18//PAX8//EEF1E1//ERH//TRIM67//CNTN2//PAX5//CGNL1//RTN4//TRIM21//CANX//SEMA4G//CDC5L//SCN3B//RBMS3//LYPD6//PATE1//BAG5//LDOC1// |
| GO:0099537 | trans-synaptic signaling | Biological process | 24 | 670 | 372 | 17653 | 1.69985556090515 | 0.00826193686988785 | 0.586108143302666 | 2.08291812782519 | 0.0645161290322581 | NLGN1//CPLX1//GABRR1//HTR3E//GRIN2B//KCNIP2//NOVA1//NPTX1//RIT2//SLC1A6//BSN//CHRM1//P2RX2//SYT9//CPLX3//USP46//CA7//GRIA3//EDN1//PPP3CB//CNTN2//KIT//LRP8//LRRTM1// |
| GO:0051254 | positive regulation of RNA metabolic process | Biological process | 46 | 1523 | 372 | 17653 | 1.43328814803832 | 0.00834859885631083 | 0.586108143302666 | 2.07838640595744 | 0.123655913978495 | HEYL//DDX39B//MICAL2//CAMKK2//TAF8//DLX5//ELK1//ZNF281//FZD2//BMP10//NDP//NFKB1//NFYA//FOXP3//PPP3CB//KMT2E//TP53INP2//HIVEP3//GPBP1L1//SFRP1//PAX8//INSIG2//PSMC6//ZNF268//PRDM4//EDN1//TET3//FGFR2//SATB2//ATP1B4//GABPA//PROX2//TLX2//MCIDAS//ASCL1//NDN//PAX5//PLAG1//IL26//RIT2//CREB3L3//OGT//PAXBP1//CD81//CDC5L//PRKAA1// |
| GO:0071705 | nitrogen compound transport | Biological process | 64 | 2264 | 372 | 17653 | 1.34146434135036 | 0.00836937973551068 | 0.586108143302666 | 2.07730672688448 | 0.172043010752688 | SPRN//NUP214//EDN1//DDX39B//NLGN1//PAN3//YWHAB//ZDHHC20//GOLGA7B//GOSR2//TIMM23//TOMM34//SLC1A6//KDELR1//TMED2//VPS45//AP1S1//ARFIP2//RABL3//KCNIP3//RAB2A//MLPH//VPS26A//SYT9//PPP3CB//ABCC8//CANX//CPLX1//FFAR3//YKT6//EHD3//VPS53//ANKRD50//ZW10//CTNS//SLC23A2//PEX5//CPLX3//TOMM40L//REP15//ATP6V1G2//VLDLR//VSNL1//SYS1//SFRP1//NUDT4//CD209//FOXP3//CLEC9A//IL26//NDFIP2//NDFIP1//BCR//SLC35A2//IL4R//GRP//CHRM1//GRIPAP1//RALBP1//NUCB1//PRKAA1//UBE2D3//PAX8//TSLP// |
| GO:0099536 | synaptic signaling | Biological process | 24 | 671 | 372 | 17653 | 1.69732224412288 | 0.00840640099073763 | 0.586108143302666 | 2.07538989771018 | 0.0645161290322581 | NLGN1//CPLX1//GABRR1//HTR3E//GRIN2B//KCNIP2//NOVA1//NPTX1//RIT2//SLC1A6//BSN//CHRM1//P2RX2//SYT9//CPLX3//USP46//CA7//GRIA3//EDN1//PPP3CB//CNTN2//KIT//LRP8//LRRTM1// |
| GO:0030166 | proteoglycan biosynthetic process | Biological process | 5 | 60 | 372 | 17653 | 3.95452508960573 | 0.00855985950758976 | 0.588704282059488 | 2.06753336331107 | 0.0134408602150538 | EXTL2//XYLT2//NDST2//NDST3//HS6ST1// |
| GO:0051234 | establishment of localization | Biological process | 130 | 5155 | 372 | 17653 | 1.19671370315906 | 0.00883256494543212 | 0.588704282059488 | 2.0539131605346 | 0.349462365591398 | ZW10//SPRN//NUP214//SLC27A4//EDN1//LRRTM1//LGALS3BP//THBS1//TLN1//FERMT3//SPTBN2//VAV3//YKT6//SYT9//NLGN1//DDX39B//PAN3//YWHAB//ZDHHC20//GOLGA7B//GOSR2//TIMM23//TOMM34//SLC26A1//CTNS//SLC1A6//TOMM40L//P2RX2//KCNIP2//KCNA6//ABCC8//KCNQ4//SCN3B//SCN1A//SLC5A3//SLC23A2//ANO8//BEST1//NDFIP2//NDFIP1//SLC39A2//RALBP1//PCTP//ANO3//VLDLR//KDELR1//TMED2//VPS45//AP1S1//ARFIP2//RABL3//KCNIP3//RAB2A//MLPH//VPS26A//CPLX1//PIK3C2A//KIF2A//STEAP2//TGOLN2//SYS1//CD209//LRP8//CLEC9A//DBNL//TMPRSS2//ANP32E//VPS53//PPP3CB//MGRN1//SLC35A2//CANX//ATP1B4//FFAR3//EHD3//ANKRD50//SLC9B1//CA7//CPLX3//ARPP19//ACTR2//PEX5//PTAFR//RIT2//CNTN2//CD81//NPVF//CYP4F2//NFKB1//SGK2//REP15//ATP6V1G2//GABRR1//HTR3E//GRIA3//ASPH//CACNG1//VSNL1//ARPC3//BRK1//CHRM1//KIT//IL4R//ALAD//MGST1//RHOF//TSPAN14//BCR//WDR91//SFRP1//NUDT4//FOXP3//IL26//MOGAT2//GNAO1//PSMC6//PRKAA1//SLC30A6//LRRC38//TTL//GRP//GRIN2B//GRIPAP1//OXSR1//UBQLN1//NUCB1//UBE2D3//SESTD1//PAX8//TSLP// |
| GO:0010243 | response to organonitrogen compound | Biological process | 31 | 937 | 372 | 17653 | 1.56999288509427 | 0.0088603311988854 | 0.588704282059488 | 2.05255004389625 | 0.0833333333333333 | IGFBP1//PIK3C2A//ATP6V1G2//ALAD//CPS1//UBE4B//UBQLN1//PSMC6//PRKAA1//INSIG2//ABCC8//OGT//P2RX2//ACTR2//DNAJB12//MAS1//SMPD1//EDN1//GNAO1//ASCL1//PTAFR//CD81//SFRP1//CASP7//LARP1//GABPA//EDEM3//NFKB1//CDC5L//FFAR3//MGST1// |
| GO:0043029 | T cell homeostasis | Biological process | 4 | 39 | 372 | 17653 | 4.86710780259167 | 0.00892262966554692 | 0.588704282059488 | 2.04950713206691 | 0.010752688172043 | FOXP3//PPP3CB//RAG1//TNFSF14// |
| GO:0051147 | regulation of muscle cell differentiation | Biological process | 9 | 167 | 372 | 17653 | 2.55741742321808 | 0.00905066928960974 | 0.588704282059488 | 2.04331930388467 | 0.0241935483870968 | PTBP1//FGFR2//EDN1//BMP10//DDX39B//IL4R//TNFSF14//CTH//KIT// |
| GO:0051051 | negative regulation of transport | Biological process | 18 | 460 | 372 | 17653 | 1.85690743338008 | 0.00908129630332481 | 0.588704282059488 | 2.04185215399015 | 0.0483870967741935 | LRRTM1//THBS1//NPVF//CYP4F2//NFKB1//NDFIP2//NDFIP1//BCR//SFRP1//ABCC8//VSNL1//EDN1//FOXP3//GNAO1//OXSR1//UBQLN1//SESTD1//PRKAA1// |
| GO:0014821 | phasic smooth muscle contraction | Biological process | 3 | 21 | 372 | 17653 | 6.77918586789554 | 0.00931411476864782 | 0.588704282059488 | 2.03085841498785 | 0.00806451612903226 | EDN1//P2RX2//KIT// |
| GO:0014829 | vascular smooth muscle contraction | Biological process | 3 | 21 | 372 | 17653 | 6.77918586789554 | 0.00931411476864782 | 0.588704282059488 | 2.03085841498785 | 0.00806451612903226 | CHRM1//EDN1//PIK3C2A// |
| GO:0032674 | regulation of interleukin-5 production | Biological process | 3 | 21 | 372 | 17653 | 6.77918586789554 | 0.00931411476864782 | 0.588704282059488 | 2.03085841498785 | 0.00806451612903226 | FOXP3//TSLP//IL5RA// |
| GO:1902932 | positive regulation of alcohol biosynthetic process | Biological process | 3 | 21 | 372 | 17653 | 6.77918586789554 | 0.00931411476864782 | 0.588704282059488 | 2.03085841498785 | 0.00806451612903226 | PRKAA1//MAS1//PTAFR// |
| GO:0007188 | adenylate cyclase-modulating G-protein coupled receptor signaling pathway | Biological process | 10 | 199 | 372 | 17653 | 2.38463824498838 | 0.00975768774224435 | 0.611030020377764 | 2.01065308394768 | 0.0268817204301075 | GPR3//RIT2//EDN1//GNAZ//FFAR3//PALM//CHRM1//GNAO1//CYSLTR2//GNA14// |
| GO:0051130 | positive regulation of cellular component organization | Biological process | 38 | 1221 | 372 | 17653 | 1.47687423493875 | 0.0102910990383344 | 0.629959129367184 | 1.98753824226418 | 0.102150537634409 | BRK1//PAN3//TRIM67//RIT2//ZNF804A//PAXBP1//MAPRE1//KIT//TNKS2//TRABD2B//ARPC3//ACTR2//ARFIP2//FOXP3//EDN1//PSMC6//RTN4//BCR//NLGN1//PALM//SFRP1//PRDM12//LRRTM1//LRRN1//BMP10//LRP8//OGT//VLDLR//YWHAB//IL4R//TNFSF14//SEPT7//CAMKK2//PRKAA1//UBE2D3//ABCC8//KMT2E//PIP4K2C// |
| GO:0030032 | lamellipodium assembly | Biological process | 5 | 63 | 372 | 17653 | 3.76621437105308 | 0.0104693098978836 | 0.629959129367184 | 1.98008194462667 | 0.0134408602150538 | BRK1//VAV3//ARFIP2//KIT//ARHGEF6// |
| GO:0046888 | negative regulation of hormone secretion | Biological process | 5 | 63 | 372 | 17653 | 3.76621437105308 | 0.0104693098978836 | 0.629959129367184 | 1.98008194462667 | 0.0134408602150538 | NPVF//SFRP1//ABCC8//VSNL1//EDN1// |
| GO:0008104 | protein localization | Biological process | 72 | 2628 | 372 | 17653 | 1.3001178376786 | 0.0105489183769395 | 0.629959129367184 | 1.97679206804479 | 0.193548387096774 | SPRN//NUP214//DDX39B//NLGN1//PAN3//YWHAB//ZDHHC20//GOLGA7B//KDELR1//GOSR2//TIMM23//TOMM34//TMED2//VPS45//AP1S1//ARFIP2//RABL3//KCNIP3//RAB2A//MLPH//VPS26A//TLN1//SYT9//PPP3CB//ABCC8//CANX//YKT6//EHD3//VPS53//ANKRD50//ZW10//PEX5//CPLX1//CPLX3//TOMM40L//RIT2//REP15//ATP6V1G2//VLDLR//TTK//SEPT7//MAPRE1//LRRTM1//BSN//VSNL1//SYS1//SFRP1//FOXP3//CLEC9A//IL26//NDFIP2//NDFIP1//TAF8//CD81//TULP4//BCR//TNKS2//CNTN2//PALM//SCN3B//TSPAN14//IL4R//CHRM1//GRIPAP1//NUCB1//TP53INP2//PRKAA1//UBE2D3//RTN4//TSLP//AKAP10//AKAP7// |
| GO:0032634 | interleukin-5 production | Biological process | 3 | 22 | 372 | 17653 | 6.47104105571847 | 0.0106188586053318 | 0.629959129367184 | 1.97392216197828 | 0.00806451612903226 | IL5RA//FOXP3//TSLP// |
| GO:0035590 | purinergic nucleotide receptor signaling pathway | Biological process | 3 | 22 | 372 | 17653 | 6.47104105571847 | 0.0106188586053318 | 0.629959129367184 | 1.97392216197828 | 0.00806451612903226 | GPR34//PTAFR//P2RX2// |
| GO:0016055 | Wnt signaling pathway | Biological process | 19 | 504 | 372 | 17653 | 1.78895182625021 | 0.010833960519617 | 0.637131086905824 | 1.96521275137238 | 0.0510752688172043 | FZD2//GNAO1//PPP3CB//FZD3//FOXP3//FGFR2//SFRP1//TRABD2B//NDP//PSMC6//RBMS3//IGFBP1//DAPK3//DLX5//NFKB1//TNKS2//MARK1//PRKAA1//VPS26A// |
| GO:1902930 | regulation of alcohol biosynthetic process | Biological process | 5 | 64 | 372 | 17653 | 3.70736727150538 | 0.0111655855350845 | 0.648301269139309 | 1.95211849713457 | 0.0134408602150538 | NFKB1//NFYA//PRKAA1//MAS1//PTAFR// |
| GO:0198738 | cell-cell signaling by wnt | Biological process | 19 | 506 | 372 | 17653 | 1.78188087041523 | 0.0112624275150781 | 0.648301269139309 | 1.9483679911208 | 0.0510752688172043 | TRABD2B//MARK1//NDP//PRKAA1//TNKS2//VPS26A//FZD2//GNAO1//PPP3CB//FZD3//FOXP3//FGFR2//SFRP1//PSMC6//RBMS3//IGFBP1//DAPK3//DLX5//NFKB1// |
| GO:0048468 | cell development | Biological process | 58 | 2045 | 372 | 17653 | 1.34589215763599 | 0.0113114815552918 | 0.648301269139309 | 1.94648050832403 | 0.155913978494624 | TXNDC8//SEMA4G//OTP//FZD3//SATB2//ASCL1//KIT//JAM3//LRRC38//FGFR2//RTN4//LRRN1//PARD6B//EFNA3//NCAM1//SPTBN2//CNTN2//VLDLR//UNC5A//NDN//SFRP1//ZNF804A//TRIM67//RIT2//BAG5//HEYL//EDN1//RND1//FBXO45//MYOM3//TTL//NREP//UBE4B//NLGN1//GNAO1//TBC1D24//PRDM12//PARVA//FERMT3//MAEA//KCNIP2//BMP10//HS6ST1//PPP3CB//P2RX2//DBNL//MSI2//TLX2//FZD2//PLAG1//ABCC8//NPTX1//ACTR2//LRP8//DDX39B//PROX2//DLX5//ZSWIM6// |
| GO:0006109 | regulation of carbohydrate metabolic process | Biological process | 9 | 174 | 372 | 17653 | 2.45453281423804 | 0.01164276288723 | 0.661680717700307 | 1.93394394715221 | 0.0241935483870968 | PPP1R3F//NUP214//OGT//ARPP19//MAEA//PRKAA1//MAS1//PTAFR//NFKB1// |
| GO:0006940 | regulation of smooth muscle contraction | Biological process | 5 | 65 | 372 | 17653 | 3.65033085194375 | 0.0118927830046468 | 0.664718111243193 | 1.92471650516818 | 0.0134408602150538 | CHRM1//EDN1//KIT//PTAFR//DAPK3// |
| GO:0007585 | respiratory gaseous exchange | Biological process | 5 | 65 | 372 | 17653 | 3.65033085194375 | 0.0118927830046468 | 0.664718111243193 | 1.92471650516818 | 0.0134408602150538 | NLGN1//NDN//SLC5A3//EDN1//NDUFA12// |
| GO:2000191 | regulation of fatty acid transport | Biological process | 3 | 23 | 372 | 17653 | 6.18969144460028 | 0.0120240390783465 | 0.666545707269323 | 1.91994962095386 | 0.00806451612903226 | THBS1//CYP4F2//EDN1// |
| GO:0048518 | positive regulation of biological process | Biological process | 144 | 5841 | 372 | 17653 | 1.16990572758752 | 0.0121876245256873 | 0.668605436429652 | 1.91408093391556 | 0.387096774193548 | KIT//PRKAA1//THBS1//CD81//CAMKK2//CDK2AP1//SFRP1//TSLP//OTP//FZD3//FGFR2//CD209//NFKB1//PSMC6//IL4R//FOXP3//CREB3L3//FFAR3//UBE2D3//EDN1//C1QA//HEYL//MAS1//DBNL//DDX39B//ZNF268//CHRM1//BRK1//PURA//TTK//CASP7//PYURF//VAV3//SCN3B//TP53INP2//TRIM21//P2RX2//PAN3//BMP10//TMED2//GABPA//RBMS3//ZNF804A//RTN4//MICAL2//ITGA5//TNFSF14//NLGN1//CYSLTR2//CNTN2//TRIM67//RIT2//ARPP19//LARP1//PTAFR//TAF8//DLX5//ELK1//ZNF281//FZD2//NDP//NFYA//PPP3CB//KMT2E//HIVEP3//GPBP1L1//PAX8//HLA-DPA1//EIF4G2//H3F3B//IGFBP1//DAPK3//MAPRE1//SEMA4G//FERMT3//PAXBP1//UBQLN1//NDFIP2//NDFIP1//OXSR1//TNKS2//CA7//CYP4F2//SGK2//TRABD2B//POLR2E//RNF135//ABCC8//LRP8//IL26//INSIG2//WDFY1//ARPC3//ACTR2//ARFIP2//SMPD1//VSNL1//ARHGEF6//EEF1E1//CTH//ASCL1//PPIH//DDAH1//RAG1//ERH//TSPAN14//VLDLR//ASPH//OGT//EVC//TTL//PRDM4//TET3//SATB2//ATP1B4//PROX2//TLX2//MCIDAS//NDN//PAX5//PLAG1//CDC5L//SYT9//TMPRSS2//CLEC9A//CCL16//BCR//PALM//PRDM12//LRRTM1//LRRN1//GRIN2B//PIK3C2A//GRP//LSM11//YWHAB//EHD3//ZNF322//SEPT7//EFNA3//LRRC38//FAM168A//CXCL14//PIP4K2C// |
| GO:0007610 | behavior | Biological process | 21 | 583 | 372 | 17653 | 1.70933159962375 | 0.0123005195319921 | 0.668605436429652 | 1.91007654505553 | 0.0564516129032258 | USP46//GRIN2B//PPP3CB//CNTN2//ITGA5//ABCC8//VLDLR//CTNS//GNAO1//LRRTM1//NOVA1//SCN1A//PAX5//SPTBN2//ACTR2//KIT//RAG1//NLGN1//GRP//P2RX2//THBS1// |
| GO:0007165 | signal transduction | Biological process | 152 | 6210 | 372 | 17653 | 1.16152234515956 | 0.0123577819833959 | 0.668605436429652 | 1.9080594708987 | 0.408602150537634 | CAMKK2//FGFR2//GRIN2B//IL5RA//KIT//NCAM1//MAPK4//PSMC6//SPTBN2//YWHAB//PRKAA1//THBS1//CD81//TSLP//CD209//NFKB1//UBE2D3//TRABD2B//MARK1//NDP//TNKS2//VPS26A//FZD2//GNAO1//PPP3CB//FZD3//PTPRT//SGK2//NUDT4//DAPK3//CARHSP1//RIT2//BCR//SH3BP5//OXSR1//EDN1//CLEC1A//TSPAN14//TSPAN18//PIK3C2A//VAV3//CHRM1//GNAZ//GPR34//FFAR3//GRP//MAS1//CYSLTR2//PTAFR//CCL16//SFRP1//ARHGEF6//GNA14//GPR3//PALM//GABRR1//GRIA3//NPVF//HEYL//ASCL1//EVC//ITGA5//TLN1//FERMT3//DBNL//DDAH1//RALBP1//ARFIP2//RND1//RHOF//RALGPS2//RGS5//IGFBP1//ATP6V1G2//POLR2E//PTBP1//UBE4B//FGF11//BMP10//IL26//TNFSF14//CXCL14//AKAP7//TTK//PIP4K2C//PIK3IP1//MGRN1//BRK1//NREP//LRRTM1//IL4R//NDN//LRP8//HLA-DPA1//FOXP3//SMPD1//NLGN1//DLX5//RASL11B//CTH//EDEM3//CREB3L3//LARP1//RABL3//RAB2A//INSIG2//UFM1//UBQLN1//WDFY1//OGT//C9ORF129//P2RX2//MTAP//EXTL2//GOSR2//UNC5A//VLDLR//ARPC3//ACTR2//PTPN18//PAX8//NDFIP2//NDFIP1//EEF1E1//ERH//TRIM67//EFNA3//PAX5//TAGAP//ARHGAP40//CGNL1//RTN4//CNTN2//TRIM21//CANX//SEMA4G//CDC5L//RBMS3//BSN//LYPD6//PATE1//BAG5//LDOC1//PRDM4//AKAP10//HTR3E//KCNIP3//KCNIP2//LGALS3BP//HIVEP3//ABCC8//RERGL// |
| GO:0097327 | response to antineoplastic agent | Biological process | 6 | 91 | 372 | 17653 | 3.12885501595179 | 0.0125062877314033 | 0.67127003117048 | 1.9028715837536 | 0.0161290322580645 | CPS1//EDN1//PTAFR//NUCB1//LARP1//PRKAA1// |
| GO:1902117 | positive regulation of organelle assembly | Biological process | 5 | 66 | 372 | 17653 | 3.59502280873249 | 0.0126514964675488 | 0.673717091417579 | 1.897858101432 | 0.0134408602150538 | PAN3//EDN1//BMP10//SEPT7//PIP4K2C// |
| GO:0043583 | ear development | Biological process | 10 | 208 | 372 | 17653 | 2.28145678246485 | 0.013018272798934 | 0.687832647962427 | 1.88544663202364 | 0.0268817204301075 | FGFR2//DLX5//INSIG2//BCR//PAX8//FZD3//KCNQ4//EDN1//CXCL14//FZD2// |
| GO:0022603 | regulation of anatomical structure morphogenesis | Biological process | 35 | 1123 | 372 | 17653 | 1.47898534072521 | 0.0132920546178389 | 0.696853995197244 | 1.87640788284794 | 0.0940860215053763 | DAPK3//GAS2//RND1//KIT//PALM//RHOF//PARVA//SEPT7//EFNA3//ABCC8//THBS1//TTL//RTN4//CNTN2//EDN1//SFRP1//DDAH1//CYSLTR2//SEMA4G//TLX2//FZD2//PSMC6//FZD3//BMP10//BCR//FGFR2//NLGN1//ACTR2//LRP8//PAX8//PIK3C2A//IL4R//TNFSF14//ZSWIM6//ITGA5// |
| GO:0035588 | G-protein coupled purinergic receptor signaling pathway | Biological process | 3 | 24 | 372 | 17653 | 5.9317876344086 | 0.0135308344847919 | 0.703915643235751 | 1.86867541840869 | 0.00806451612903226 | GPR34//PTAFR//CNTN2// |
| GO:0045913 | positive regulation of carbohydrate metabolic process | Biological process | 5 | 68 | 372 | 17653 | 3.48928684376977 | 0.0142657727608989 | 0.715925035608703 | 1.84570469812507 | 0.0134408602150538 | ARPP19//PRKAA1//MAS1//PTAFR//NFKB1// |
| GO:0030072 | peptide hormone secretion | Biological process | 11 | 243 | 372 | 17653 | 2.14813708571176 | 0.0143252862198168 | 0.715925035608703 | 1.843896692052 | 0.0295698924731183 | SYT9//PPP3CB//ABCC8//CPLX1//CPLX3//VSNL1//SFRP1//FFAR3//GRP//PAX8//EDN1// |
| GO:0051049 | regulation of transport | Biological process | 52 | 1821 | 372 | 17653 | 1.35509261719603 | 0.0144701686852756 | 0.715925035608703 | 1.83952640608501 | 0.139784946236559 | LRRTM1//EDN1//SYT9//PPP3CB//ABCC8//P2RX2//THBS1//SCN3B//FFAR3//ARPP19//CPLX1//RIT2//NPVF//CYP4F2//NFKB1//NDFIP2//NDFIP1//SGK2//KCNA6//SCN1A//CACNG1//KCNQ4//VSNL1//CHRM1//IL4R//BCR//PTAFR//SFRP1//NUP214//NUDT4//CPLX3//FOXP3//CLEC9A//IL26//BEST1//GNAO1//PRKAA1//GRP//NLGN1//YWHAB//OXSR1//UBQLN1//KCNIP3//KCNIP2//EHD3//NUCB1//LRRC38//UBE2D3//SESTD1//PAX8//TSLP//CA7// |
| GO:0002704 | negative regulation of leukocyte mediated immunity | Biological process | 4 | 45 | 372 | 17653 | 4.21816009557945 | 0.0146510444944113 | 0.715925035608703 | 1.83413141271675 | 0.010752688172043 | PPP3CB//FOXP3//BCR//NDFIP1// |
| GO:0043551 | regulation of phosphatidylinositol 3-kinase activity | Biological process | 4 | 45 | 372 | 17653 | 4.21816009557945 | 0.0146510444944113 | 0.715925035608703 | 1.83413141271675 | 0.010752688172043 | VAV3//KIT//PIK3IP1//WDR91// |
| GO:0055023 | positive regulation of cardiac muscle tissue growth | Biological process | 4 | 45 | 372 | 17653 | 4.21816009557945 | 0.0146510444944113 | 0.715925035608703 | 1.83413141271675 | 0.010752688172043 | FGFR2//BMP10//EDN1//DDX39B// |
| GO:0006935 | chemotaxis | Biological process | 21 | 594 | 372 | 17653 | 1.67767731074183 | 0.0148842037074899 | 0.715925035608703 | 1.82727439478908 | 0.0564516129032258 | CCL16//KIT//EFNA3//NCAM1//SPTBN2//CNTN2//VLDLR//UNC5A//THBS1//TNFSF14//VAV3//FZD3//SEMA4G//CXCL14//PARVA//JAM3//EDN1//DLX5//ZSWIM6//RALBP1//PTAFR// |
| GO:0051960 | regulation of nervous system development | Biological process | 27 | 820 | 372 | 17653 | 1.56251966955153 | 0.0149153282326639 | 0.715925035608703 | 1.82636718493912 | 0.0725806451612903 | OTP//FZD3//SFRP1//ZNF804A//TRIM67//RIT2//BAG5//TTL//RTN4//CNTN2//EFNA3//NLGN1//NREP//ASCL1//HEYL//KIT//SEMA4G//TLX2//LRRTM1//LRRN1//PLAG1//ABCC8//ACTR2//LRP8//FZD2//VLDLR//ZSWIM6// |
| GO:0009987 | cellular process | Biological process | 352 | 16158 | 372 | 17653 | 1.03378598703395 | 0.0149633359905718 | 0.715925035608703 | 1.82497157219456 | 0.946236559139785 | CYP4F2//UBXN2B//UBQLN1//TP53INP2//CPS1//DDAH1//POLI//SKA3//CEP85//ZW10//ARPP19//MAPRE1//SEPT7//CTH//ZNF268//EDN1//FGFR2//SATB2//ZNF281//GABPA//HEYL//PROX2//KCNIP3//ASCL1//NFKB1//PAX5//FOXP3//GATAD2B//UBE2D3//VLDLR//LRP8//BHLHE40//PAXBP1//THUMPD1//CAMKK2//GRIN2B//IL5RA//KIT//NCAM1//MAPK4//PSMC6//SPTBN2//YWHAB//H3F3B//PRKAA1//THBS1//CD81//UBE4B//MGRN1//TRIM21//UBE2E1//TNKS2//TTL//KIF2A//MARK1//MAP7D1//CNTN2//DDX39B//CDC5L//EIF4A2//PAN3//PTBP1//PPIH//SNRNP27//NOVA1//POLR2E//SNRPA//RND1//RHOF//MGST1//PAX8//SGK2//IGFBP7//PHYH//DLX5//IARS//SFRP1//TXNDC8//SLC27A4//ABCC8//RPP14//SEMA4G//FBXO45//NDN//FZD3//EHD3//REP15//MAS1//CDK2AP1//TSLP//OTP//LRRTM1//CD209//JAM3//RAG1//CCL16//LGALS3BP//TLN1//FERMT3//BCR//NDFIP1//EFNA3//FGF11//NDP//IL26//C1QA//CXCL14//P2RX2//PRDM4//ELK1//TLX2//NFYA//PLAG1//HIVEP3//CREB3L3//LSM11//LIN9//NUP214//TOMM34//GNAO1//GNAZ//PFDN1//SGTB//TMX1//CANX//BAG5//ANP32E//MICAL2//ARFIP2//MAEA//PALM//CCDC6//PRUNE2//DAPK3//FAM32A//GAS2//RTN4//CASP7//OGT//TNFSF14//UNC5A//ARHGEF6//TRABD2B//VPS26A//FZD2//PPP3CB//RALBP1//PTPRT//AKAP10//CHRM1//TAGAP//GABRR1//HTR3E//GRP//KCNIP2//ARHGAP40//IGFBP1//IL4R//NDFIP2//RASL11B//SMPD1//RERGL//SH3BP5//GNA14//NUDT4//CARHSP1//RIT2//OXSR1//VAV3//YKT6//SYT9//NLGN1//KLHL2//RNF38//TULP4//KLHL14//PEX5//RNF135//PPP1R3F//FBP2//SLC5A3//MOGAT2//NDUFA12//HAAO//ASPH//GPD2//CTNS//EEF1E1//ERH//MTAP//SLC23A2//DPYS//TET3//PURA//MCIDAS//XRCC4//KMT2E//HMGB3//ASF1B//ZNF618//ZNF519//ZNF781//EWSR1//FOXG1//ATP1B4//MYCBP//HOXC9//NAB1//CREBZF//PRDM12//GPBP1L1//ZNF17//ZNF322//ZNF468//ZNF682//SNAPC1//PTAFR//WDR3//C2ORF49//PPP4R2//SCNM1//EIF1B//EIF4G2//LARP1//MEX3B//PTPN18//METTL21A//ST8SIA5//EDEM3//GALNT10//SPRN//TECTB//PYURF//USP46//KDELR1//TIMM23//ACBD6//INSIG2//PCTP//PIK3C2A//PIP4K2C//CYP4F3//CYP7A1//CA7//ALAD//SYPL2//LETM2//CPLX1//VPS45//MYOM3//BRK1//CGNL1//DNAH12//TMED2//RAB2A//PARD6B//ITGA5//TTC28//TTK//DPEP3//ACTR2//PARVA//CLEC1A//TSPAN14//TSPAN18//GPR34//FFAR3//CYSLTR2//GPR3//GRIA3//NPVF//EVC//DBNL//RALGPS2//NPTX1//SLC1A6//BSN//LRRC38//LRRN1//UFM1//RGS5//LDOC1//ATP6V1G2//UBXN10//BMP10//DDX55//AKAP7//PPP1R26//ZNF804A//TRIM67//PIK3IP1//EXTL2//XYLT2//NDST2//NDST3//HS6ST1//CPLX3//RBMS3//ST3GAL3//SERBP1//TNFAIP8L2//AVL9//TAF8//PASD1//USP54//NREP//ZDHHC20//GOLGA7B//SCN1A//HLA-DPA1//MLPH//TOMM40L//ELAVL3//MEA1//SLC9B1//EXD1//TBC1D24//KRT9//KRTAP1-1//KRT38//RABL3//WDFY1//ARPC3//SRXN1//GPX8//DNAJB12//FAR2//C9ORF129//VSNL1//GOSR2//KCTD18//KCNA6//WDR91//TGOLN2//NUCB1//ANO8//STEAP2//FAM57B//MSI2//SULT4A1//SLC26A1//CLEC9A//IFRD2//SCN3B//SLC30A6//ANO3//KCNQ4//GRIPAP1//LYPD6//PATE1//ZSWIM6//FAM168A// |
| GO:0007166 | cell surface receptor signaling pathway | Biological process | 80 | 3016 | 372 | 17653 | 1.25873477653233 | 0.0150448700956099 | 0.715925035608703 | 1.82261155780781 | 0.21505376344086 | TSLP//CD209//NFKB1//PSMC6//TRABD2B//MARK1//NDP//PRKAA1//TNKS2//VPS26A//FZD2//GNAO1//PPP3CB//FZD3//PTPRT//PIK3C2A//GRIA3//GRIN2B//HEYL//ASCL1//EVC//VAV3//ITGA5//TLN1//FERMT3//IGFBP1//ATP6V1G2//FGFR2//POLR2E//PTBP1//BMP10//TTK//NREP//LRRTM1//IL4R//IL5RA//KIT//NDN//IL26//PTAFR//LRP8//HLA-DPA1//FOXP3//SFRP1//DLX5//UBE2D3//THBS1//RASL11B//TNFSF14//GPR34//P2RX2//MTAP//UNC5A//VLDLR//ARPC3//ACTR2//BRK1//PTPN18//ERH//TSPAN14//MGRN1//OGT//BCR//EFNA3//PAX5//CNTN2//NCAM1//TRIM21//CCL16//EDN1//CANX//SEMA4G//RBMS3//DAPK3//RTN4//NLGN1//CLEC1A//MAPK4//TSPAN18//CD81// |
| GO:0051155 | positive regulation of striated muscle cell differentiation | Biological process | 5 | 69 | 372 | 17653 | 3.43871746922238 | 0.0151224471824578 | 0.715925035608703 | 1.82037792366023 | 0.0134408602150538 | EDN1//BMP10//DDX39B//IL4R//TNFSF14// |
| GO:0042330 | taxis | Biological process | 21 | 595 | 372 | 17653 | 1.67485768500949 | 0.0151389052372503 | 0.715925035608703 | 1.81990552949986 | 0.0564516129032258 | CCL16//KIT//RALBP1//PTAFR//CXCL14//EFNA3//NCAM1//SPTBN2//CNTN2//VLDLR//UNC5A//THBS1//TNFSF14//VAV3//FZD3//SEMA4G//PARVA//JAM3//EDN1//DLX5//ZSWIM6// |
| GO:0019233 | sensory perception of pain | Biological process | 6 | 95 | 372 | 17653 | 2.99711375212224 | 0.0152147265719488 | 0.715925035608703 | 1.8177358480617 | 0.0161290322580645 | PRDM12//SCN1A//EDN1//PTAFR//NDN//SCN3B// |
| GO:0048839 | inner ear development | Biological process | 9 | 182 | 372 | 17653 | 2.34664126196384 | 0.0152437091716181 | 0.715925035608703 | 1.81690934554447 | 0.0241935483870968 | FGFR2//DLX5//INSIG2//BCR//PAX8//FZD3//KCNQ4//FZD2//CXCL14// |
| GO:0071774 | response to fibroblast growth factor | Biological process | 8 | 152 | 372 | 17653 | 2.4975947934352 | 0.0153696854775818 | 0.716863330240591 | 1.81333501973176 | 0.021505376344086 | FGFR2//POLR2E//PTBP1//THBS1//CPS1//SFRP1//CDC5L//ELK1// |
| GO:0045620 | negative regulation of lymphocyte differentiation | Biological process | 4 | 46 | 372 | 17653 | 4.12646096306685 | 0.0157865707906224 | 0.730427021522708 | 1.80171219858272 | 0.010752688172043 | HMGB3//SFRP1//IL4R//FOXP3// |
| GO:0097061 | dendritic spine organization | Biological process | 5 | 70 | 372 | 17653 | 3.38959293394777 | 0.0160128598675935 | 0.730427021522708 | 1.79553109695037 | 0.0134408602150538 | NLGN1//ACTR2//LRP8//GRIN2B//ZNF804A// |
| GO:0008015 | blood circulation | Biological process | 19 | 526 | 372 | 17653 | 1.71412874606484 | 0.0163328217709579 | 0.730427021522708 | 1.7869387770705 | 0.0510752688172043 | P2RX2//CHRM1//DDAH1//CYP4F2//EDN1//GNAO1//KCNIP2//PTAFR//SCN3B//PIK3C2A//CPS1//BCR//FFAR3//BMP10//EHD3//KCNIP3//CACNG1//SCN1A//ASPH// |
| GO:0031325 | positive regulation of cellular metabolic process | Biological process | 81 | 3074 | 372 | 17653 | 1.25042237706466 | 0.0167956400253988 | 0.730427021522708 | 1.77480344200375 | 0.217741935483871 | KIT//PRKAA1//THBS1//CD81//CAMKK2//CDK2AP1//HEYL//MAS1//DBNL//DDX39B//CASP7//TP53INP2//TRIM21//MICAL2//BMP10//TTK//EDN1//CNTN2//ARPP19//LARP1//RBMS3//PTAFR//TAF8//DLX5//ELK1//ZNF281//FZD2//NDP//NFKB1//NFYA//FOXP3//PPP3CB//KMT2E//HIVEP3//GPBP1L1//SFRP1//PAX8//PAXBP1//UBQLN1//NDFIP2//NDFIP1//OXSR1//TNKS2//IL26//INSIG2//FGFR2//SMPD1//TSLP//RIT2//VAV3//DDAH1//VLDLR//ASPH//OGT//PSMC6//ZNF268//PRDM4//TET3//SATB2//ATP1B4//GABPA//PROX2//TLX2//MCIDAS//ASCL1//NDN//PAX5//PLAG1//CREB3L3//CDC5L//ITGA5//LRP8//PRDM12//CYSLTR2//CCL16//EFNA3//TRABD2B//FAM168A//TRIM67//PIP4K2C//GRIN2B// |
| GO:0008306 | associative learning | Biological process | 5 | 71 | 372 | 17653 | 3.34185218839921 | 0.0169375255097777 | 0.730427021522708 | 1.77115003768573 | 0.0134408602150538 | CTNS//KIT//RAG1//ABCC8//ACTR2// |
| GO:0030001 | metal ion transport | Biological process | 28 | 868 | 372 | 17653 | 1.53078390565383 | 0.0170256009720772 | 0.730427021522708 | 1.76889754935641 | 0.0752688172043011 | KCNIP2//KCNA6//ABCC8//KCNQ4//SCN3B//SCN1A//SLC5A3//SLC23A2//SLC39A2//ATP1B4//P2RX2//STEAP2//REP15//ATP6V1G2//SLC9B1//BEST1//GNAO1//CACNG1//SLC30A6//LRRC38//KCNIP3//GRIN2B//OXSR1//UBQLN1//EHD3//SESTD1//NDFIP2//NDFIP1// |
| GO:0034762 | regulation of transmembrane transport | Biological process | 19 | 529 | 372 | 17653 | 1.70440778909283 | 0.0172271020905326 | 0.730427021522708 | 1.7637877725775 | 0.0510752688172043 | THBS1//ARPP19//KCNA6//SCN1A//CACNG1//KCNQ4//OXSR1//UBQLN1//KCNIP3//KCNIP2//EHD3//PTAFR//LRRC38//ABCC8//SESTD1//NLGN1//SCN3B//PRKAA1//FFAR3// |
| GO:0003013 | circulatory system process | Biological process | 19 | 530 | 372 | 17653 | 1.70119192533983 | 0.017533538870531 | 0.730427021522708 | 1.75613041951188 | 0.0510752688172043 | P2RX2//CHRM1//DDAH1//CYP4F2//EDN1//GNAO1//KCNIP2//PTAFR//SCN3B//PIK3C2A//CPS1//BCR//FFAR3//BMP10//EHD3//KCNIP3//CACNG1//SCN1A//ASPH// |
| GO:0002638 | negative regulation of immunoglobulin production | Biological process | 2 | 10 | 372 | 17653 | 9.49086021505376 | 0.0178205616666046 | 0.730427021522708 | 1.74907861203523 | 0.00537634408602151 | FOXP3//NDFIP1// |
| GO:0002713 | negative regulation of B cell mediated immunity | Biological process | 2 | 10 | 372 | 17653 | 9.49086021505376 | 0.0178205616666046 | 0.730427021522708 | 1.74907861203523 | 0.00537634408602151 | FOXP3//NDFIP1// |
| GO:0002890 | negative regulation of immunoglobulin mediated immune response | Biological process | 2 | 10 | 372 | 17653 | 9.49086021505376 | 0.0178205616666046 | 0.730427021522708 | 1.74907861203523 | 0.00537634408602151 | FOXP3//NDFIP1// |
| GO:0003149 | membranous septum morphogenesis | Biological process | 2 | 10 | 372 | 17653 | 9.49086021505376 | 0.0178205616666046 | 0.730427021522708 | 1.74907861203523 | 0.00537634408602151 | FGFR2//FZD2// |
| GO:0021877 | forebrain neuron fate commitment | Biological process | 2 | 10 | 372 | 17653 | 9.49086021505376 | 0.0178205616666046 | 0.730427021522708 | 1.74907861203523 | 0.00537634408602151 | SATB2//ASCL1// |
| GO:0021892 | cerebral cortex GABAergic interneuron differentiation | Biological process | 2 | 10 | 372 | 17653 | 9.49086021505376 | 0.0178205616666046 | 0.730427021522708 | 1.74907861203523 | 0.00537634408602151 | CNTN2//ASCL1// |
| GO:0032905 | transforming growth factor beta1 production | Biological process | 2 | 10 | 372 | 17653 | 9.49086021505376 | 0.0178205616666046 | 0.730427021522708 | 1.74907861203523 | 0.00537634408602151 | FOXP3//THBS1// |
| GO:0032908 | regulation of transforming growth factor beta1 production | Biological process | 2 | 10 | 372 | 17653 | 9.49086021505376 | 0.0178205616666046 | 0.730427021522708 | 1.74907861203523 | 0.00537634408602151 | FOXP3//THBS1// |
| GO:0042758 | long-chain fatty acid catabolic process | Biological process | 2 | 10 | 372 | 17653 | 9.49086021505376 | 0.0178205616666046 | 0.730427021522708 | 1.74907861203523 | 0.00537634408602151 | CYP4F3//CYP4F2// |
| GO:1901569 | fatty acid derivative catabolic process | Biological process | 2 | 10 | 372 | 17653 | 9.49086021505376 | 0.0178205616666046 | 0.730427021522708 | 1.74907861203523 | 0.00537634408602151 | CYP4F3//CYP4F2// |
| GO:1902950 | regulation of dendritic spine maintenance | Biological process | 2 | 10 | 372 | 17653 | 9.49086021505376 | 0.0178205616666046 | 0.730427021522708 | 1.74907861203523 | 0.00537634408602151 | GRIN2B//ZNF804A// |
| GO:2001225 | regulation of chloride transport | Biological process | 2 | 10 | 372 | 17653 | 9.49086021505376 | 0.0178205616666046 | 0.730427021522708 | 1.74907861203523 | 0.00537634408602151 | PTAFR//CA7// |
| GO:0008542 | visual learning | Biological process | 4 | 48 | 372 | 17653 | 3.95452508960574 | 0.0182198282737365 | 0.733456539376666 | 1.73945572067367 | 0.010752688172043 | CTNS//KIT//RAG1//ABCC8// |
| GO:0045747 | positive regulation of Notch signaling pathway | Biological process | 4 | 48 | 372 | 17653 | 3.95452508960574 | 0.0182198282737365 | 0.733456539376666 | 1.73945572067367 | 0.010752688172043 | ERH//KIT//ASCL1//TSPAN14// |
| GO:1903307 | positive regulation of regulated secretory pathway | Biological process | 4 | 48 | 372 | 17653 | 3.95452508960574 | 0.0182198282737365 | 0.733456539376666 | 1.73945572067367 | 0.010752688172043 | IL4R//PTAFR//SYT9//NLGN1// |
| GO:0070887 | cellular response to chemical stimulus | Biological process | 83 | 3175 | 372 | 17653 | 1.2405376344086 | 0.0185926302857885 | 0.742715999393868 | 1.73065916655954 | 0.223118279569892 | TSLP//CCL16//KIT//P2RX2//MGST1//CHRM1//IGFBP1//PIK3C2A//ATP6V1G2//FGFR2//POLR2E//PTBP1//THBS1//TNFSF14//NREP//LRRTM1//IL4R//IL5RA//NDN//IL26//PTAFR//LRP8//DLX5//BMP10//UBE2D3//RASL11B//SFRP1//VAV3//ITGA5//CTH//EDEM3//CREB3L3//IGFBP7//UFM1//PSMC6//SRXN1//TXNDC8//GPX8//CAMKK2//EDN1//PPP3CB//MTAP//VSNL1//ACTR2//EXTL2//TLN1//GOSR2//MAS1//CPS1//CDC5L//OGT//CXCL14//HLA-DPA1//NCAM1//TRIM21//HEYL//CANX//PRKAA1//NFKB1//DNAJB12//BCR//NLGN1//ALAD//XRCC4//SATB2//MAPK4//ABCC8//CYP7A1//PTPN18//DAPK3//PAX8//ELK1//FFAR3//UBQLN1//PARVA//CASP7//LARP1//JAM3//DDAH1//BAG5//GABPA//ARPC3//EIF4A2// |
| GO:0007628 | adult walking behavior | Biological process | 3 | 27 | 372 | 17653 | 5.27270011947431 | 0.0186694839415877 | 0.742715999393868 | 1.72886768657252 | 0.00806451612903226 | CTNS//SCN1A//CNTN2// |
| GO:0106027 | neuron projection organization | Biological process | 5 | 73 | 372 | 17653 | 3.25029459419649 | 0.0188915883006857 | 0.747156793435891 | 1.72373152745531 | 0.0134408602150538 | NLGN1//ACTR2//LRP8//GRIN2B//ZNF804A// |
| GO:0016051 | carbohydrate biosynthetic process | Biological process | 10 | 221 | 372 | 17653 | 2.14725344231986 | 0.0190919836410376 | 0.750692356769403 | 1.71914894643077 | 0.0268817204301075 | PPP1R3F//FBP2//GPD2//GALNT10//NDST3//ARPP19//MAEA//MAS1//PTAFR//NFKB1// |
| GO:0023051 | regulation of signaling | Biological process | 92 | 3575 | 372 | 17653 | 1.22120159410482 | 0.0192486939824606 | 0.752479291349023 | 1.71559873189326 | 0.247311827956989 | KIT//PRKAA1//THBS1//CD81//EHD3//REP15//TSLP//MAS1//DBNL//SYT9//PPP3CB//ABCC8//RGS5//FGFR2//VAV3//EDN1//FGF11//BMP10//GRP//NDP//IL26//CCL16//TNFSF14//CXCL14//AKAP7//TTK//PIP4K2C//PIK3IP1//MGRN1//YWHAB//NREP//SMPD1//FOXP3//SFRP1//TRABD2B//RASL11B//ITGA5//USP46//NLGN1//CA7//NPVF//RALGPS2//RIT2//UFM1//UBQLN1//WDFY1//OGT//BCR//ARHGEF6//VSNL1//CTH//NDFIP2//NDFIP1//EEF1E1//IGFBP1//ERH//ASCL1//TSPAN14//EVC//LRRTM1//TRIM67//CPLX1//CPLX3//GRIN2B//CNTN2//GRIA3//LRP8//P2RX2//PAX5//RALBP1//TAGAP//ARHGAP40//RHOF//CGNL1//RTN4//PALM//HEYL//CYSLTR2//RBMS3//PSMC6//DAPK3//DLX5//NFKB1//TNKS2//FFAR3//LYPD6//PATE1//PTPN18//BAG5//LDOC1//ASPH//PAX8// |
| GO:0010171 | body morphogenesis | Biological process | 4 | 49 | 372 | 17653 | 3.87382049594031 | 0.0195189175973699 | 0.754322512634358 | 1.70954426938055 | 0.010752688172043 | ZNF281//DLX5//ASPH//CRISPLD1// |
| GO:0060421 | positive regulation of heart growth | Biological process | 4 | 49 | 372 | 17653 | 3.87382049594031 | 0.0195189175973699 | 0.754322512634358 | 1.70954426938055 | 0.010752688172043 | FGFR2//BMP10//EDN1//DDX39B// |
| GO:0048568 | embryonic organ development | Biological process | 16 | 428 | 372 | 17653 | 1.77399256356145 | 0.019804579712154 | 0.761013480643736 | 1.70323436965096 | 0.043010752688172 | TMED2//MICAL2//FGFR2//KIT//DLX5//INSIG2//BCR//PAX8//FZD3//KCNQ4//EDN1//PAX5//SATB2//HOXC9//HS6ST1//FZD2// |
| GO:1903725 | regulation of phospholipid metabolic process | Biological process | 5 | 74 | 372 | 17653 | 3.20637169427492 | 0.019921927668695 | 0.761197722166013 | 1.70066864104421 | 0.0134408602150538 | CD81//WDR91//VAV3//KIT//PIK3IP1// |
| GO:0030968 | endoplasmic reticulum unfolded protein response | Biological process | 7 | 130 | 372 | 17653 | 2.55523159636063 | 0.0202912562249613 | 0.770953740727041 | 1.69269106510661 | 0.0188172043010753 | EXTL2//TLN1//GOSR2//IGFBP1//CTH//EDEM3//CREB3L3// |
| GO:0090659 | walking behavior | Biological process | 3 | 28 | 372 | 17653 | 5.08438940092166 | 0.020590319456072 | 0.7779459803431 | 1.68633691530849 | 0.00806451612903226 | CTNS//SCN1A//CNTN2// |
| GO:0002504 | antigen processing and presentation of peptide or polysaccharide antigen via MHC class II | Biological process | 6 | 102 | 372 | 17653 | 2.79142947501581 | 0.0208874559740934 | 0.784224171173617 | 1.6801144524895 | 0.0161290322580645 | THBS1//AP1S1//HLA-DPA1//KIF2A//SPTBN2//CANX// |
| GO:0098876 | vesicle-mediated transport to the plasma membrane | Biological process | 5 | 75 | 372 | 17653 | 3.16362007168459 | 0.0209884038122763 | 0.784224171173617 | 1.67802058866294 | 0.0134408602150538 | STEAP2//SYS1//GRIPAP1//ANKRD50//VPS26A// |
| GO:0022604 | regulation of cell morphogenesis | Biological process | 17 | 468 | 372 | 17653 | 1.72376734675122 | 0.0213227737661294 | 0.785067932969694 | 1.67115630091335 | 0.0456989247311828 | DAPK3//GAS2//RND1//KIT//PALM//RHOF//PARVA//SEPT7//TTL//RTN4//CNTN2//SEMA4G//TLX2//NLGN1//ACTR2//LRP8//ZSWIM6// |
| GO:0044650 | adhesion of symbiont to host cell | Biological process | 2 | 11 | 372 | 17653 | 8.62805474095797 | 0.0214805646190487 | 0.785067932969694 | 1.66795430734332 | 0.00537634408602151 | CD209//CD81// |
| GO:0060525 | prostate glandular acinus development | Biological process | 2 | 11 | 372 | 17653 | 8.62805474095797 | 0.0214805646190487 | 0.785067932969694 | 1.66795430734332 | 0.00537634408602151 | FGFR2//SFRP1// |
| GO:1902563 | regulation of neutrophil activation | Biological process | 2 | 11 | 372 | 17653 | 8.62805474095797 | 0.0214805646190487 | 0.785067932969694 | 1.66795430734332 | 0.00537634408602151 | BCR//PTAFR// |
| GO:0009893 | positive regulation of metabolic process | Biological process | 86 | 3329 | 372 | 17653 | 1.22591465679577 | 0.0218179759729038 | 0.785067932969694 | 1.66118554084991 | 0.231182795698925 | KIT//PRKAA1//THBS1//CD81//CAMKK2//CDK2AP1//HEYL//MAS1//DBNL//DDX39B//CASP7//TP53INP2//TRIM21//TMED2//GABPA//RBMS3//ZNF804A//MICAL2//BMP10//TTK//EDN1//CNTN2//ARPP19//LARP1//PTAFR//TAF8//DLX5//ELK1//ZNF281//FZD2//NDP//NFKB1//NFYA//FOXP3//PPP3CB//KMT2E//HIVEP3//GPBP1L1//SFRP1//PAX8//PAXBP1//UBQLN1//NDFIP2//NDFIP1//OXSR1//TNKS2//IL26//INSIG2//FGFR2//SMPD1//TSLP//RIT2//VAV3//DDAH1//H3F3B//POLR2E//VLDLR//ASPH//OGT//PSMC6//ZNF268//PRDM4//TET3//SATB2//ATP1B4//PROX2//TLX2//MCIDAS//ASCL1//NDN//PAX5//PLAG1//CREB3L3//CDC5L//ITGA5//LRP8//PRDM12//CYSLTR2//CCL16//EFNA3//TRABD2B//FAM168A//TRIM67//PIP4K2C//GRIN2B//PYURF// |
| GO:0016571 | histone methylation | Biological process | 7 | 132 | 372 | 17653 | 2.51651596611274 | 0.0218562631788686 | 0.785067932969694 | 1.66042408847613 | 0.0188172043010753 | PAXBP1//KMT2E//PRDM4//PAX5//PRDM12//OGT//TET3// |
| GO:0050767 | regulation of neurogenesis | Biological process | 24 | 732 | 372 | 17653 | 1.55587872377931 | 0.0219753749665429 | 0.785067932969694 | 1.65806370581 | 0.0645161290322581 | OTP//FZD3//SFRP1//ZNF804A//TRIM67//RIT2//BAG5//TTL//RTN4//CNTN2//EFNA3//NLGN1//NREP//ASCL1//HEYL//KIT//SEMA4G//TLX2//PLAG1//ABCC8//ACTR2//LRP8//VLDLR//ZSWIM6// |
| GO:0042493 | response to drug | Biological process | 30 | 966 | 372 | 17653 | 1.47373605823816 | 0.0219958396657014 | 0.785067932969694 | 1.65765945468187 | 0.0806451612903226 | SLC1A6//RALBP1//CHRM1//EDN1//ALAD//PRKAA1//THBS1//P2RX2//PPP3CB//SMPD1//GNAO1//ACTR2//FGFR2//GRIN2B//USP46//IGFBP7//ASCL1//NFKB1//SFRP1//CPS1//PTAFR//NUCB1//LARP1//GABPA//VAV3//MAS1//MGST1//ABCC8//LRP8//FZD3// |
| GO:0045010 | actin nucleation | Biological process | 4 | 51 | 372 | 17653 | 3.72190596668775 | 0.0222850367799821 | 0.785067932969694 | 1.65198664480284 | 0.010752688172043 | ARPC3//ACTR2//ARFIP2//BRK1// |
| GO:0045935 | positive regulation of nucleobase-containing compound metabolic process | Biological process | 49 | 1742 | 372 | 17653 | 1.33482247571078 | 0.0223304482378515 | 0.785067932969694 | 1.65110255927197 | 0.131720430107527 | HEYL//DDX39B//MICAL2//CAMKK2//TAF8//DLX5//ELK1//ZNF281//FZD2//BMP10//NDP//NFKB1//NFYA//FOXP3//PPP3CB//KMT2E//TP53INP2//HIVEP3//GPBP1L1//SFRP1//PAX8//TNKS2//INSIG2//MAS1//PRKAA1//PSMC6//ZNF268//PRDM4//EDN1//TET3//FGFR2//SATB2//ATP1B4//GABPA//PROX2//TLX2//MCIDAS//ASCL1//NDN//PAX5//PLAG1//IL26//RIT2//CREB3L3//OGT//PAXBP1//CD81//CDC5L//FAM168A// |
| GO:0008360 | regulation of cell shape | Biological process | 8 | 163 | 372 | 17653 | 2.32904545154694 | 0.0223815027156653 | 0.785067932969694 | 1.65011075787862 | 0.021505376344086 | SEPT7//DAPK3//GAS2//RND1//KIT//PALM//RHOF//PARVA// |
| GO:0015031 | protein transport | Biological process | 53 | 1914 | 372 | 17653 | 1.3140428197436 | 0.0231924755919285 | 0.785067932969694 | 1.63465289178757 | 0.14247311827957 | SPRN//NUP214//DDX39B//NLGN1//PAN3//YWHAB//ZDHHC20//GOLGA7B//GOSR2//TIMM23//TOMM34//KDELR1//TMED2//VPS45//AP1S1//ARFIP2//RABL3//KCNIP3//RAB2A//MLPH//VPS26A//SYT9//PPP3CB//ABCC8//CANX//PEX5//CPLX1//CPLX3//TOMM40L//REP15//ATP6V1G2//VLDLR//VSNL1//SYS1//SFRP1//FOXP3//CLEC9A//IL26//NDFIP2//NDFIP1//BCR//IL4R//CHRM1//GRIPAP1//NUCB1//PRKAA1//UBE2D3//TSLP//YKT6//EHD3//VPS53//ANKRD50//ZW10// |
| GO:0010508 | positive regulation of autophagy | Biological process | 6 | 105 | 372 | 17653 | 2.71167434715822 | 0.0237059198470964 | 0.785067932969694 | 1.62514318834049 | 0.0161290322580645 | LARP1//CAMKK2//PIP4K2C//PRKAA1//TP53INP2//TRIM21// |
| GO:0065002 | intracellular protein transmembrane transport | Biological process | 4 | 52 | 372 | 17653 | 3.65033085194376 | 0.0237531249642837 | 0.785067932969694 | 1.62427924644051 | 0.010752688172043 | PEX5//TIMM23//TOMM40L//BCR// |
| GO:0032956 | regulation of actin cytoskeleton organization | Biological process | 13 | 331 | 372 | 17653 | 1.86376409056947 | 0.0238022199267793 | 0.785067932969694 | 1.62338253634726 | 0.0349462365591398 | BRK1//ARHGAP40//ARPC3//ACTR2//ARFIP2//DAPK3//SFRP1//SPTBN2//EDN1//BMP10//JAM3//RND1//RHOF// |
| GO:0044089 | positive regulation of cellular component biogenesis | Biological process | 18 | 511 | 372 | 17653 | 1.67158007701534 | 0.0238480026156462 | 0.785067932969694 | 1.62254798934205 | 0.0483870967741935 | BRK1//PAN3//MAPRE1//KIT//TRABD2B//ARPC3//ACTR2//ARFIP2//PSMC6//NLGN1//PALM//SFRP1//LRRTM1//LRRN1//EDN1//BMP10//SEPT7//PIP4K2C// |
| GO:0050807 | regulation of synapse organization | Biological process | 8 | 165 | 372 | 17653 | 2.30081459758879 | 0.0238645908557842 | 0.785067932969694 | 1.62224600695449 | 0.021505376344086 | NLGN1//LRRTM1//LRRN1//ACTR2//LRP8//GRIN2B//ZNF804A//GRIPAP1// |
| GO:0035967 | cellular response to topologically incorrect protein | Biological process | 8 | 166 | 372 | 17653 | 2.28695426868765 | 0.0246314332242909 | 0.785067932969694 | 1.6085103172193 | 0.021505376344086 | CTH//EDEM3//CREB3L3//EXTL2//TLN1//GOSR2//IGFBP1//DNAJB12// |
| GO:0060325 | face morphogenesis | Biological process | 3 | 30 | 372 | 17653 | 4.74543010752688 | 0.0247453627295478 | 0.785067932969694 | 1.60650617570668 | 0.00806451612903226 | DLX5//ASPH//CRISPLD1// |
| GO:0035966 | response to topologically incorrect protein | Biological process | 9 | 198 | 372 | 17653 | 2.15701368523949 | 0.0248048424822526 | 0.785067932969694 | 1.60546352651152 | 0.0241935483870968 | THBS1//CTH//EDEM3//CREB3L3//EXTL2//TLN1//GOSR2//IGFBP1//DNAJB12// |
| GO:0060322 | head development | Biological process | 24 | 741 | 372 | 17653 | 1.53698141134474 | 0.0249468791221184 | 0.785067932969694 | 1.60298377728784 | 0.0645161290322581 | CTNS//FOXG1//GRIN2B//H3F3B//CA10//BCR//LRP8//PAX5//SPTBN2//ASCL1//SYPL2//MAS1//FGFR2//FBXO45//RTN4//CNTN2//OTP//DLX5//SATB2//GNAO1//FZD3//ASPH//CRISPLD1//SFRP1// |
| GO:0060284 | regulation of cell development | Biological process | 27 | 858 | 372 | 17653 | 1.49331716670426 | 0.0250051325677961 | 0.785067932969694 | 1.60197083864442 | 0.0725806451612903 | OTP//FZD3//SFRP1//ZNF804A//TRIM67//RIT2//BAG5//TTL//RTN4//CNTN2//EFNA3//NLGN1//NREP//ASCL1//HEYL//KIT//SEMA4G//TLX2//PLAG1//ABCC8//EDN1//BMP10//ACTR2//LRP8//DDX39B//VLDLR//ZSWIM6// |
| GO:0016192 | vesicle-mediated transport | Biological process | 56 | 2050 | 372 | 17653 | 1.29631261473905 | 0.0250849020103693 | 0.785067932969694 | 1.60058759112573 | 0.150537634408602 | LRRTM1//LGALS3BP//THBS1//TLN1//FERMT3//VAV3//YKT6//SYT9//NLGN1//CPLX1//PIK3C2A//KDELR1//TMED2//SPTBN2//ZW10//GOSR2//KIF2A//STEAP2//TGOLN2//SYS1//RALBP1//CD209//LRP8//AP1S1//CLEC9A//DBNL//TMPRSS2//VLDLR//VPS45//MGRN1//CPLX3//PPP3CB//PTAFR//RIT2//CNTN2//CD81//EHD3//VPS53//ARPC3//ACTR2//BRK1//VPS26A//KIT//IL4R//ALAD//MGST1//NFKB1//RHOF//TSPAN14//BCR//WDR91//VSNL1//CANX//PRKAA1//GRIPAP1//ANKRD50// |
| GO:0007612 | learning | Biological process | 7 | 136 | 372 | 17653 | 2.44250079063884 | 0.0252334116448426 | 0.785067932969694 | 1.59802402739118 | 0.0188172043010753 | ACTR2//CTNS//KIT//RAG1//ABCC8//PPP3CB//CNTN2// |
| GO:0007632 | visual behavior | Biological process | 4 | 53 | 372 | 17653 | 3.58145668492595 | 0.0252785221893528 | 0.785067932969694 | 1.59724831898812 | 0.010752688172043 | CTNS//KIT//RAG1//ABCC8// |
| GO:0010038 | response to metal ion | Biological process | 13 | 334 | 372 | 17653 | 1.84702369454639 | 0.0253777016728383 | 0.785067932969694 | 1.595547712265 | 0.0349462365591398 | KCNIP2//ALAD//C1QA//CPS1//HAAO//SLC30A6//ABCC8//ASCL1//KIT//THBS1//EDN1//NLGN1//XRCC4// |
| GO:0009086 | methionine biosynthetic process | Biological process | 2 | 12 | 372 | 17653 | 7.90905017921147 | 0.0254221317936364 | 0.785067932969694 | 1.59478803413631 | 0.00537634408602151 | MTAP//CTH// |
| GO:0014831 | gastro-intestinal system smooth muscle contraction | Biological process | 2 | 12 | 372 | 17653 | 7.90905017921147 | 0.0254221317936364 | 0.785067932969694 | 1.59478803413631 | 0.00537634408602151 | KIT//PTAFR// |
| GO:0033631 | cell-cell adhesion mediated by integrin | Biological process | 2 | 12 | 372 | 17653 | 7.90905017921147 | 0.0254221317936364 | 0.785067932969694 | 1.59478803413631 | 0.00537634408602151 | FERMT3//ITGA5// |
| GO:0045161 | neuronal ion channel clustering | Biological process | 2 | 12 | 372 | 17653 | 7.90905017921147 | 0.0254221317936364 | 0.785067932969694 | 1.59478803413631 | 0.00537634408602151 | KCNIP2//CNTN2// |
| GO:0048266 | behavioral response to pain | Biological process | 2 | 12 | 372 | 17653 | 7.90905017921147 | 0.0254221317936364 | 0.785067932969694 | 1.59478803413631 | 0.00537634408602151 | P2RX2//THBS1// |
| GO:0048302 | regulation of isotype switching to IgG isotypes | Biological process | 2 | 12 | 372 | 17653 | 7.90905017921147 | 0.0254221317936364 | 0.785067932969694 | 1.59478803413631 | 0.00537634408602151 | FOXP3//NDFIP1// |
| GO:0060442 | branching involved in prostate gland morphogenesis | Biological process | 2 | 12 | 372 | 17653 | 7.90905017921147 | 0.0254221317936364 | 0.785067932969694 | 1.59478803413631 | 0.00537634408602151 | FGFR2//SFRP1// |
| GO:0060736 | prostate gland growth | Biological process | 2 | 12 | 372 | 17653 | 7.90905017921147 | 0.0254221317936364 | 0.785067932969694 | 1.59478803413631 | 0.00537634408602151 | FGFR2//PLAG1// |
| GO:0060766 | negative regulation of androgen receptor signaling pathway | Biological process | 2 | 12 | 372 | 17653 | 7.90905017921147 | 0.0254221317936364 | 0.785067932969694 | 1.59478803413631 | 0.00537634408602151 | HEYL//SFRP1// |
| GO:0097090 | presynaptic membrane organization | Biological process | 2 | 12 | 372 | 17653 | 7.90905017921147 | 0.0254221317936364 | 0.785067932969694 | 1.59478803413631 | 0.00537634408602151 | NLGN1//CNTN2// |
| GO:1904251 | regulation of bile acid metabolic process | Biological process | 2 | 12 | 372 | 17653 | 7.90905017921147 | 0.0254221317936364 | 0.785067932969694 | 1.59478803413631 | 0.00537634408602151 | CYP7A1//KIT// |
| GO:1905065 | positive regulation of vascular smooth muscle cell differentiation | Biological process | 2 | 12 | 372 | 17653 | 7.90905017921147 | 0.0254221317936364 | 0.785067932969694 | 1.59478803413631 | 0.00537634408602151 | CTH//KIT// |
| GO:0006024 | glycosaminoglycan biosynthetic process | Biological process | 6 | 107 | 372 | 17653 | 2.66098884534218 | 0.0257201577586395 | 0.786072550538652 | 1.58972637192602 | 0.0161290322580645 | ST3GAL3//XYLT2//NDST2//NDST3//NFKB1//HS6ST1// |
| GO:0006821 | chloride transport | Biological process | 6 | 107 | 372 | 17653 | 2.66098884534218 | 0.0257201577586395 | 0.786072550538652 | 1.58972637192602 | 0.0161290322580645 | BEST1//SLC26A1//GABRR1//PTAFR//CA7//ANO8// |
| GO:0050804 | modulation of chemical synaptic transmission | Biological process | 13 | 335 | 372 | 17653 | 1.84151019098058 | 0.0259195887579653 | 0.786072550538652 | 1.58637189329385 | 0.0349462365591398 | USP46//NLGN1//CA7//EDN1//CPLX1//CPLX3//GRIN2B//PPP3CB//CNTN2//KIT//LRRTM1//GRIA3//LRP8// |
| GO:0099177 | regulation of trans-synaptic signaling | Biological process | 13 | 335 | 372 | 17653 | 1.84151019098058 | 0.0259195887579653 | 0.786072550538652 | 1.58637189329385 | 0.0349462365591398 | USP46//NLGN1//CA7//EDN1//CPLX1//CPLX3//GRIN2B//PPP3CB//CNTN2//KIT//GRIA3//LRP8//LRRTM1// |
| GO:0090263 | positive regulation of canonical Wnt signaling pathway | Biological process | 7 | 137 | 372 | 17653 | 2.42467231771447 | 0.0261304804266642 | 0.788930531810402 | 1.58285260539969 | 0.0188172043010753 | DAPK3//DLX5//FGFR2//NFKB1//PSMC6//SFRP1//TNKS2// |
| GO:0007405 | neuroblast proliferation | Biological process | 4 | 54 | 372 | 17653 | 3.51513341298288 | 0.0268616348985194 | 0.788976849340144 | 1.57086755809331 | 0.010752688172043 | OTP//FZD3//FGFR2//ASCL1// |
| GO:0007010 | cytoskeleton organization | Biological process | 38 | 1306 | 372 | 17653 | 1.38075301750399 | 0.0268761959633757 | 0.788976849340144 | 1.57063220098376 | 0.102150537634409 | SEPT7//ZW10//TTL//KIF2A//MARK1//MAP7D1//CNTN2//RND1//RHOF//MICAL2//ARFIP2//BCR//MYOM3//BRK1//CGNL1//TLN1//TTK//ARHGAP40//SKA3//MAPRE1//KIT//PARVA//ARPC3//ACTR2//DAPK3//KRT9//BMP10//CEP85//NLGN1//SFRP1//SPTBN2//EDN1//JAM3//MCIDAS//MAEA//PALM//PFDN1//CCDC6// |
| GO:0034694 | response to prostaglandin | Biological process | 3 | 31 | 372 | 17653 | 4.5923517169615 | 0.0269794686307255 | 0.788976849340144 | 1.56896660814878 | 0.00806451612903226 | EDN1//PRKAA1//SFRP1// |
| GO:0051173 | positive regulation of nitrogen compound metabolic process | Biological process | 77 | 2965 | 372 | 17653 | 1.23237139386027 | 0.0269836538038062 | 0.788976849340144 | 1.56889924371558 | 0.206989247311828 | KIT//PRKAA1//THBS1//CD81//CAMKK2//CDK2AP1//HEYL//MAS1//DBNL//DDX39B//CASP7//MICAL2//BMP10//TTK//CNTN2//LARP1//RBMS3//PTAFR//TAF8//DLX5//ELK1//ZNF281//FZD2//NDP//NFKB1//NFYA//FOXP3//PPP3CB//KMT2E//TP53INP2//HIVEP3//GPBP1L1//SFRP1//PAX8//PAXBP1//UBQLN1//NDFIP2//NDFIP1//OXSR1//TNKS2//IL26//INSIG2//SMPD1//TSLP//EDN1//FGFR2//RIT2//DDAH1//VLDLR//ASPH//OGT//PSMC6//ZNF268//PRDM4//TET3//SATB2//ATP1B4//GABPA//PROX2//TLX2//MCIDAS//ASCL1//NDN//PAX5//PLAG1//CREB3L3//CDC5L//ITGA5//LRP8//PRDM12//CYSLTR2//CCL16//EFNA3//TRABD2B//FAM168A//TRIM67//GRIN2B// |
| GO:1901698 | response to nitrogen compound | Biological process | 32 | 1065 | 372 | 17653 | 1.425856933717 | 0.0276258315584324 | 0.788976849340144 | 1.55868464051758 | 0.0860215053763441 | CHRM1//IGFBP1//PIK3C2A//ATP6V1G2//MGST1//ALAD//CPS1//UBE4B//UBQLN1//PSMC6//PRKAA1//INSIG2//ABCC8//OGT//P2RX2//ACTR2//DNAJB12//MAS1//SMPD1//EDN1//GNAO1//ASCL1//PTAFR//NFKB1//CD81//SFRP1//CASP7//LARP1//GABPA//EDEM3//CDC5L//FFAR3// |
| GO:0034765 | regulation of ion transmembrane transport | Biological process | 16 | 446 | 372 | 17653 | 1.70239645113072 | 0.0276676182283248 | 0.788976849340144 | 1.55802822556513 | 0.043010752688172 | THBS1//OXSR1//UBQLN1//KCNIP3//KCNIP2//EHD3//PTAFR//LRRC38//ABCC8//SESTD1//NLGN1//SCN3B//KCNA6//SCN1A//CACNG1//KCNQ4// |
| GO:0045921 | positive regulation of exocytosis | Biological process | 5 | 81 | 372 | 17653 | 2.9292778441524 | 0.0281686540548332 | 0.788976849340144 | 1.55023390383154 | 0.0134408602150538 | IL4R//PTAFR//SYT9//NLGN1//VSNL1// |
| GO:0060415 | muscle tissue morphogenesis | Biological process | 5 | 81 | 372 | 17653 | 2.9292778441524 | 0.0281686540548332 | 0.788976849340144 | 1.55023390383154 | 0.0134408602150538 | FZD2//UBE4B//MYOM3//BMP10//FGFR2// |
| GO:0097581 | lamellipodium organization | Biological process | 5 | 81 | 372 | 17653 | 2.9292778441524 | 0.0281686540548332 | 0.788976849340144 | 1.55023390383154 | 0.0134408602150538 | BRK1//VAV3//ARFIP2//KIT//ARHGEF6// |
| GO:1901379 | regulation of potassium ion transmembrane transport | Biological process | 5 | 81 | 372 | 17653 | 2.9292778441524 | 0.0281686540548332 | 0.788976849340144 | 1.55023390383154 | 0.0134408602150538 | OXSR1//LRRC38//ABCC8//KCNIP3//KCNIP2// |
| GO:0030154 | cell differentiation | Biological process | 103 | 4124 | 372 | 17653 | 1.18520684062868 | 0.0283672778772931 | 0.788976849340144 | 1.54718233708363 | 0.276881720430108 | DLX5//IARS//TP53INP2//SFRP1//TXNDC8//SEMA4G//FBXO45//SATB2//MARK1//ASCL1//NDN//FZD3//FGFR2//OTP//KIT//JAM3//RAG1//FOXP3//NDFIP1//HEYL//PAX8//LRRC38//RTN4//LRRN1//PARD6B//EFNA3//NCAM1//SPTBN2//CNTN2//VLDLR//UNC5A//NFKB1//ZNF804A//TRIM67//RIT2//BAG5//EDN1//NAB1//RND1//DAPK3//FZD2//PROX2//KDELR1//PPP3CB//KMT2E//MYOM3//TTL//NREP//UBE4B//NLGN1//GNAO1//TBC1D24//PRDM12//KRT9//KRTAP1-1//KRT38//MGST1//PARVA//FERMT3//PAX5//HIVEP3//ITGA5//MAEA//KCNIP2//BMP10//C9ORF129//HMGB3//ZNF268//TAF8//FAM57B//IL4R//H3F3B//THBS1//GABPA//CXCL14//TNFSF14//HS6ST1//P2RX2//DBNL//ZNF281//MSI2//TLX2//PTBP1//PLAG1//ABCC8//NPTX1//ACTR2//LRP8//DDX39B//CPS1//PSMC6//ZSWIM6//MCIDAS//CTH//ELAVL3//ELK1//FOXG1//KIF2A//MEA1//ASF1B//PURA//CDC5L//SEPT7// |
| GO:0060688 | regulation of morphogenesis of a branching structure | Biological process | 4 | 55 | 372 | 17653 | 3.45122189638319 | 0.0285028213016564 | 0.788976849340144 | 1.54511214998804 | 0.010752688172043 | FGFR2//SFRP1//PAX8//RTN4// |
| GO:0019722 | calcium-mediated signaling | Biological process | 8 | 171 | 372 | 17653 | 2.22008426083129 | 0.0287254112075069 | 0.788976849340144 | 1.5417337456206 | 0.021505376344086 | PPP3CB//RIT2//P2RX2//CAMKK2//PRKAA1//NUDT4//EDN1//GRIN2B// |
| GO:0021953 | central nervous system neuron differentiation | Biological process | 8 | 171 | 372 | 17653 | 2.22008426083129 | 0.0287254112075069 | 0.788976849340144 | 1.5417337456206 | 0.021505376344086 | ASCL1//CNTN2//FGFR2//OTP//DLX5//SATB2//FBXO45//SFRP1// |
| GO:0071346 | cellular response to interferon-gamma | Biological process | 8 | 171 | 372 | 17653 | 2.22008426083129 | 0.0287254112075069 | 0.788976849340144 | 1.5417337456206 | 0.021505376344086 | HLA-DPA1//NCAM1//PTAFR//TRIM21//ACTR2//DAPK3//EDN1//CCL16// |
| GO:0062012 | regulation of small molecule metabolic process | Biological process | 17 | 485 | 372 | 17653 | 1.66334663562798 | 0.028738290729827 | 0.788976849340144 | 1.54153906600971 | 0.0456989247311828 | PPP1R3F//NUP214//OGT//PSMC6//PALM//NFKB1//ARPP19//THBS1//FZD2//NFYA//PRKAA1//INSIG2//MAEA//MAS1//PTAFR//CYP7A1//KIT// |
| GO:0010604 | positive regulation of macromolecule metabolic process | Biological process | 80 | 3106 | 372 | 17653 | 1.22226145718658 | 0.0288299611068658 | 0.788976849340144 | 1.5401559434975 | 0.21505376344086 | KIT//PRKAA1//THBS1//CD81//CAMKK2//CDK2AP1//HEYL//MAS1//DBNL//DDX39B//CASP7//TMED2//GABPA//RBMS3//ZNF804A//MICAL2//BMP10//TTK//EDN1//CNTN2//LARP1//PTAFR//TAF8//DLX5//ELK1//ZNF281//FZD2//NDP//NFKB1//NFYA//FOXP3//PPP3CB//KMT2E//TP53INP2//HIVEP3//GPBP1L1//SFRP1//PAX8//PAXBP1//UBQLN1//NDFIP2//NDFIP1//OXSR1//TNKS2//IL26//INSIG2//SMPD1//TSLP//FGFR2//RIT2//H3F3B//POLR2E//VLDLR//ASPH//OGT//PSMC6//ZNF268//PRDM4//TET3//SATB2//ATP1B4//PROX2//TLX2//MCIDAS//ASCL1//NDN//PAX5//PLAG1//CREB3L3//CDC5L//ITGA5//LRP8//PRDM12//CYSLTR2//CCL16//EFNA3//TRABD2B//FAM168A//TRIM67//GRIN2B// |
| GO:0008038 | neuron recognition | Biological process | 3 | 32 | 372 | 17653 | 4.44884072580645 | 0.0293176429005518 | 0.788976849340144 | 1.53287094932537 | 0.00806451612903226 | NDN//RTN4//CNTN2// |
| GO:0032228 | regulation of synaptic transmission, GABAergic | Biological process | 3 | 32 | 372 | 17653 | 4.44884072580645 | 0.0293176429005518 | 0.788976849340144 | 1.53287094932537 | 0.00806451612903226 | NLGN1//CA7//USP46// |
| GO:0010638 | positive regulation of organelle organization | Biological process | 21 | 637 | 372 | 17653 | 1.5644275079759 | 0.0293217195589463 | 0.788976849340144 | 1.53281056428113 | 0.0564516129032258 | PAN3//PAXBP1//MAPRE1//TNKS2//ARPC3//ACTR2//ARFIP2//FOXP3//EDN1//SFRP1//PRDM12//BMP10//OGT//YWHAB//SEPT7//CAMKK2//PRKAA1//UBE2D3//KMT2E//BRK1//PIP4K2C// |
| GO:0046879 | hormone secretion | Biological process | 12 | 306 | 372 | 17653 | 1.86095298334388 | 0.0294585018155516 | 0.788976849340144 | 1.53078934404409 | 0.032258064516129 | SYT9//PPP3CB//ABCC8//EDN1//CPLX1//CPLX3//NPVF//VSNL1//SFRP1//FFAR3//GRP//PAX8// |
| GO:0008284 | positive regulation of cell proliferation | Biological process | 28 | 911 | 372 | 17653 | 1.4585295610401 | 0.0296038267815502 | 0.788976849340144 | 1.52865214560698 | 0.0752688172043011 | OTP//FZD3//FGFR2//VAV3//CD81//FOXP3//RTN4//CD209//HLA-DPA1//EDN1//PTAFR//THBS1//DLX5//SFRP1//BMP10//PLAG1//DDX39B//ASCL1//PAXBP1//ZNF268//CHRM1//KIT//MAS1//PRKAA1//BRK1//PURA//TTK//TSLP// |
| GO:0032230 | positive regulation of synaptic transmission, GABAergic | Biological process | 2 | 13 | 372 | 17653 | 7.30066170388751 | 0.0296318379021731 | 0.788976849340144 | 1.52824141075357 | 0.00537634408602151 | NLGN1//CA7// |
| GO:0048291 | isotype switching to IgG isotypes | Biological process | 2 | 13 | 372 | 17653 | 7.30066170388751 | 0.0296318379021731 | 0.788976849340144 | 1.52824141075357 | 0.00537634408602151 | FOXP3//NDFIP1// |
| GO:0060732 | positive regulation of inositol phosphate biosynthetic process | Biological process | 2 | 13 | 372 | 17653 | 7.30066170388751 | 0.0296318379021731 | 0.788976849340144 | 1.52824141075357 | 0.00537634408602151 | MAS1//PTAFR// |
| GO:0061051 | positive regulation of cell growth involved in cardiac muscle cell development | Biological process | 2 | 13 | 372 | 17653 | 7.30066170388751 | 0.0296318379021731 | 0.788976849340144 | 1.52824141075357 | 0.00537634408602151 | EDN1//DDX39B// |
| GO:0061548 | ganglion development | Biological process | 2 | 13 | 372 | 17653 | 7.30066170388751 | 0.0296318379021731 | 0.788976849340144 | 1.52824141075357 | 0.00537634408602151 | ASCL1//FZD3// |
| GO:0071397 | cellular response to cholesterol | Biological process | 2 | 13 | 372 | 17653 | 7.30066170388751 | 0.0296318379021731 | 0.788976849340144 | 1.52824141075357 | 0.00537634408602151 | CYP7A1//LRP8// |
| GO:0090179 | planar cell polarity pathway involved in neural tube closure | Biological process | 2 | 13 | 372 | 17653 | 7.30066170388751 | 0.0296318379021731 | 0.788976849340144 | 1.52824141075357 | 0.00537634408602151 | FZD2//SFRP1// |
| GO:0097154 | GABAergic neuron differentiation | Biological process | 2 | 13 | 372 | 17653 | 7.30066170388751 | 0.0296318379021731 | 0.788976849340144 | 1.52824141075357 | 0.00537634408602151 | CNTN2//ASCL1// |
| GO:0007187 | G-protein coupled receptor signaling pathway, coupled to cyclic nucleotide second messenger | Biological process | 10 | 238 | 372 | 17653 | 1.99387819643987 | 0.0298832110961095 | 0.790852165109587 | 1.52457273729866 | 0.0268817204301075 | GNAO1//GNAZ//CYSLTR2//GNA14//GPR3//RIT2//EDN1//FFAR3//PALM//CHRM1// |
| GO:0008277 | regulation of G-protein coupled receptor protein signaling pathway | Biological process | 7 | 141 | 372 | 17653 | 2.35588728742469 | 0.0299361458329224 | 0.790852165109587 | 1.52380411418803 | 0.0188172043010753 | MGRN1//YWHAB//EDN1//PALM//CNTN2//GRP//RGS5// |
| GO:0061061 | muscle structure development | Biological process | 21 | 640 | 372 | 17653 | 1.55709425403226 | 0.0306273620380035 | 0.796801517461844 | 1.5138904077535 | 0.0564516129032258 | FZD2//UBE4B//EVC//PAXBP1//MYOM3//HEYL//PAX5//HIVEP3//BMP10//CXCL14//TNFSF14//PRKAA1//P2RX2//PTBP1//FGFR2//PROX2//EDN1//DDX39B//IL4R//CTH//KIT// |
| GO:0120035 | regulation of plasma membrane bounded cell projection organization | Biological process | 20 | 602 | 372 | 17653 | 1.57655485299896 | 0.0307309265590594 | 0.796801517461844 | 1.512424345262 | 0.0537634408602151 | BRK1//SFRP1//ZNF804A//TRIM67//RIT2//BAG5//TTL//RTN4//CNTN2//KIT//SEMA4G//TLX2//NLGN1//PALM//ACTR2//LRP8//VLDLR//ZSWIM6//SEPT7//GRIN2B// |
| GO:0031058 | positive regulation of histone modification | Biological process | 5 | 83 | 372 | 17653 | 2.85869283585957 | 0.030868253374982 | 0.796801517461844 | 1.51048794360075 | 0.0134408602150538 | PAXBP1//FOXP3//PRDM12//OGT//KMT2E// |
| GO:0044070 | regulation of anion transport | Biological process | 5 | 83 | 372 | 17653 | 2.85869283585957 | 0.030868253374982 | 0.796801517461844 | 1.51048794360075 | 0.0134408602150538 | THBS1//CYP4F2//EDN1//PTAFR//CA7// |
| GO:0045844 | positive regulation of striated muscle tissue development | Biological process | 5 | 83 | 372 | 17653 | 2.85869283585957 | 0.030868253374982 | 0.796801517461844 | 1.51048794360075 | 0.0134408602150538 | PRKAA1//FGFR2//BMP10//EDN1//DDX39B// |
| GO:0048636 | positive regulation of muscle organ development | Biological process | 5 | 83 | 372 | 17653 | 2.85869283585957 | 0.030868253374982 | 0.796801517461844 | 1.51048794360075 | 0.0134408602150538 | PRKAA1//FGFR2//BMP10//EDN1//DDX39B// |
| GO:0007611 | learning or memory | Biological process | 10 | 240 | 372 | 17653 | 1.97726254480287 | 0.0313888754593386 | 0.802770286708069 | 1.50322424309423 | 0.0268817204301075 | PPP3CB//CNTN2//ITGA5//ABCC8//VLDLR//CTNS//ACTR2//KIT//RAG1//GRIN2B// |
| GO:0032350 | regulation of hormone metabolic process | Biological process | 3 | 33 | 372 | 17653 | 4.31402737047898 | 0.0317594676931241 | 0.802770286708069 | 1.4981267852083 | 0.00806451612903226 | NFKB1//FFAR3//PAX8// |
| GO:0090218 | positive regulation of lipid kinase activity | Biological process | 3 | 33 | 372 | 17653 | 4.31402737047898 | 0.0317594676931241 | 0.802770286708069 | 1.4981267852083 | 0.00806451612903226 | CD81//VAV3//KIT// |
| GO:0048869 | cellular developmental process | Biological process | 107 | 4323 | 372 | 17653 | 1.17455707033397 | 0.0318603508760073 | 0.802770286708069 | 1.49674944565119 | 0.287634408602151 | DLX5//IARS//TP53INP2//SFRP1//TXNDC8//SEMA4G//FBXO45//SATB2//MARK1//ASCL1//NDN//FZD3//FGFR2//OTP//KIT//JAM3//RAG1//FOXP3//NDFIP1//HEYL//PAX8//LRRC38//RTN4//LRRN1//PARD6B//EFNA3//NCAM1//SPTBN2//CNTN2//VLDLR//UNC5A//DAPK3//GAS2//RND1//PALM//RHOF//PARVA//NFKB1//ZNF804A//TRIM67//RIT2//BAG5//EDN1//NAB1//SEPT7//ZNF268//TAF8//ELAVL3//ELK1//FOXG1//GABPA//KIF2A//MEA1//ASF1B//PURA//CDC5L//FZD2//PROX2//KDELR1//PPP3CB//KMT2E//MYOM3//TTL//NREP//UBE4B//NLGN1//GNAO1//TBC1D24//PRDM12//KRT9//KRTAP1-1//KRT38//MGST1//FERMT3//PAX5//HIVEP3//ITGA5//MAEA//KCNIP2//BMP10//C9ORF129//HMGB3//FAM57B//IL4R//H3F3B//THBS1//CXCL14//TNFSF14//HS6ST1//P2RX2//DBNL//ZNF281//MSI2//TLX2//PTBP1//PLAG1//ABCC8//NPTX1//ACTR2//LRP8//DDX39B//CPS1//PSMC6//ZSWIM6//MCIDAS//CTH//EEF1E1// |
| GO:1903902 | positive regulation of viral life cycle | Biological process | 4 | 57 | 372 | 17653 | 3.33012639124693 | 0.0319606103175485 | 0.802770286708069 | 1.49538493602223 | 0.010752688172043 | PPIH//LARP1//TRIM21//TMPRSS2// |
| GO:0001764 | neuron migration | Biological process | 7 | 143 | 372 | 17653 | 2.3229378148733 | 0.0319721392395564 | 0.802770286708069 | 1.49522830433348 | 0.0188172043010753 | CNTN2//FBXO45//SATB2//MARK1//ASCL1//NDN//FZD3// |
| GO:0001776 | leukocyte homeostasis | Biological process | 5 | 84 | 372 | 17653 | 2.82466077828981 | 0.0322768397997326 | 0.802770286708069 | 1.49110899330324 | 0.0134408602150538 | JAM3//FOXP3//PPP3CB//RAG1//TNFSF14// |
| GO:1901863 | positive regulation of muscle tissue development | Biological process | 5 | 84 | 372 | 17653 | 2.82466077828981 | 0.0322768397997326 | 0.802770286708069 | 1.49110899330324 | 0.0134408602150538 | PRKAA1//FGFR2//BMP10//EDN1//DDX39B// |
| GO:0006023 | aminoglycan biosynthetic process | Biological process | 6 | 113 | 372 | 17653 | 2.51969740222666 | 0.0324357659251296 | 0.802770286708069 | 1.48897584235747 | 0.0161290322580645 | XYLT2//NDST2//HS6ST1//ST3GAL3//NDST3//NFKB1// |
| GO:0010646 | regulation of cell communication | Biological process | 89 | 3522 | 372 | 17653 | 1.19915752291281 | 0.0325800125631282 | 0.802770286708069 | 1.48704875255985 | 0.239247311827957 | KIT//PRKAA1//THBS1//CD81//TSLP//MAS1//DBNL//SYT9//PPP3CB//ABCC8//RGS5//FGFR2//VAV3//EDN1//FGF11//BMP10//GRP//NDP//IL26//CCL16//TNFSF14//CXCL14//AKAP7//TTK//PIP4K2C//PIK3IP1//MGRN1//YWHAB//NREP//SMPD1//FOXP3//SFRP1//TRABD2B//RASL11B//ITGA5//USP46//NLGN1//CA7//NPVF//RALGPS2//RIT2//UFM1//UBQLN1//WDFY1//OGT//BCR//ARHGEF6//VSNL1//CTH//NDFIP2//NDFIP1//EEF1E1//IGFBP1//ERH//ASCL1//TSPAN14//EVC//LRRTM1//TRIM67//CPLX1//CPLX3//GRIN2B//CNTN2//GRIA3//LRP8//P2RX2//PAX5//RALBP1//TAGAP//ARHGAP40//RHOF//CGNL1//RTN4//PALM//HEYL//CYSLTR2//RBMS3//PSMC6//DAPK3//DLX5//NFKB1//TNKS2//FFAR3//LYPD6//PATE1//PTPN18//BAG5//LDOC1//PAX8// |
| GO:0050803 | regulation of synapse structure or activity | Biological process | 8 | 176 | 372 | 17653 | 2.15701368523949 | 0.0332669881692373 | 0.802770286708069 | 1.47798651625441 | 0.021505376344086 | NLGN1//LRRTM1//LRRN1//ACTR2//LRP8//GRIN2B//ZNF804A//GRIPAP1// |
| GO:0050890 | cognition | Biological process | 11 | 277 | 372 | 17653 | 1.88446682970382 | 0.0334539545322511 | 0.802770286708069 | 1.47555253768618 | 0.0295698924731183 | GRIN2B//PPP3CB//CNTN2//ITGA5//ABCC8//VLDLR//CTNS//ACTR2//KIT//RAG1//CHRM1// |
| GO:0003007 | heart morphogenesis | Biological process | 10 | 243 | 372 | 17653 | 1.9528518961016 | 0.0337465022871866 | 0.802770286708069 | 1.47177123368016 | 0.0268817204301075 | TMED2//MICAL2//FGFR2//PARVA//FZD2//HEYL//UBE4B//MYOM3//BMP10//RTN4// |
| GO:0014823 | response to activity | Biological process | 4 | 58 | 372 | 17653 | 3.27271041898406 | 0.0337776935473871 | 0.802770286708069 | 1.47137000881595 | 0.010752688172043 | EDN1//ALAD//MAS1//PRKAA1// |
| GO:0051865 | protein autoubiquitination | Biological process | 4 | 58 | 372 | 17653 | 3.27271041898406 | 0.0337776935473871 | 0.802770286708069 | 1.47137000881595 | 0.010752688172043 | UBE4B//RAG1//TRIM21//UBE2D3// |
| GO:0055025 | positive regulation of cardiac muscle tissue development | Biological process | 4 | 58 | 372 | 17653 | 3.27271041898406 | 0.0337776935473871 | 0.802770286708069 | 1.47137000881595 | 0.010752688172043 | FGFR2//BMP10//EDN1//DDX39B// |
| GO:0051716 | cellular response to stimulus | Biological process | 177 | 7556 | 372 | 17653 | 1.11162139893099 | 0.03402833841681 | 0.802770286708069 | 1.46815925672342 | 0.475806451612903 | CAMKK2//FGFR2//GRIN2B//IL5RA//KIT//NCAM1//MAPK4//PSMC6//SPTBN2//YWHAB//PRKAA1//THBS1//CD81//TSLP//CD209//NFKB1//CCL16//UBE2D3//P2RX2//TRABD2B//MARK1//NDP//TNKS2//VPS26A//FZD2//GNAO1//PPP3CB//FZD3//RALBP1//PRDM4//PTPRT//AKAP10//CHRM1//TAGAP//FGF11//GABRR1//HTR3E//GRP//KCNIP3//KCNIP2//ARHGAP40//IGFBP1//IL4R//LGALS3BP//NDFIP2//HIVEP3//BCR//RASL11B//SMPD1//ABCC8//VLDLR//LRP8//RERGL//NDFIP1//OGT//TNFSF14//SH3BP5//CXCL14//GNA14//SGK2//NUDT4//DAPK3//CARHSP1//RIT2//OXSR1//POLI//CDC5L//POLR2E//XRCC4//MGST1//VAV3//FBXO45//EDN1//CLEC1A//TSPAN14//TSPAN18//PIK3C2A//GNAZ//GPR34//FFAR3//MAS1//CYSLTR2//PTAFR//SFRP1//ARHGEF6//GPR3//PALM//GRIA3//NPVF//HEYL//ASCL1//EVC//ITGA5//TLN1//FERMT3//DBNL//DDAH1//ARFIP2//RND1//RHOF//RALGPS2//RGS5//ATP6V1G2//PTBP1//UBE4B//BMP10//IL26//PPP4R2//AKAP7//TTK//PIP4K2C//PIK3IP1//MGRN1//BRK1//NREP//LRRTM1//NDN//HLA-DPA1//FOXP3//NLGN1//UBQLN1//DLX5//CTH//EDEM3//CREB3L3//JAM3//LARP1//RABL3//RAB2A//IGFBP7//INSIG2//UFM1//WDFY1//SRXN1//TXNDC8//GPX8//TMX1//C9ORF129//MTAP//VSNL1//ACTR2//EXTL2//GOSR2//DNAJB12//UNC5A//ARPC3//PTPN18//PAX8//EEF1E1//CPS1//ERH//TRIM67//EFNA3//RTN4//PAX5//CGNL1//CNTN2//TRIM21//CANX//ALAD//SATB2//CYP7A1//ELK1//SEMA4G//PARVA//CASP7//RBMS3//BSN//LYPD6//PATE1//NUP214//BAG5//GABPA//LDOC1//FAM168A//EIF4A2//DDX39B// |
| GO:0006896 | Golgi to vacuole transport | Biological process | 2 | 14 | 372 | 17653 | 6.77918586789555 | 0.0340966957934598 | 0.802770286708069 | 1.46728770511882 | 0.00537634408602151 | EHD3//GOSR2// |
| GO:0030950 | establishment or maintenance of actin cytoskeleton polarity | Biological process | 2 | 14 | 372 | 17653 | 6.77918586789555 | 0.0340966957934598 | 0.802770286708069 | 1.46728770511882 | 0.00537634408602151 | RND1//RHOF// |
| GO:0035589 | G-protein coupled purinergic nucleotide receptor signaling pathway | Biological process | 2 | 14 | 372 | 17653 | 6.77918586789555 | 0.0340966957934598 | 0.802770286708069 | 1.46728770511882 | 0.00537634408602151 | GPR34//PTAFR// |
| GO:0044406 | adhesion of symbiont to host | Biological process | 2 | 14 | 372 | 17653 | 6.77918586789555 | 0.0340966957934598 | 0.802770286708069 | 1.46728770511882 | 0.00537634408602151 | CD209//CD81// |
| GO:0045623 | negative regulation of T-helper cell differentiation | Biological process | 2 | 14 | 372 | 17653 | 6.77918586789555 | 0.0340966957934598 | 0.802770286708069 | 1.46728770511882 | 0.00537634408602151 | IL4R//FOXP3// |
| GO:0050667 | homocysteine metabolic process | Biological process | 2 | 14 | 372 | 17653 | 6.77918586789555 | 0.0340966957934598 | 0.802770286708069 | 1.46728770511882 | 0.00537634408602151 | CTH//CPS1// |
| GO:0090178 | regulation of establishment of planar polarity involved in neural tube closure | Biological process | 2 | 14 | 372 | 17653 | 6.77918586789555 | 0.0340966957934598 | 0.802770286708069 | 1.46728770511882 | 0.00537634408602151 | FZD2//SFRP1// |
| GO:0044344 | cellular response to fibroblast growth factor stimulus | Biological process | 7 | 145 | 372 | 17653 | 2.29089729328884 | 0.0340988551959765 | 0.802770286708069 | 1.46726020137161 | 0.0188172043010753 | FGFR2//POLR2E//PTBP1//THBS1//CPS1//SFRP1//CDC5L// |
| GO:0050793 | regulation of developmental process | Biological process | 67 | 2563 | 372 | 17653 | 1.24051430824932 | 0.0342954934306317 | 0.802770286708069 | 1.46476294429525 | 0.18010752688172 | OTP//FZD3//FOXP3//NDFIP1//DAPK3//GAS2//RND1//KIT//PALM//RHOF//PARVA//FGFR2//SFRP1//NFKB1//ZNF804A//TRIM67//RIT2//BAG5//SEPT7//EFNA3//ABCC8//THBS1//TTL//RTN4//ASCL1//CNTN2//TNKS2//EDN1//HMGB3//RAG1//ZNF268//TAF8//FAM57B//IL4R//H3F3B//KMT2E//GABPA//CXCL14//TNFSF14//NLGN1//NREP//HEYL//NAB1//DDAH1//CYSLTR2//PRKAA1//SEMA4G//TLX2//PTBP1//LRRTM1//LRRN1//BMP10//FZD2//PSMC6//PLAG1//BCR//ACTR2//LRP8//DDX39B//PAX8//PIK3C2A//VLDLR//ZNF322//ZSWIM6//ITGA5//CTH//EEF1E1// |
| GO:0045987 | positive regulation of smooth muscle contraction | Biological process | 3 | 34 | 372 | 17653 | 4.18714421252372 | 0.0343043934435357 | 0.802770286708069 | 1.46465025528974 | 0.00806451612903226 | KIT//PTAFR//EDN1// |
| GO:1901615 | organic hydroxy compound metabolic process | Biological process | 17 | 497 | 372 | 17653 | 1.62318534865105 | 0.0350304407202538 | 0.812779818693812 | 1.4555543987608 | 0.0456989247311828 | SLC5A3//MOGAT2//INSIG2//PRKAA1//CYP7A1//VLDLR//NFKB1//GPD2//PTAFR//CYP4F3//CYP4F2//CTNS//NUDT4//NFYA//MAS1//KIT//PAX8// |
| GO:0006029 | proteoglycan metabolic process | Biological process | 5 | 86 | 372 | 17653 | 2.75897099274819 | 0.035212847386853 | 0.812779818693812 | 1.45329885552057 | 0.0134408602150538 | EXTL2//XYLT2//NDST2//NDST3//HS6ST1// |
| GO:0048661 | positive regulation of smooth muscle cell proliferation | Biological process | 5 | 86 | 372 | 17653 | 2.75897099274819 | 0.035212847386853 | 0.812779818693812 | 1.45329885552057 | 0.0134408602150538 | DDX39B//EDN1//FGFR2//PTAFR//THBS1// |
| GO:1901655 | cellular response to ketone | Biological process | 5 | 86 | 372 | 17653 | 2.75897099274819 | 0.035212847386853 | 0.812779818693812 | 1.45329885552057 | 0.0134408602150538 | PRKAA1//SFRP1//ELK1//LARP1//CDC5L// |
| GO:0031344 | regulation of cell projection organization | Biological process | 20 | 612 | 372 | 17653 | 1.55079415278656 | 0.0355738315587077 | 0.813038585305347 | 1.44886935564113 | 0.0537634408602151 | BRK1//SFRP1//ZNF804A//TRIM67//RIT2//BAG5//TTL//RTN4//CNTN2//KIT//SEMA4G//TLX2//NLGN1//PALM//ACTR2//LRP8//VLDLR//ZSWIM6//SEPT7//GRIN2B// |
| GO:0071806 | protein transmembrane transport | Biological process | 4 | 59 | 372 | 17653 | 3.21724075086568 | 0.0356538130879849 | 0.813038585305347 | 1.44789401659711 | 0.010752688172043 | PEX5//TIMM23//TOMM40L//BCR// |
| GO:0019751 | polyol metabolic process | Biological process | 6 | 116 | 372 | 17653 | 2.45453281423804 | 0.0361837333959631 | 0.813038585305347 | 1.44148662515957 | 0.0161290322580645 | SLC5A3//MOGAT2//GPD2//PTAFR//NUDT4//MAS1// |
| GO:0006986 | response to unfolded protein | Biological process | 8 | 179 | 372 | 17653 | 2.12086261788911 | 0.0362145110096852 | 0.813038585305347 | 1.44111737453145 | 0.021505376344086 | CTH//EDEM3//CREB3L3//EXTL2//TLN1//GOSR2//IGFBP1//THBS1// |
| GO:0048013 | ephrin receptor signaling pathway | Biological process | 5 | 87 | 372 | 17653 | 2.72725868248671 | 0.0367407139356041 | 0.813038585305347 | 1.43485240885545 | 0.0134408602150538 | ARPC3//ACTR2//VAV3//EFNA3//GRIN2B// |
| GO:0048644 | muscle organ morphogenesis | Biological process | 5 | 87 | 372 | 17653 | 2.72725868248671 | 0.0367407139356041 | 0.813038585305347 | 1.43485240885545 | 0.0134408602150538 | FZD2//UBE4B//MYOM3//BMP10//FGFR2// |
| GO:0060021 | roof of mouth development | Biological process | 5 | 87 | 372 | 17653 | 2.72725868248671 | 0.0367407139356041 | 0.813038585305347 | 1.43485240885545 | 0.0134408602150538 | FZD2//DLX5//SATB2//ASPH//INSIG2// |
| GO:0032502 | developmental process | Biological process | 149 | 6267 | 372 | 17653 | 1.12824172015559 | 0.036749921579684 | 0.813038585305347 | 1.43474358331575 | 0.400537634408602 | DLX5//EVC//VAV3//FGFR2//ITGA5//RTN4//JAM3//HS6ST1//KIT//EDN1//IARS//TP53INP2//SFRP1//PAX8//TXNDC8//GABPA//BRK1//TLX2//FZD3//SEMA4G//FBXO45//SATB2//MARK1//ASCL1//NDN//TAF8//TMED2//DDX39B//NDP//LDOC1//PROX2//PPP3CB//MICAL2//NAB1//PARVA//THBS1//OTP//CTNS//RAG1//FOXP3//NDFIP1//FZD2//HEYL//UBE4B//GRIN2B//HMGB3//PAX5//ASF1B//HIVEP3//FOXG1//MAS1//ASPH//CHRM1//ELAVL3//FGF11//NLGN1//KIF2A//SCN3B//PURA//SMPD1//VLDLR//LRRC38//LRRN1//PARD6B//EFNA3//NCAM1//SPTBN2//CNTN2//UNC5A//DBNL//BSN//NPTX1//H3F3B//CA10//BCR//SLC5A3//CPS1//MEST//BMP10//PAXBP1//IGFBP7//GNAO1//IGFBP1//CANX//DAPK3//GAS2//RND1//PALM//RHOF//KRT9//RNF38//MEA1//HOXC9//ZNF281//NFKB1//ZNF804A//TRIM67//RIT2//BAG5//SEPT7//ABCC8//LRP8//SYPL2//PLAG1//ZNF268//ELK1//CDC5L//KDELR1//KMT2E//MYOM3//TTL//NREP//TBC1D24//PRDM12//KRTAP1-1//KRT38//MGST1//FERMT3//POLR2E//GPD2//TNKS2//INSIG2//KCNQ4//MAEA//SLC27A4//TET3//KCNIP2//C9ORF129//FAM57B//IL4R//CXCL14//TNFSF14//DDAH1//CYSLTR2//C2ORF49//PRKAA1//P2RX2//MSI2//PTBP1//LRRTM1//PSMC6//CRISPLD1//ACTR2//PIK3C2A//ZNF322//ZSWIM6//MCIDAS//CTH//EEF1E1// |
| GO:0045184 | establishment of protein localization | Biological process | 54 | 2012 | 372 | 17653 | 1.27362438273584 | 0.0368356177112809 | 0.813038585305347 | 1.43373204288928 | 0.145161290322581 | SPRN//NUP214//DDX39B//NLGN1//PAN3//YWHAB//ZDHHC20//GOLGA7B//GOSR2//TIMM23//TOMM34//KDELR1//TMED2//VPS45//AP1S1//ARFIP2//RABL3//KCNIP3//RAB2A//MLPH//VPS26A//SYT9//PPP3CB//ABCC8//CANX//YKT6//EHD3//VPS53//ANKRD50//ZW10//PEX5//CPLX1//CPLX3//TOMM40L//REP15//ATP6V1G2//VLDLR//VSNL1//SYS1//SFRP1//FOXP3//CLEC9A//IL26//NDFIP2//NDFIP1//BCR//CNTN2//IL4R//CHRM1//GRIPAP1//NUCB1//PRKAA1//UBE2D3//TSLP// |
| GO:0002707 | negative regulation of lymphocyte mediated immunity | Biological process | 3 | 35 | 372 | 17653 | 4.06751152073733 | 0.0369517467138272 | 0.813038585305347 | 1.43236502762783 | 0.00806451612903226 | PPP3CB//FOXP3//NDFIP1// |
| GO:0032369 | negative regulation of lipid transport | Biological process | 3 | 35 | 372 | 17653 | 4.06751152073733 | 0.0369517467138272 | 0.813038585305347 | 1.43236502762783 | 0.00806451612903226 | THBS1//CYP4F2//NFKB1// |
| GO:0060070 | canonical Wnt signaling pathway | Biological process | 12 | 317 | 372 | 17653 | 1.79637732777043 | 0.037079955489866 | 0.813038585305347 | 1.43086079584677 | 0.032258064516129 | RBMS3//IGFBP1//PSMC6//SFRP1//DAPK3//DLX5//FGFR2//NFKB1//TNKS2//FZD2//NDP//FZD3// |
| GO:0016043 | cellular component organization | Biological process | 149 | 6269 | 372 | 17653 | 1.12788177703223 | 0.0371262169489818 | 0.813038585305347 | 1.43031930183458 | 0.400537634408602 | UBXN2B//UBQLN1//TP53INP2//ZW10//SEPT7//H3F3B//TTL//KIF2A//MARK1//MAP7D1//CNTN2//DDX39B//SGK2//IGFBP7//TXNDC8//LRRTM1//ANP32E//RND1//RHOF//MICAL2//ARFIP2//BCR//MAEA//PALM//PFDN1//SPTBN2//CCDC6//NDP//VAV3//YKT6//SYT9//PURA//DAPK3//ASF1B//SATB2//HMGB3//FOXP3//TIMM23//TOMM34//GABPA//MYOM3//BRK1//CGNL1//TLN1//TMED2//RAB2A//BAG5//PARD6B//ITGA5//TTK//MAPRE1//NUP214//DPEP3//LRRC38//FGFR2//RTN4//LRRN1//EFNA3//NCAM1//VLDLR//UNC5A//NDN//NLGN1//DBNL//BSN//P2RX2//GAS2//KIT//PARVA//ACTR2//UBXN10//EHD3//MCIDAS//RAG1//UBE2E1//PAN3//SFRP1//ZNF804A//TRIM67//RIT2//PEX5//FBXO45//ARHGEF6//TOMM40L//THBS1//JAM3//EIF4G2//IGFBP1//BMP10//ARHGAP40//PAXBP1//NREP//SKA3//UBE4B//GNAO1//TBC1D24//PRDM12//TNKS2//TRABD2B//NDUFA12//FERMT3//ARPC3//DNAJB12//KMT2E//PRKAA1//FZD3//GPR3//KCTD18//ALAD//KCNA6//GATAD2B//OGT//PRDM4//KRT9//KCNIP2//EDN1//PSMC6//CEP85//GOSR2//PIK3C2A//PPP3CB//SEMA4G//TLX2//CTH//DPYS//GRIN2B//YWHAB//PAX5//NPTX1//LRP8//TGOLN2//ANO3//PPIH//TRIM21//MGST1//PROX2//TET3//ATP6V1G2//DLX5//GRIPAP1//IL4R//TNFSF14//ASPH//ZSWIM6//CAMKK2//UBE2D3//FZD2//ABCC8//PIP4K2C// |
| GO:0006479 | protein methylation | Biological process | 8 | 180 | 372 | 17653 | 2.10908004778973 | 0.0372349349944103 | 0.813038585305347 | 1.42904940002106 | 0.021505376344086 | METTL21A//PAXBP1//KMT2E//PRDM4//PAX5//PRDM12//OGT//TET3// |
| GO:0008213 | protein alkylation | Biological process | 8 | 180 | 372 | 17653 | 2.10908004778973 | 0.0372349349944103 | 0.813038585305347 | 1.42904940002106 | 0.021505376344086 | METTL21A//PAXBP1//KMT2E//PRDM4//PAX5//PRDM12//OGT//TET3// |
| GO:0071242 | cellular response to ammonium ion | Biological process | 4 | 60 | 372 | 17653 | 3.16362007168459 | 0.0375890954865846 | 0.813038585305347 | 1.42493812466957 | 0.010752688172043 | CHRM1//CASP7//GABPA//PTAFR// |
| GO:0048731 | system development | Biological process | 116 | 4760 | 372 | 17653 | 1.15644935393512 | 0.0378271936670687 | 0.813038585305347 | 1.42219587705552 | 0.311827956989247 | DLX5//EVC//VAV3//FGFR2//ITGA5//RTN4//JAM3//HS6ST1//KIT//EDN1//PAX8//SFRP1//SEMA4G//FBXO45//SATB2//MARK1//ASCL1//NDN//FZD3//TMED2//DDX39B//NDP//LDOC1//PROX2//PPP3CB//MICAL2//NAB1//PARVA//THBS1//OTP//CTNS//RAG1//FOXP3//NDFIP1//FZD2//HEYL//UBE4B//PAX5//CHRM1//ELAVL3//FGF11//NLGN1//KIF2A//SCN3B//PURA//SMPD1//VLDLR//LRRC38//LRRN1//PARD6B//EFNA3//NCAM1//SPTBN2//CNTN2//UNC5A//DBNL//BSN//NPTX1//FOXG1//GRIN2B//H3F3B//CA10//BCR//SLC5A3//CPS1//BMP10//PAXBP1//RNF38//MAS1//MEA1//ZNF804A//TRIM67//RIT2//BAG5//RND1//ABCC8//LRP8//SYPL2//PLAG1//DAPK3//KDELR1//KMT2E//TTL//GNAO1//NREP//TBC1D24//PRDM12//KRT9//KRTAP1-1//KRT38//MGST1//HIVEP3//INSIG2//KCNQ4//GPD2//MAEA//SLC27A4//KCNIP2//HMGB3//IL4R//GABPA//DDAH1//CYSLTR2//TLX2//PRKAA1//HOXC9//MYOM3//P2RX2//CXCL14//LRRTM1//PSMC6//PALM//ACTR2//PIK3C2A//ZSWIM6//CTH// |
| GO:0009914 | hormone transport | Biological process | 12 | 318 | 372 | 17653 | 1.79072834246297 | 0.0378359481691784 | 0.813038585305347 | 1.42209537813668 | 0.032258064516129 | SYT9//PPP3CB//ABCC8//EDN1//CPLX1//CPLX3//NPVF//VSNL1//SFRP1//FFAR3//GRP//PAX8// |
| GO:0007411 | axon guidance | Biological process | 10 | 248 | 372 | 17653 | 1.91347988206729 | 0.0379467705561461 | 0.813038585305347 | 1.42082517864398 | 0.0268817204301075 | UNC5A//FZD3//SEMA4G//DLX5//ZSWIM6//EFNA3//NCAM1//SPTBN2//CNTN2//VLDLR// |
| GO:0034620 | cellular response to unfolded protein | Biological process | 7 | 149 | 372 | 17653 | 2.22939669481129 | 0.0386297647832653 | 0.813038585305347 | 1.41307793626732 | 0.0188172043010753 | CTH//EDEM3//CREB3L3//EXTL2//TLN1//GOSR2//IGFBP1// |
| GO:0001946 | lymphangiogenesis | Biological process | 2 | 15 | 372 | 17653 | 6.32724014336918 | 0.0388041440307348 | 0.813038585305347 | 1.41112189209709 | 0.00537634408602151 | PROX2//PPP3CB// |
| GO:0010919 | regulation of inositol phosphate biosynthetic process | Biological process | 2 | 15 | 372 | 17653 | 6.32724014336918 | 0.0388041440307348 | 0.813038585305347 | 1.41112189209709 | 0.00537634408602151 | MAS1//PTAFR// |
| GO:0031033 | myosin filament organization | Biological process | 2 | 15 | 372 | 17653 | 6.32724014336918 | 0.0388041440307348 | 0.813038585305347 | 1.41112189209709 | 0.00537634408602151 | MYOM3//DAPK3// |
| GO:0032305 | positive regulation of icosanoid secretion | Biological process | 2 | 15 | 372 | 17653 | 6.32724014336918 | 0.0388041440307348 | 0.813038585305347 | 1.41112189209709 | 0.00537634408602151 | EDN1//CYP4F2// |
| GO:0043371 | negative regulation of CD4-positive, alpha-beta T cell differentiation | Biological process | 2 | 15 | 372 | 17653 | 6.32724014336918 | 0.0388041440307348 | 0.813038585305347 | 1.41112189209709 | 0.00537634408602151 | IL4R//FOXP3// |
| GO:0050774 | negative regulation of dendrite morphogenesis | Biological process | 2 | 15 | 372 | 17653 | 6.32724014336918 | 0.0388041440307348 | 0.813038585305347 | 1.41112189209709 | 0.00537634408602151 | NLGN1//TLX2// |
| GO:0071599 | otic vesicle development | Biological process | 2 | 15 | 372 | 17653 | 6.32724014336918 | 0.0388041440307348 | 0.813038585305347 | 1.41112189209709 | 0.00537634408602151 | FGFR2//PAX8// |
| GO:0090177 | establishment of planar polarity involved in neural tube closure | Biological process | 2 | 15 | 372 | 17653 | 6.32724014336918 | 0.0388041440307348 | 0.813038585305347 | 1.41112189209709 | 0.00537634408602151 | FZD2//SFRP1// |
| GO:1903818 | positive regulation of voltage-gated potassium channel activity | Biological process | 2 | 15 | 372 | 17653 | 6.32724014336918 | 0.0388041440307348 | 0.813038585305347 | 1.41112189209709 | 0.00537634408602151 | LRRC38//ABCC8// |
| GO:0018022 | peptidyl-lysine methylation | Biological process | 6 | 118 | 372 | 17653 | 2.41293056314926 | 0.0388306170417902 | 0.813038585305347 | 1.41082570819937 | 0.0161290322580645 | KMT2E//PAX5//PRDM12//OGT//TET3//METTL21A// |
| GO:0010720 | positive regulation of cell development | Biological process | 17 | 504 | 372 | 17653 | 1.60064110769756 | 0.0391387518552729 | 0.816961045670403 | 1.4073930282184 | 0.0456989247311828 | OTP//FZD3//TRIM67//RIT2//ZNF804A//HEYL//ASCL1//KIT//RTN4//PLAG1//EDN1//BMP10//NLGN1//ACTR2//LRP8//DDX39B//VLDLR// |
| GO:0046622 | positive regulation of organ growth | Biological process | 4 | 61 | 372 | 17653 | 3.11175744755861 | 0.0395836231734863 | 0.820545916216811 | 1.40248445640967 | 0.010752688172043 | FGFR2//BMP10//EDN1//DDX39B// |
| GO:0060323 | head morphogenesis | Biological process | 3 | 36 | 372 | 17653 | 3.95452508960574 | 0.0397007377243536 | 0.820545916216811 | 1.40120142304446 | 0.00806451612903226 | DLX5//ASPH//CRISPLD1// |
| GO:0097485 | neuron projection guidance | Biological process | 10 | 250 | 372 | 17653 | 1.89817204301075 | 0.0397238650706514 | 0.820545916216811 | 1.4009485019392 | 0.0268817204301075 | EFNA3//NCAM1//SPTBN2//CNTN2//VLDLR//UNC5A//FZD3//SEMA4G//DLX5//ZSWIM6// |
| GO:0007044 | cell-substrate junction assembly | Biological process | 5 | 89 | 372 | 17653 | 2.66597197052072 | 0.0399171383166244 | 0.820545916216811 | 1.39884060109827 | 0.0134408602150538 | DAPK3//SFRP1//THBS1//ITGA5//TLN1// |
| GO:0007193 | adenylate cyclase-inhibiting G-protein coupled receptor signaling pathway | Biological process | 5 | 89 | 372 | 17653 | 2.66597197052072 | 0.0399171383166244 | 0.820545916216811 | 1.39884060109827 | 0.0134408602150538 | CHRM1//EDN1//GNAZ//FFAR3//PALM// |
| GO:0046718 | viral entry into host cell | Biological process | 6 | 119 | 372 | 17653 | 2.39265383572784 | 0.0401990507486625 | 0.823836909736983 | 1.39578420212661 | 0.0161290322580645 | TRIM21//TMPRSS2//CD209//ITGA5//NCAM1//CD81// |
| GO:0090596 | sensory organ morphogenesis | Biological process | 10 | 251 | 372 | 17653 | 1.89060960459238 | 0.0406335493840079 | 0.830225663093793 | 1.39111523992902 | 0.0268817204301075 | FGFR2//DLX5//INSIG2//BCR//PAX8//FZD3//KCNQ4//EDN1//PROX2//FZD2// |
| GO:0030029 | actin filament-based process | Biological process | 23 | 739 | 372 | 17653 | 1.47692682642921 | 0.0407888537882619 | 0.830888608945829 | 1.38945849866494 | 0.0618279569892473 | RND1//RHOF//MICAL2//ARFIP2//BCR//MYOM3//BRK1//CGNL1//MLPH//ARHGAP40//TLN1//KIT//PARVA//ARPC3//ACTR2//DAPK3//BMP10//SFRP1//SPTBN2//EDN1//SCN3B//SCN1A//JAM3// |
| GO:0007420 | brain development | Biological process | 22 | 700 | 372 | 17653 | 1.49142089093702 | 0.0409622994172768 | 0.831916008886015 | 1.38761567224205 | 0.0591397849462366 | LRP8//PAX5//SPTBN2//ASCL1//SYPL2//MAS1//FGFR2//FBXO45//RTN4//CNTN2//OTP//DLX5//SATB2//GNAO1//FZD3//SFRP1//CTNS//FOXG1//GRIN2B//H3F3B//CA10//BCR// |
| GO:1905269 | positive regulation of chromatin organization | Biological process | 5 | 90 | 372 | 17653 | 2.63635005973716 | 0.0415660235214546 | 0.841649751723346 | 1.38126152096632 | 0.0134408602150538 | PAXBP1//FOXP3//PRDM12//OGT//KMT2E// |
| GO:0048598 | embryonic morphogenesis | Biological process | 19 | 586 | 372 | 17653 | 1.53862068332783 | 0.0425261862177504 | 0.852694018675678 | 1.37134356341467 | 0.0510752688172043 | TLX2//TMED2//FZD3//MICAL2//ZNF281//FGFR2//DLX5//ITGA5//INSIG2//BCR//PAX8//KCNQ4//EDN1//PAX5//SATB2//HOXC9//FZD2//SFRP1//C2ORF49// |
| GO:0061001 | regulation of dendritic spine morphogenesis | Biological process | 3 | 37 | 372 | 17653 | 3.8476460331299 | 0.0425504675811832 | 0.852694018675678 | 1.37109566315138 | 0.00806451612903226 | NLGN1//ACTR2//LRP8// |
| GO:0006066 | alcohol metabolic process | Biological process | 12 | 324 | 372 | 17653 | 1.75756670649144 | 0.0426015349601076 | 0.852694018675678 | 1.37057475271195 | 0.032258064516129 | SLC5A3//MOGAT2//INSIG2//PRKAA1//CYP7A1//VLDLR//NFKB1//GPD2//PTAFR//NUDT4//NFYA//MAS1// |
| GO:0003231 | cardiac ventricle development | Biological process | 6 | 121 | 372 | 17653 | 2.35310583844308 | 0.0430266663564936 | 0.852694018675678 | 1.36626230111856 | 0.0161290322580645 | FGFR2//FZD2//HEYL//UBE4B//PAX8//BMP10// |
| GO:0120034 | positive regulation of plasma membrane bounded cell projection assembly | Biological process | 5 | 91 | 372 | 17653 | 2.60737917995983 | 0.043255543044132 | 0.852694018675678 | 1.36395823133839 | 0.0134408602150538 | BRK1//KIT//NLGN1//PALM//SEPT7// |
| GO:0007517 | muscle organ development | Biological process | 14 | 398 | 372 | 17653 | 1.66924677149187 | 0.0433592538213529 | 0.852694018675678 | 1.36291820027173 | 0.0376344086021505 | FZD2//UBE4B//HEYL//PAX5//HIVEP3//PRKAA1//P2RX2//MYOM3//BMP10//FGFR2//EDN1//DDX39B//EVC//PAXBP1// |
| GO:0007416 | synapse assembly | Biological process | 7 | 153 | 372 | 17653 | 2.17111181390119 | 0.0435385620077997 | 0.852694018675678 | 1.36112591894193 | 0.0188172043010753 | NLGN1//LRRTM1//LRRN1//FBXO45//DBNL//SPTBN2//BSN// |
| GO:0032695 | negative regulation of interleukin-12 production | Biological process | 2 | 16 | 372 | 17653 | 5.9317876344086 | 0.0437420347958806 | 0.852694018675678 | 1.35910101827899 | 0.00537634408602151 | NFKB1//THBS1// |
| GO:0034315 | regulation of Arp2/3 complex-mediated actin nucleation | Biological process | 2 | 16 | 372 | 17653 | 5.9317876344086 | 0.0437420347958806 | 0.852694018675678 | 1.35910101827899 | 0.00537634408602151 | BRK1//ARFIP2// |
| GO:0043584 | nose development | Biological process | 2 | 16 | 372 | 17653 | 5.9317876344086 | 0.0437420347958806 | 0.852694018675678 | 1.35910101827899 | 0.00537634408602151 | DLX5//ASCL1// |
| GO:0140131 | positive regulation of lymphocyte chemotaxis | Biological process | 2 | 16 | 372 | 17653 | 5.9317876344086 | 0.0437420347958806 | 0.852694018675678 | 1.35910101827899 | 0.00537634408602151 | TNFSF14//CXCL14// |
| GO:2000193 | positive regulation of fatty acid transport | Biological process | 2 | 16 | 372 | 17653 | 5.9317876344086 | 0.0437420347958806 | 0.852694018675678 | 1.35910101827899 | 0.00537634408602151 | CYP4F2//EDN1// |
| GO:0002260 | lymphocyte homeostasis | Biological process | 4 | 63 | 372 | 17653 | 3.01297149684246 | 0.043750528534742 | 0.852694018675678 | 1.35901669605114 | 0.010752688172043 | FOXP3//PPP3CB//RAG1//TNFSF14// |
| GO:0006979 | response to oxidative stress | Biological process | 15 | 436 | 372 | 17653 | 1.63260210121338 | 0.0439427268589388 | 0.853978913066101 | 1.35711299650416 | 0.0403225806451613 | EDN1//SRXN1//TXNDC8//GPX8//CAMKK2//GNAO1//PRKAA1//MGST1//UBQLN1//BAG5//SGK2//ALAD//NDUFA12//OXSR1//SLC23A2// |
| GO:1903522 | regulation of blood circulation | Biological process | 11 | 290 | 372 | 17653 | 1.79999073044123 | 0.0441551695245675 | 0.855648743537679 | 1.3550184435119 | 0.0295698924731183 | CHRM1//GNAO1//KCNIP2//EDN1//SCN3B//PTAFR//BMP10//EHD3//KCNIP3//CACNG1//ASPH// |
| GO:0006812 | cation transport | Biological process | 32 | 1109 | 372 | 17653 | 1.36928551344328 | 0.0442896702900415 | 0.855802971918716 | 1.35369755276443 | 0.0860215053763441 | EDN1//KCNIP2//KCNA6//ABCC8//KCNQ4//SCN3B//SCN1A//SLC5A3//SLC23A2//NDFIP2//NDFIP1//SLC39A2//ATP1B4//P2RX2//SLC9B1//STEAP2//REP15//ATP6V1G2//BEST1//GNAO1//CACNG1//SLC30A6//LRRC38//KCNIP3//GRIN2B//HTR3E//RALBP1//OXSR1//UBQLN1//EHD3//SESTD1//NLGN1// |
| GO:0010033 | response to organic substance | Biological process | 81 | 3218 | 372 | 17653 | 1.19446811283306 | 0.0447931863189991 | 0.861241073682472 | 1.34878804334812 | 0.217741935483871 | TSLP//THBS1//CHRM1//IGFBP1//PIK3C2A//ATP6V1G2//FGFR2//POLR2E//PTBP1//STEAP2//H3F3B//P2RX2//MGST1//ALAD//SFRP1//CPS1//NREP//LRRTM1//IL4R//IL5RA//KIT//NDN//IL26//PTAFR//LRP8//UBE4B//UBQLN1//PSMC6//DLX5//BMP10//UBE2D3//RASL11B//ITGA5//CTH//EDEM3//CREB3L3//PRKAA1//EDN1//ABCC8//IGFBP7//ASCL1//INSIG2//OGT//UFM1//TNFSF14//GNAO1//PPP3CB//TRIM21//MAS1//MTAP//VSNL1//ACTR2//EXTL2//TLN1//GOSR2//DNAJB12//SMPD1//CDC5L//GRIN2B//USP46//HLA-DPA1//NCAM1//HEYL//CCL16//CANX//NFKB1//BCR//SATB2//MAPK4//CYP7A1//PTPN18//DAPK3//PAX8//ELK1//FFAR3//CD81//CASP7//LARP1//GABPA//ARPC3//EIF4A2// |
| GO:0002695 | negative regulation of leukocyte activation | Biological process | 7 | 154 | 372 | 17653 | 2.15701368523949 | 0.0448257959391143 | 0.861241073682472 | 1.3484719902455 | 0.0188172043010753 | FOXP3//NDFIP1//BCR//HMGB3//SFRP1//IL4R//TNFAIP8L2// |
| GO:0000096 | sulfur amino acid metabolic process | Biological process | 3 | 38 | 372 | 17653 | 3.7463921901528 | 0.0454999352098121 | 0.866805807954815 | 1.34198922176137 | 0.00806451612903226 | CTH//MTAP//CPS1// |
| GO:0016601 | Rac protein signal transduction | Biological process | 3 | 38 | 372 | 17653 | 3.7463921901528 | 0.0454999352098121 | 0.866805807954815 | 1.34198922176137 | 0.00806451612903226 | OGT//DBNL//BRK1// |
| GO:0046676 | negative regulation of insulin secretion | Biological process | 3 | 38 | 372 | 17653 | 3.7463921901528 | 0.0454999352098121 | 0.866805807954815 | 1.34198922176137 | 0.00806451612903226 | SFRP1//ABCC8//VSNL1// |
| GO:0060359 | response to ammonium ion | Biological process | 6 | 123 | 372 | 17653 | 2.31484395489116 | 0.0459761778491244 | 0.873284509160939 | 1.33746713589472 | 0.0161290322580645 | CHRM1//SMPD1//GNAO1//CASP7//GABPA//PTAFR// |
| GO:0044272 | sulfur compound biosynthetic process | Biological process | 8 | 188 | 372 | 17653 | 2.01933196064974 | 0.0460982655286789 | 0.873284509160939 | 1.33631541485969 | 0.021505376344086 | ST3GAL3//CTH//MTAP//XYLT2//NDST2//NDST3//SLC26A1//MGST1// |
| GO:0006457 | protein folding | Biological process | 10 | 257 | 372 | 17653 | 1.84647085895988 | 0.0463927499069432 | 0.876408289443176 | 1.33354988413104 | 0.0268817204301075 | CANX//DNAJB12//BAG5//PPIH//TOMM34//GNAO1//GNAZ//PFDN1//SGTB//TMX1// |
| GO:0030219 | megakaryocyte differentiation | Biological process | 5 | 93 | 372 | 17653 | 2.55130650942305 | 0.0467569252951884 | 0.880827536967574 | 1.33015405549218 | 0.0134408602150538 | KIT//H3F3B//KMT2E//THBS1//GABPA// |
| GO:0003206 | cardiac chamber morphogenesis | Biological process | 6 | 124 | 372 | 17653 | 2.29617585848075 | 0.0474969423410105 | 0.881870350252787 | 1.32333434757869 | 0.0161290322580645 | FGFR2//PARVA//FZD2//HEYL//UBE4B//BMP10// |
| GO:1901616 | organic hydroxy compound catabolic process | Biological process | 4 | 65 | 372 | 17653 | 2.920264681555 | 0.0481543384000791 | 0.881870350252787 | 1.31736457960201 | 0.010752688172043 | CYP7A1//GPD2//CYP4F3//CYP4F2// |
| GO:0071310 | cellular response to organic substance | Biological process | 68 | 2656 | 372 | 17653 | 1.21494445524032 | 0.0481717626269776 | 0.881870350252787 | 1.31720746235974 | 0.182795698924731 | TSLP//CHRM1//IGFBP1//PIK3C2A//ATP6V1G2//FGFR2//POLR2E//PTBP1//NREP//LRRTM1//IL4R//IL5RA//KIT//NDN//IL26//PTAFR//LRP8//DLX5//BMP10//UBE2D3//THBS1//RASL11B//SFRP1//ITGA5//CTH//EDEM3//CREB3L3//IGFBP7//UFM1//PSMC6//TNFSF14//MTAP//PPP3CB//VSNL1//ACTR2//EXTL2//TLN1//GOSR2//MAS1//CPS1//CDC5L//OGT//HLA-DPA1//NCAM1//TRIM21//HEYL//CCL16//EDN1//CANX//NFKB1//DNAJB12//BCR//CYP7A1//PTPN18//DAPK3//ALAD//PRKAA1//PAX8//ELK1//FFAR3//CASP7//LARP1//GABPA//ARPC3//EIF4A2//SATB2//MAPK4//ABCC8// |
| GO:0015682 | ferric iron transport | Biological process | 3 | 39 | 372 | 17653 | 3.65033085194375 | 0.0485480440051675 | 0.881870350252787 | 1.31382826307575 | 0.00806451612903226 | REP15//ATP6V1G2//STEAP2// |
| GO:0072512 | trivalent inorganic cation transport | Biological process | 3 | 39 | 372 | 17653 | 3.65033085194375 | 0.0485480440051675 | 0.881870350252787 | 1.31382826307575 | 0.00806451612903226 | REP15//ATP6V1G2//STEAP2// |
| GO:0036503 | ERAD pathway | Biological process | 5 | 94 | 372 | 17653 | 2.52416495081217 | 0.0485689599104693 | 0.881870350252787 | 1.31364119671186 | 0.0134408602150538 | UBE4B//UBQLN1//PSMC6//EDEM3//DNAJB12// |
| GO:0000097 | sulfur amino acid biosynthetic process | Biological process | 2 | 17 | 372 | 17653 | 5.58285895003163 | 0.048898622112198 | 0.881870350252787 | 1.31070337845307 | 0.00537634408602151 | CTH//MTAP// |
| GO:0002710 | negative regulation of T cell mediated immunity | Biological process | 2 | 17 | 372 | 17653 | 5.58285895003163 | 0.048898622112198 | 0.881870350252787 | 1.31070337845307 | 0.00537634408602151 | PPP3CB//FOXP3// |
| GO:0032303 | regulation of icosanoid secretion | Biological process | 2 | 17 | 372 | 17653 | 5.58285895003163 | 0.048898622112198 | 0.881870350252787 | 1.31070337845307 | 0.00537634408602151 | CYP4F2//EDN1// |
| GO:0032891 | negative regulation of organic acid transport | Biological process | 2 | 17 | 372 | 17653 | 5.58285895003163 | 0.048898622112198 | 0.881870350252787 | 1.31070337845307 | 0.00537634408602151 | THBS1//CYP4F2// |
| GO:0036315 | cellular response to sterol | Biological process | 2 | 17 | 372 | 17653 | 5.58285895003163 | 0.048898622112198 | 0.881870350252787 | 1.31070337845307 | 0.00537634408602151 | CYP7A1//LRP8// |
| GO:0036342 | post-anal tail morphogenesis | Biological process | 2 | 17 | 372 | 17653 | 5.58285895003163 | 0.048898622112198 | 0.881870350252787 | 1.31070337845307 | 0.00537634408602151 | TMED2//FZD3// |
| GO:0042249 | establishment of planar polarity of embryonic epithelium | Biological process | 2 | 17 | 372 | 17653 | 5.58285895003163 | 0.048898622112198 | 0.881870350252787 | 1.31070337845307 | 0.00537634408602151 | FZD2//SFRP1// |
| GO:0071380 | cellular response to prostaglandin E stimulus | Biological process | 2 | 17 | 372 | 17653 | 5.58285895003163 | 0.048898622112198 | 0.881870350252787 | 1.31070337845307 | 0.00537634408602151 | PRKAA1//SFRP1// |
| GO:0071636 | positive regulation of transforming growth factor beta production | Biological process | 2 | 17 | 372 | 17653 | 5.58285895003163 | 0.048898622112198 | 0.881870350252787 | 1.31070337845307 | 0.00537634408602151 | FOXP3//THBS1// |
| GO:1902043 | positive regulation of extrinsic apoptotic signaling pathway via death domain receptors | Biological process | 2 | 17 | 372 | 17653 | 5.58285895003163 | 0.048898622112198 | 0.881870350252787 | 1.31070337845307 | 0.00537634408602151 | SFRP1//THBS1// |
| GO:0034341 | response to interferon-gamma | Biological process | 8 | 191 | 372 | 17653 | 1.9876147047233 | 0.0497498585866085 | 0.888822567013791 | 1.30320814939349 | 0.021505376344086 | HLA-DPA1//NCAM1//PTAFR//TRIM21//ACTR2//DAPK3//EDN1//CCL16// |
